# Diastereoselectivity is in the Details: Minor Changes Yield Major Improvements to the Synthesis of Bedaquiline**

**DOI:** 10.1002/chem.202201311

**Published:** 2022-07-07

**Authors:** Sarah Jane Mear, Tobias Lucas, Grace P. Ahlqvist, Juliana M. S. Robey, Jule‐Philipp Dietz, Pankaj V. Khairnar, Sanjay Maity, Corshai L. Williams, David R. Snead, Ryan C. Nelson, Till Opatz, Timothy F. Jamison

**Affiliations:** ^1^ Department of Chemistry Massachusetts Institute of Technology Cambridge MA 02139 USA; ^2^ Department of Chemistry Johannes Gutenberg University Duesbergweg 10–14 55128 Mainz Germany; ^3^ Medicines for All Institute Department of Chemistry and Life Sciences Engineering Virginia Commonwealth University Richmond Virginia 23284 USA

**Keywords:** bedaquiline, continuous flow, diastereoselectivity, lithiation, nucleophilic addition

## Abstract

Bedaquiline is a crucial medicine in the global fight against tuberculosis, yet its high price places it out of reach for many patients. Herein, we describe improvements to the key industrial lithiation‐addition sequence that enable a higher yielding and therefore more economical synthesis of bedaquiline. Prioritization of mechanistic understanding and multi‐lab reproducibility led to optimized reaction conditions that feature an unusual base‐salt pairing and afford a doubling of the yield of racemic bedaquiline. We anticipate that implementation of these improvements on manufacturing scale will be facile, thereby substantially increasing the accessibility of this essential medication.

## Graphical abstract

**Bedaquiline fumarate** is a crucial drug for treatment of multidrug‐resistant tuberculosis. Improvements to the industrial synthesis are reported here that affords racemic bedaquiline in yields twice as high as those reported in patents, presenting an opportunity to dramatically improve the affordability of this drug. Close attention to reaction mechanism informed optimization of this synthesis by providing rationale for operational controls and reaction parameters that influence yield and diastereoselectivity in surprising ways.

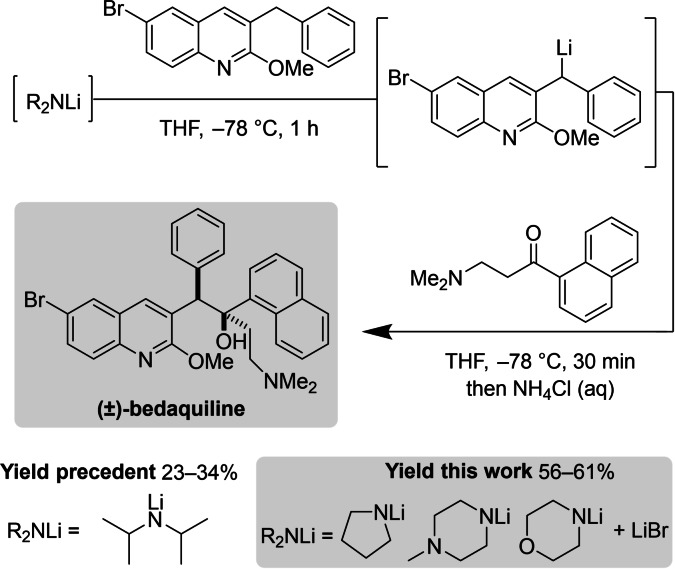

## Introduction

Tuberculosis (TB) is the result of infection by the bacillus *Mycobacterium tuberculosis* and is a leading cause of death worldwide despite the fact that it is typically curable.[^1^, ^2^] Moreover, nearly one quarter of the global population has a latent TB infection.^[2]^ The COVID‐19 pandemic has hindered diagnosis and treatment of TB, leading to an increase in TB deaths in 2020, reversing several years of progress.^[2]^ That the proportion of new cases of TB due to multi‐drug‐resistant TB (MDR‐TB) has been increasing with time further exacerbates this global health crisis. Reducing global TB burden requires a multifaceted approach, as many societal factors such as the prevalence of poverty and general access to healthcare in a country strongly influence its TB infection and mortality rate. The high price of a full TB drug regimen, particularly for MDR‐TB, remains a major factor limiting access to care.^[3]^

**Scheme 1:**
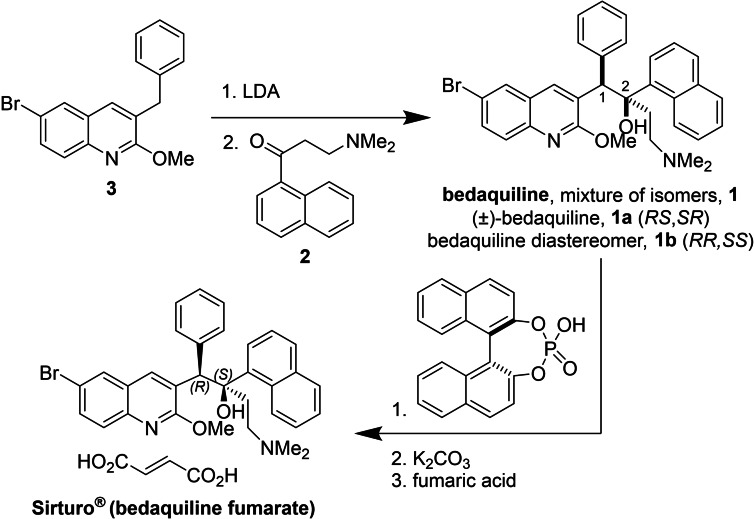
Industrial synthesis of bedaquiline fumarate.

Bedaquiline (BDQ, fumarate adduct sold under the trade name Sirturo®, Scheme 1) is an effective treatment for MDR‐TB that received FDA approval in 2012 as the first TB drug in over forty years with a novel mode of action.^[4]^ BDQ inhibits mycobacterial ATP synthase, which differentiates it from first‐line therapeutic drugs that disrupt the cell membrane or protein synthesis, to which *M. tuberculosis* commonly develops resistance.^[5]^ A key structural feature of BDQ is its two vicinal stereocenters (Scheme 1). Of the four possible stereoisomers, BDQ represents the (1*R*,2*S*)‐ or *RS*‐enantiomer. According to publicly‐available information in patent applications and the scientific literature, the industrially‐relevant construction of these stereocenters is accomplished via a 1,2‐addition reaction between quinoline fragment **3** and naphthyl ketone **2**.[^4^, ^6^, ^7^, ^8^] This reaction is facilitated by an initial deprotonation of **3** with an amide base, typically lithium diisopropylamide (LDA). A series of crystallization and chiral resolution steps are then applied to access the *RS*‐enantiomer as the penultimate intermediate to the API, which is administered as the fumarate salt.^[8]^

A prerequisite to improving global access to life‐saving medications like BDQ is lowering the cost of goods by the development of an efficient manufacturing process. Despite significant industrial interest in this reaction, patented procedures remain low‐yielding and unselective for the desired diastereomer **1 a** (Table 1). Chiral amine bases or additives can improve selectivity, but their implementation on scale may be hampered by reagent costs.[^9^, ^10^] Alternative disconnections and enantioselective routes to BDQ have also been reported, but low yields and/or high reagent costs restrict their industrial application.[^11^, ^12^] Thus, as a first step towards delivery of an improved synthesis of BDQ, we sought to understand key variables influencing the yield and diastereoselectivity of the critical coupling reaction used in the industrial process. We focused on increasing the yield of **1 a**, rather than altering the synthesis entirely, to enable use of the current industrial routes to **2** and **3** and methods for isolation of **1 a** and bedaquiline fumarate.

**Table 1:** Selected examples of synthesis of **1** reported in process patent applications and improvements described herein.

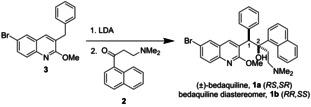			
Source	Base	Additive	Yield **1 a** [%]^[a]^
Janssen 2006^[6]^	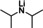	None	32 %
SIPI 2017^[7]^	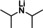	None	34 %
Mylan 2020^[8]^	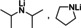	None	23 %, 14 %
This work	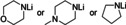	LiBr	56–61 %

Yield: yield of isolated product [a] Adjusted for purity where reported.

Herein we describe the result of this multi‐institution international collaboration, in which we present striking shortcomings of the well‐known deprotonation conditions: LDA, THF, −78 °C. An early focus on reproducibility between chemists at different research sites led to insights that informed further reaction optimization. By examining the mechanism, changing the lithium amide base, and introducing an additive, we doubled the yield of the desired diastereomer **1 a** to over 60 % while substantially maintaining the current commercial process. As our results hinge on straightforward changes to the current industrial route to BDQ, we anticipate that our findings could be rapidly implemented by manufacturers to improve access to this essential medication.

## Results and Discussion

### Establishing a Reproducible Baseline Procedure for Optimization

At the start of our investigation, we immediately encountered difficulty in reproducing reported syntheses of **1** by the reported lithiation/1,2‐addition sequence.[^6^, ^7^, ^8^] Even within our respective laboratories, we were unable to achieve similar results between different operators using the same reagents and seemingly following the same procedure. This situation is familiar to most chemists and supports the recent focus of the broader scientific community,[^13^, ^14^] as well as organic chemists in particular,^[15]^ on the importance of reproducibility for high‐quality and impactful research. Similarly, we found that our observations were due to incomplete understanding of the key variables that affect reaction outcome.

Following literature precedent, the standard protocol is to prepare **1** by first adding LDA to a stirring solution of **3** at −78 °C, followed by addition of **2** as a room‐temperature solution in THF, and finally quenching with an aqueous solution of saturated ammonium chloride. Examination of our early results showed that the largest variance in yield resulted from unintentional warming of the reaction mixture during normal experimental operations such as addition of a reagent, removal of an aliquot for analysis, or reaction quenching (Supporting Information, Table S2). To probe the detrimental effect of higher reaction temperatures on the yield of **1**, we warmed the reaction mixture to −40 °C or −20 °C after addition of **2** and observed no product formation in either case (Supporting Information, page S11). Additionally, warming to room temperature and re‐cooling to −78 °C resulted in 0 % yield of **1**.

We hypothesized that careful control of reaction temperature during reagent addition and quench would lead to more reproducible results across our research sites. Reversing the order of addition by cannulating the cold reaction mixture containing the lithium salt of **3** into a pre‐cooled solution of ketone **2** was effective for the synthesis, but this option was not investigated further as it increased operational complexity without significant improvement in yield of **1**. To avoid increase in reaction temperature during the quench, rapid addition of precooled AcOH solution instead of room temperature NH_4_Cl (aq) was explored. This improved yields of **1 a**; however, this method of quenching was problematic for isolation due to formation of insoluble precipitates, presumably the acetate salt of **1**. Ultimately, we found that quenching by slow dropwise addition of saturated NH_4_Cl (aq) solution resulted in the highest yield of **1 a**.

In addition, we found that variations in reagent quality, most notably LDA, also led to challenges with reproducibility. As has been widely reported, variations in commercial LDA solutions can lead to confounding and irreproducible results.[^16^, ^17^, ^18^] Across our research sites, a variety of commercial LDA solutions were used with varying success, which we attributed in part to differences in trace LiCl salt content of the purchased reagents.^[16]^ Freshly prepared LDA, formed by addition of *n*‐BuLi to a solution of diisopropylamine in THF, was therefore key to achieving reproducible results, as was thorough drying of all reactants and regular titration of the *n*‐BuLi solution.^[19]^ These changes were crucial to achieving high conversion of **3** and clean, reproducible 1,2‐addition using a low excess of electrophile **2**.

**Figure 1:**
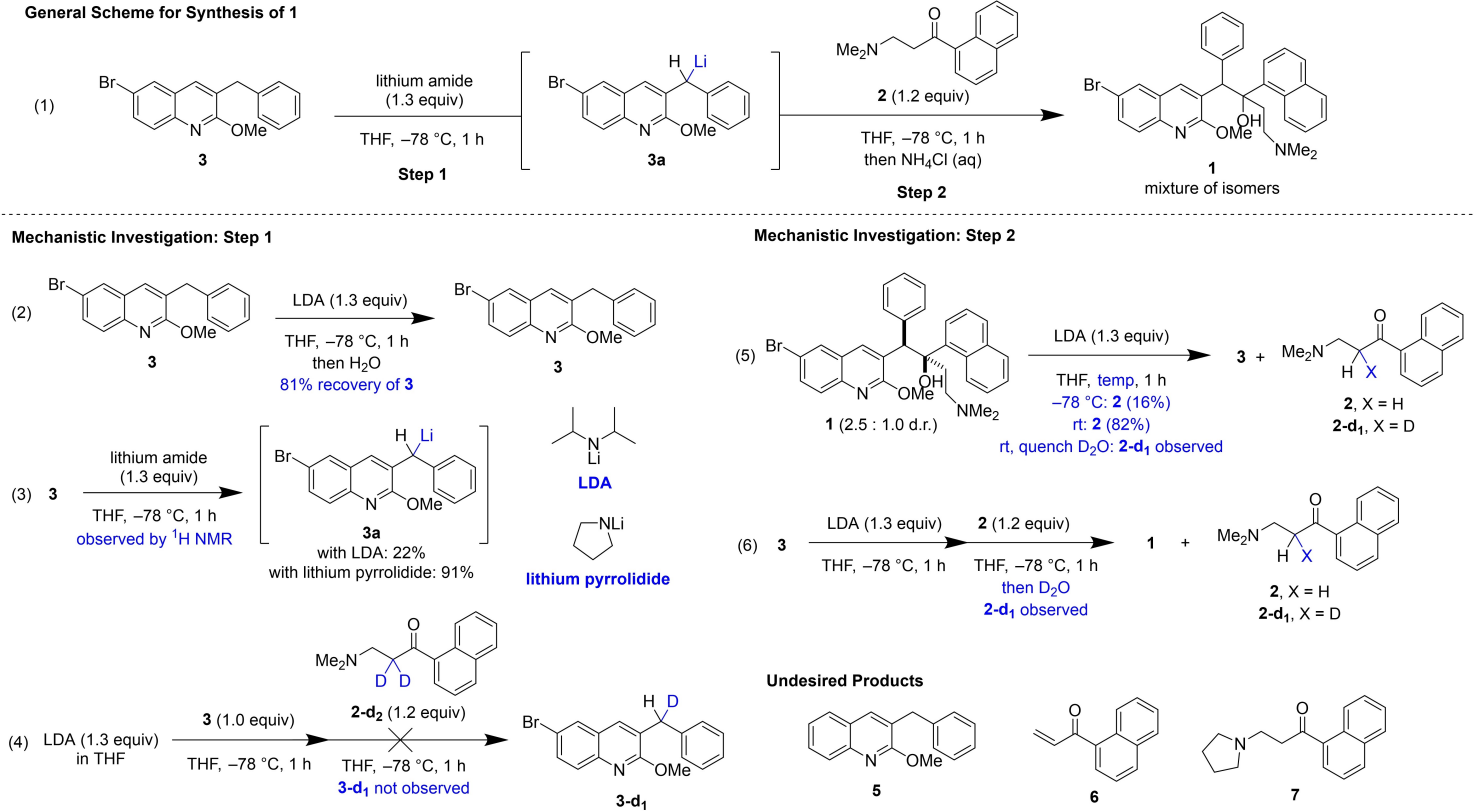
Mechanistic investigation of the lithiation/1,2‐addition sequence for synthesis of **1**.

Lastly, preparation of ketone **2** from the commercially supplied HCl salt required attention in our early optimization efforts (Supporting Information, Scheme S1). Preparation of the free base by treatment of the HCl salt with aqueous NaOH or NaHCO_3_ led to formation of elimination product **6** (Figure 1) if extended reaction times were used, or if the sample was heated during solvent removal.

With more awareness of these reproducibility challenges, we implemented a “unified procedure” (Supporting Information, page S12) that represented our baseline of reactivity from which to optimize. Once this procedure was cemented, we achieved similar results (19–25 % yield of **1 a**, 41–52 % yield of **1**) across operators at three different institutions (Supporting Information Table S3).

### Understanding the Mechanism of the Lithiation/1,2‐Addition

An early focus on reproducibility informed our understanding of the reaction mechanism and ultimately our optimization of the reaction conditions. We investigated the mechanism of this lithiation/1,2‐addition sequence with the goal of understanding how step 1 and step 2 individually contribute to the yield of **1** in this two‐step sequence. In a series of experiments described below, we demonstrate that conversion of **3** and selectivity for the desired deprotonation event in step 1 is low, and that the 1,2‐addition step is reversible. Importantly, we identified that temperature and nature of the lithium amide base are key factors in determining the reaction outcome (Figure 1).

Initially, we sought to rationalize the low (maximum ∼50 %) yield of this two‐step sequence using LDA as base. Performing step 1 followed by water quench and ^1^H NMR analysis of the crude reaction mixture, we observed low recovery of **3** (81 %), corresponding to undesired reactivity of **3** with LDA (Figure 1, Reaction 2). We identified the debrominated species **5** as a major side product, suggesting that lithium‐halogen exchange occurs competitively with benzylic deprotonation.

We then directly assayed deprotonation by observing a mixture of **3** and LDA by ^1^H NMR spectroscopy at −78 °C and identified resonances corresponding to intermediate **3 a** (Supporting Information, Figure S18). Formation of **3 a** occurred within minutes, albeit with low conversion of **3**; we were surprised to observe unreacted LDA in the presence of **3** after 15 minutes (Supporting Information, Figure S5). In a second trial, only 7 % **3 a** was observed within 10 min with 32 % consumption of **3** (Supporting Information, page S20). After warming to room temperature inside of the spectrometer over 40 min, further consumption of **3** increased to 58 %, while formation of **3 a** only increased to 11 %. This low selectivity for **3 a** is evidence for undesired reactivity of **3** with LDA. We rationalized that the deprotonation of **3** by LDA is incomplete due to the steric bulk of LDA.

While these observations around step 1 rationalized the yield we obtained with our baseline procedure using LDA, we remained perplexed by the detrimental effect of warming during reagent addition and quench for step 2. Initially, we sought to explore the possibility of a reversible sequence by treatment of **1** with LDA (Figure 1, Reaction 5). We observed formation of starting material **2** at −78 °C and at room temperature (16 % and 82 %, respectively), which aligns with observations by Kong and co‐workers who demonstrated that **1 b** can be recycled into **2** and **3** under basic conditions.^[20]^ Our observations suggest that retroaddition occurs at higher temperatures, which accounts for the variation in yield with different quenching or analysis methods (Supporting Information, Table S2). We rationalize the reversibility of this 1,2‐addition with the sterically crowded environment and entropic cost of the formation of adjacent chiral centers in **1**, factors which are exacerbated with temperature increase.^[21]^

Even with confirmation that reversion of **1** to starting materials can occur at higher temperatures, the observation of 0 % yield of **1** after warming and re‐cooling the reaction mixture was confounding; in a fully reversible sequence we would expect **1** to re‐form upon re‐cooling to −78 °C. A reasonable explanation for this is sequestration of one of the reactants due to undesired reactivity at higher temperatures. Deuterium quenching of the forward reaction showed formation of deuterated ketone **2‐d_1_**, suggesting that deprotonation of ketone **2** occurs during the reaction (Figure 1, Reaction 6). This product formed by enolization of **2** was also observed for the reverse reaction at room temperature after quenching with D_2_O (Figure 1, Reaction 5). We questioned whether **3 a** could act not only as a nucleophile but as a base, leading to undesired enolization. However, deuterated quinoline **3‐d_1_** was not observed upon reaction of **3 a** with deuterated ketone **2‐d_2_** at −78 °C (Figure 1, Reaction 4). Therefore, we suspected that deprotonation of ketone **2** occurs primarily by reaction with excess secondary amine or lithium amide base present in the reaction mixture. Enolate formation of **2** prevents a fully reversible sequence because the lithium enolate of **2** will not react with **3 a** to reform **1**.

One further undesired reaction of **2** that we identified is formation of product **7**. This undesired product was observed by HPLC‐MS, and an authentic sample was prepared to confirm the identity of this species (see Supporting Information). Presumably, this product forms by Michael addition of the secondary amine or amide to elimination product **6**. An important consequence of the formation of **7** could be formation of undesired derivatives of **1** with alternative amine functionalities. However, such products were not observed.

In summary, these mechanistic experiments in combination with our early optimization efforts led us to conclude that the reaction of LDA with **3** is unselective and low yielding and furthermore, that the reversibility of step 2 is particularly problematic at higher temperatures. A recent patent reported the use of lithium pyrrolidide as base to minimize formation of impurity **5**, but with a reduced yield of **1 a** (14 %).^[8]^ We assayed a mixture of lithium pyrrolidide with **3** by ^1^H NMR analysis to determine if altering the nature of the lithium amide base in step 1 could improve conversion of **3** and selectivity for formation of **3 a**. We observed 91 % formation of **3 a**, relative to 22 % with LDA under the same conditions (Figure 1, Reaction 3 and Scheme S11). This observation suggested that a stronger and less hindered base could provide an opportunity for drastic improvements to the synthesis of bedaquiline **1**. These mechanistic observations allowed us to further optimize for the synthesis of **1** with improved knowledge of the critical variables influencing its reproducible formation, particularly temperature control and the nature of the lithium amide base.

### Optimizing for Maximum Yield of Racemic Bedaquiline

**Figure 2:**
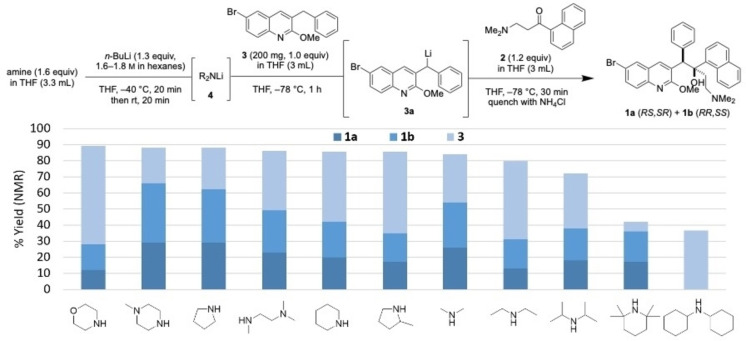
Evaluation of lithium amide bases in the lithiation/1,2‐addition sequence.

The mechanistic investigations described above suggest that altering the nature of the lithium amide base could lead to improved yield of key intermediate **3 a**. To investigate whether this observation represented a general trend, we evaluated a series of secondary amine bases which are used in combination with *n‐*BuLi to generate lithium amides with varying steric bulk and solubility profiles (Figure 2).[^22^, ^23^, ^24^, ^25^, ^26^, ^27^, ^28^, ^29^] We observed a compelling trend: less sterically hindered bases produced fewer undesired products and likewise, higher recovery of **3** and higher yield of **1**. Bases with high steric bulk such as dicyclohexylamine and 2,2,6,6‐tetramethylpiperidine gave lower overall yields with evidence of debromination, presumably through lithium‐halogen exchange with **3**. α‐Branched secondary amine bases such as diisopropylamine and 2‐methylpyrrolidine generally gave lower yields of 1,2‐addition product and lower mass balance of **3** relative to cyclic amines such as piperidine, pyrrolidine, and *N*‐methylpiperazine, which have lower hindrance and higher basicity.

In this assay, we formed the lithium amide at −40 °C, followed by warming to room temperature and re‐cooling to −78 °C prior to addition of **3**. The rationale for this temperature variation is based upon precedent which reports an influence of temperature on lithium aggregate formation.[^9^, ^16^] With lithium morpholide, the lithium amide solution turned brown in color upon warming to room temperature, and low yields were obtained in the subsequent 1,2‐addition, suggesting instability of lithium morpholide at higher temperatures. In subsequent assays, formation of the lithium amide at 0 °C for a shorter time and directly cooling to −78 °C led to improved yield (see below).

**Figure 3:**
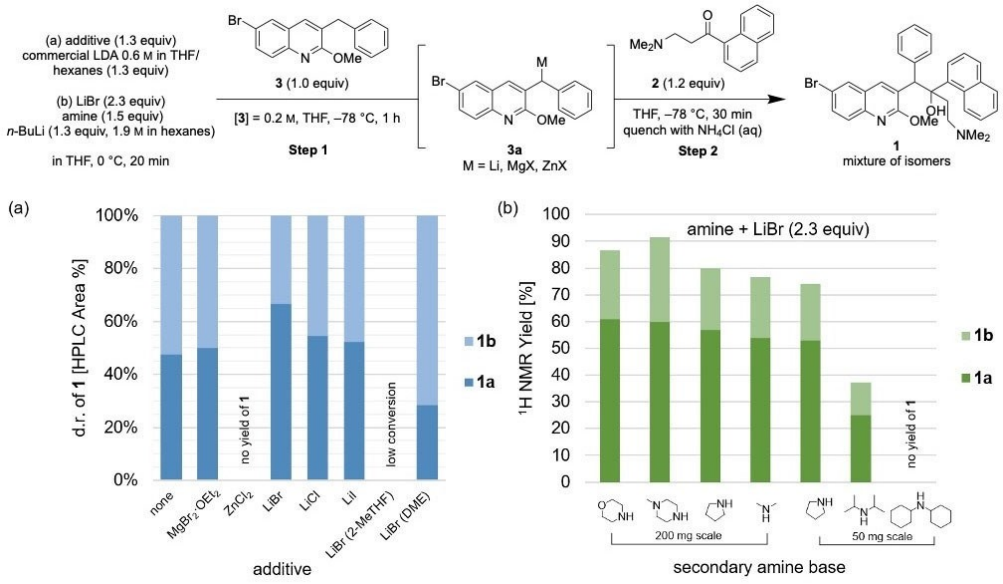
(a) Investigation of the influence of salt additives on d.r. of the 1,2‐addition reaction using commercial LDA solution as base. Diastereomer percentage composition determined by HPLC. (b) Assay of LiBr additive on d.r. and yield of 1,2‐addition with different lithium amide bases. Yield determined by ^1^H NMR spectroscopy using 1,4‐bis(trimethylsilyl)benzene as internal standard.

With knowledge that cyclic and less hindered lithium amide bases improve the yield of **1** and limit formation of undesired products, we sought to increase the diastereoselectivity of the 1,2‐addition to maximize formation of **1 a**. Salt additives are known to affect yield and diastereoselectivity in lithiation reactions by influencing the geometry, equilibrium, or rate of assembly or dissociation of lithium aggregates.[^30^, ^31^, ^32^, ^33^] Adding MgBr_2_⋅OEt_2_ resulted in no improvement of the diastereoselectivity (Figure 3a). When ZnCl_2_ was added, no conversion of **3** was observed, which was attributed to the formation of the less nucleophilic Zn‐organyl through Li−Zn exchange (Figure 3a). Cerium trichloride was evaluated as an additive based on precedent for promoting 1,2‐addition of enolizable ketones but the yield of **1 a** was not improved,^[34]^ which aligns with our earlier observation that **3 a** acts only as a nucleophile and not as a base towards ketone **2**.

A significant enhancement in diastereoselectivity was instead observed for the addition of LiBr (Figure 3a), reversing the d.r. of the reaction from 0.91 : 1.0 (**1 a** : **1 b**) to as high as 2.0 : 1.0, now favouring the desired *RS,SR*‐isomer. Similar enhancement was observed when LiBr was premixed with ketone **2** and added in step 2 (Supporting Information, Table S10). This observation could suggest that LiBr influences the d.r. by chelating the β‐amino ketone **2** and thereby impacting the approach of nucleophile **3 a**. In order to examine the effect of the counterion, LiCl and LiI were tested next, but no significant improvement in diastereoselectivity was observed (Figure 3a), possibly due to their lower solubility in THF. 2‐MeTHF and 1,2‐dimethoxyethane (DME) were investigated as alternative solvents; however, THF continued to demonstrate the highest yield and diastereoselectivity.

The stoichiometry of LiBr relative to quinoline **3** was assayed and it was determined that the use of 2.3 equivalents of LiBr was optimal when LDA was freshly prepared from *i‐*Pr_2_NH and *n‐*BuLi (Supporting Information, Table S8). However, when a commercial solution of LDA was used, only 1.3 equivalents of LiBr was required and further increases in stoichiometry did not have a beneficial effect. This difference is likely due to batch‐to‐batch variations in salt content of *n‐*BuLi and commercial LDA solutions, which is known to have important implications for the rate of lithiation reactions.[^16^, ^17^, ^18^]

LiBr used in combination with more basic, less sterically hindered lithium amides drastically improved the yield and diastereoselectivity of the 1,2‐addition reaction. The yield of **1** increased to as high as 92 % (*N*‐methylpiperazine) and d.r. improved to as high as 2.5 : 1.0 (**1 a** : **1 b**), more than doubling the yield of the desired *RS,SR*‐isomer (**1 a**) as compared to LDA (60 %, *N*‐methylpiperazine vs. 25 %, *i‐*Pr_2_NH). Across the series of bases, the same trend was observed as in the absence of salt additive; with diisopropylamine, low yield of **1** (37 %) was obtained, and with the bulky dicyclohexylamine, no product was formed (Figure 3b).

With optimal base and additive choices in hand, we investigated whether enhanced time and temperature control in continuous flow would improve the yield of **1 a**.^[35]^ We constructed a plug flow reactor to telescope the reaction and quench steps (Table 2). Using this setup, a comparable yield of **1** was achieved in a total residence time of 18.3 minutes, albeit at significantly decreased d.r. (Entry 1).

**Table 2:** Evaluation of a continuous flow process for synthesis of **1**.

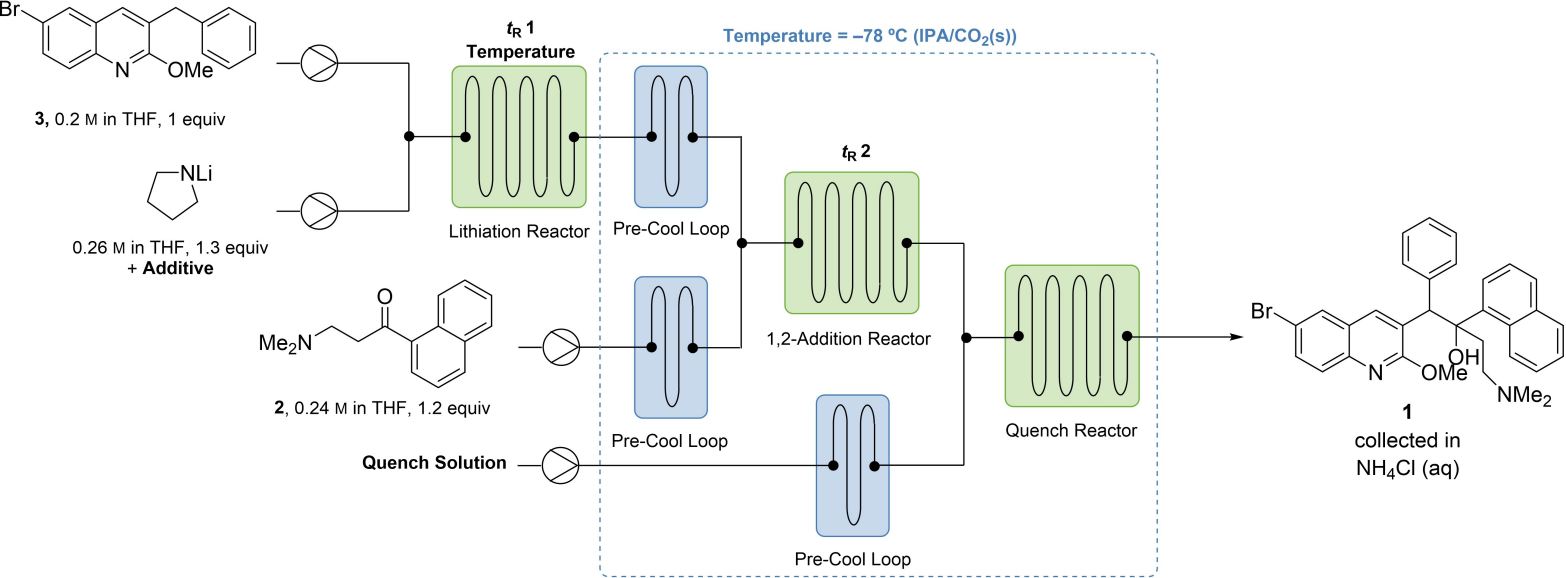							
Entry	Additive	Lithiation Temp. [°C]	*t*_R_ 1 [min]	*t*_R_ 2 [min]	Quench	AY **1 a** ^[a]^	AY **1** ^[a]^ (d.r.)^[b]^
1	None	−78	10	8.3	0.35 m AcOH in THF	30	74 (0.68 : 1.0)
2	LiBr^[d]^	−78	10	8.3	0.35 m AcOH in THF	–^[c]^	–^[c]^
3	None	22	2.5	8.3	0.35 m AcOH in THF	22	73 (0.43 : 1.0)
4	LiBr^[d]^, Et_3_N ⋅ HCl^[e]^	22	2.5	8.3	MeOH	42	72 (1.4 1.0)
5	LiBr^[d]^, Et_3_N ⋅ HCl^[e]^	22	1	5	MeOH	44	78 (1.3 1.0)
6	LiBr^[d]^	0	1	5	MeOH	42	73 (1.4 1.0)
7	LiBr^[d]^	0	0.4	2	MeOH	33	62 (1.1 1.0)

See Supporting Information Table S11 for additional details. [a] Determined by ^1^H NMR analysis with benzyl benzoate as internal standard. [b] AY **1 a** : AY **1 b**. [c] No data; failure due to clogging at quench. [d] 2.3 equiv. [e] 0.02 equiv.

We sought to investigate whether the LiBr additive could also improve d.r. in continuous flow. Formation of solids prevented the use of acetic acid as a quenching agent when LiBr was added (Table 2, Entry 2), while quenching with methanol prevented precipitation and enabled use of the salt additive in flow. LiBr did improve the d.r. of the reaction in flow (Entries 3–4), but the flow reaction remained less selective for the desired diastereomer **1 a** than the batch reaction. A LiCl salt additive (Et_3_N ⋅ HCl) was also explored due to precedent from Gupta et al. for rate enhancement in lithium amide deprotonation reactions,^[16]^ but the additive did not demonstrably affect the reaction (Entries 4–6).

In flow, the lithiation could be performed in as little as one minute at room temperature or 0 °C with rapid cooling before the 1,2‐addition at −78 °C (Entries 4–7); this observation highlights an advantage of flow, as a significant mid‐process temperature change is more feasible due to the smaller volumes. Further decrease in residence times led to decreased yield (Entry 7). Ultimately, while comparable yield of **1 a** was achieved using a plug flow reactor (Entry 5), the results did not improve upon our optimized batch conditions due to the lower diastereoselectivity. While shorter reaction times and higher deprotonation temperatures were also possible in batch (Supporting Information, Table S10), higher mass balance of **3** was observed at low temperatures (Supporting Information, Figure S1), and thus we focused our continued efforts on demonstrating a batch protocol amenable to larger scale processing that did not include the mid‐process cooling.

### Synthesis and Isolation of Racemic Bedaquiline

We translated our optimized batch reaction conditions to a 1 g scale reaction using the most promising amine bases: pyrrolidine, *N*‐methylpiperazine and morpholine (Table 3, Entries 1–3). The crude reaction mixture consisted of a mixture of isomers of **1** and unreacted starting materials **2** and **3**. For these larger scale reactions, we sought to develop conditions for separation of the desired diastereomer **1 a** from the other components of the reaction mixture by crystallization. Recrystallization from toluene resulted in selective crystallization of the undesired diastereomer **1 b**. The desired diastereomer **1 a** was isolated after recrystallization of the mother liquor from EtOH, resulting in isolation of **1 a** in up to 61 % yield and >99 % purity (1 g scale). A similar procedure was performed on 5 g and 10 g scale (Entries 4–5), leading to similar yield of isolated product **1 a**. Although the purity of **1 a** was lower (88–90 %) in the larger scale runs due to retention of **1 b**, we anticipate that application of the current industrial purification techniques including seeding can afford the desired diastereomer **1 a** in acceptable purity.

**Table 3:** Lithiation/1,2‐addition for synthesis of **1** on up to 10 g scale.

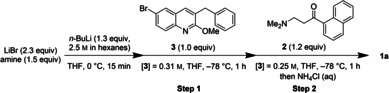					
Entry	Base	Scale [g of **3**]	Assay d.r.^[a]^	Yield **1 a** [%]^[b]^	Yield **1** [%]^[b]^
1	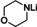	1	2.4 1.0	61	88
2	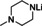	1	1.8 1.0	60	97
3	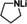	1	2.4 1.0	56	86
4^[c]^	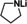	5	2.0 1.0	56	82
5^[c]^	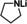	10	2.1 1.0	60	79

Yield: yield of isolated product. AY=assay yield [a] AY **1 a** : AY **1 b**, determined by ^1^H NMR spectroscopy of crude reaction mixture. [b] Yield of **1 a** are corrected for purity as determined by ^1^H NMR spectroscopy, while yield of **1 b** are uncorrected for purity. [c] *n*‐BuLi 1.4 m. Final [**3**]=0.30 m, lithium amide formation: 20–25 min, step 2: 30 min. Further details in Supporting Information.

## Conclusion

In summary, we have developed an improved process for the synthesis of racemic bedaquiline **1 a** by a higher yielding and more selective lithiation/1,2‐addition sequence enabled by use of LiBr as an additive and cyclic lithium amide bases. An initial focus on reproducibility of reported methods led to deeper understanding of the reaction mechanism, which guided optimization efforts and led to significant improvement over existing methods. We suggest important changes to the reaction sequence that we anticipate may be implemented on manufacturing scale and that will not require qualification of new intermediates or significant modifications to processing parameters. A future direction is further refinement of diastereoselectivity and/or enantioselectivity using chiral amine bases. This work is ongoing in our laboratories.

## Conflict of interest

The authors declare no conflict of interest.

1

## Supporting information

As a service to our authors and readers, this journal provides supporting information supplied by the authors. Such materials are peer reviewed and may be re‐organized for online delivery, but are not copy‐edited or typeset. Technical support issues arising from supporting information (other than missing files) should be addressed to the authors.

Supporting Information

## Data Availability

The data that support the findings of this study are available in the supplementary material of this article.
