# Supplementary material for: Diastereoselectivity is in the Details: Minor Changes Yield Major Improvements to the Synthesis of Bedaquiline
Source: Chemistry. 2022 Jul 7;28(47):e202201311. doi: 10.1002/chem.202201311 (PMC9545417; doi:10.1002/chem.202201311)
Supplement: Supplementary file 1 — Supporting Information [file CHEM-28-0-s001.pdf]

Chemistry–A European Journal

Supporting Information

Diastereoselectivity is in the Details: Minor Changes Yield Major Improvements to the Synthesis of Bedaquiline

Sarah Jane Mear, Tobias Lucas, Grace P. Ahlqvist, Juliana M. S. Robey, Jule-Philipp Dietz, Pankaj V. Khairnar, Sanjay Maity, Corshai L. Williams, David R. Snead, Ryan C. Nelson,* Till Opatz,* and Timothy F. Jamison*

SUPPORTING INFORMATION

GENERAL INFORMATION 3

Materials and Methods 3

Instrumentation and Analysis 3

Continuous Flow Apparatus 3

Laboratory Safety Statement 3

RELEVANT PRECEDENT FOR SYNTHESIS OF BEDAQUILINE 4

SYNTHESIS OF STARTING MATERIALS 5

Liberation of 3-(dimethylamino)-1-(naphthalen-1-yl)propan-1-one (2) from salt S1 5

Synthesis of 3-benzyl-6-bromo-2-methoxyquinoline (3) 5

***N*-(4-Bromophenyl)-3-phenylpropanamide (S3)** 6

3-Benzyl-6-bromo-2-chloroquinoline (S4) 6

3-Benzyl-6-bromo-2-methoxyquinoline (3) 6

SYNTHESIS OF COMPOUNDS FOR MECHANISTIC INVESTIGATION 7

3-(Dimethylamino)-1-(naphthalen-1-yl)propan-1-one-2,2-d$_2$ (2-d$_2$) 7

6-Bromo-2-methoxy-3-(phenylmethyl-d)quinoline (3-d$_1$, obtained as mixture with 3) 7

3-Benzyl-2-methoxyquinoline (5) 7

1-(Naphthalen-1-yl)-3-(pyrrolidin-1-yl)propan-1-one (7) 7

ESTABLISHING A REPRODUCIBLE BASELINE PROCEDURE FOR OPTIMIZATION 8

Temperature Sensitivity: Altering Quenching Methods 8

Stability of Lithiated Quinoline 3a Under Different Conditions 9

Influence of Step 2 (1,2-Addition) Temperature on Yield of 1 10

Unified Procedure: Baseline for Reaction Optimization Across Research Sites 11

MECHANISTIC INVESTIGATIONS 12

Deuterium Quenching of Forward Reaction 12

Control Experiment for Deuterium Quenching Assays: Background Incorporation of Deuterium into Ketone 2 by Enolization 13

Assay to Determine Whether 3a Acts as a Base: Reaction of 3a with 2-d$_2$ 14

Reverse Reaction at −78 °C and Room Temperature Using LDA 15

^{1}H NMR ASSAYS FOR FORMATION OF 3A 16

Characterization of 3a (^{1}H NMR, THF-d_8) at −78 °C 16

Characterization of 3a (^{1}H NMR, THF-d_8) at −78 °C 17

Variable Temperature ^{1}H NMR Experiment for Reaction Mixture Containing 3a 19

^{1}H NMR Assay for 3a Formation from Lithium Pyrrolidide and 3 at Room Temperature 20

REACTION OPTIMIZATION 21

Changing the Identity of the Secondary Amine Base 21

Assaying Influence of Salt Additives on Diastereoselectivity 22

General Procedure for Reaction Screening in Continuous Flow 26

SYNTHESIS OF (*RS,SR*)-1-(6-BROMO-2-METHOXYQUINOLIN-3-YL)-4-(DIMETHYLAMINO)-2-(NAPHTHALEN-1-YL)-1-PHENYLBUTAN-2-OL (1A) 28

^{1}H AND ^{13}C NMR SPECTRA 30

REFERENCES 45

APPENDIX: SYNTHESIS OF S1 AND 3 BY WUXI APPTECH 46

Synthesis of 3-(dimethylamino)-1-(naphthalen-1-yl)propan-1-one hydrochloride (S1) by WuXi AppTech: 47

Synthesis of 3-benzyl-6-bromo-2-methoxyquinoline (3) by WuXi AppTech: 48

^{1}H NMR Spectra for WuXi AppTech Products and Intermediates 49

General Information

Materials and Methods

Chemicals were obtained from commercial suppliers and were used without any further purification unless otherwise noted. Organometallic reagents were titrated according to a literature procedure[1] before first use and at least weekly thereafter. LiBr (anhydrous, various sources) was dried in a vacuum oven overnight at 100 ºC and stored in a desiccator before use. Anhydrous THF, 2-MeTHF and toluene were freshly distilled over sodium, purified using a solid-sorbent Solvent Dispensing System from Pure Process Technology, or taken from sealed bottles from Sigma-Aldrich (Darmstadt, Germany). For column chromatography, cyclohexane, hexanes and ethyl acetate were purchased in technical grade and distilled, or HPLC-grade anhydrous solvent was used. Deuterated solvents were purchased from Deutero GmbH (Kastellaun, Germany), Cambridge Isotope Labs (Cambridge, MA, USA) or Sigma Aldrich (Darnstadt, Germany). Dry MeOH was purchased from Acros Organics (Breda, Netherlands). All air or moisture sensitive reactions were performed under inert atmosphere (argon or nitrogen) in glassware that was dried using standard Schlenk techniques. Reaction temperatures referred to the temperature of the particular cooling or heating bath unless otherwise indicated. Chromatographic purification was performed using flash column chromatography of the indicated solvent system on silica gel (35–70 µm, Acros Organics) unless otherwise noted. Silica plates (TLC Silica 60 F_{254}, Merck, Darmstadt, Germany) were used for thin-layer chromatography. UV active compounds were detected using UV light (λ = 254 nm and λ = 365 nm).

Instrumentation and Analysis

All NMR spectra were recorded on the following spectrometers: Bruker Avance-III HD (^{1}H-NMR: 300 MHz, 400 MHz or 600 MHz, ^{13}C-NMR: 75.5 MHz), Bruker Avance-II (^{1}H-NMR: 400 MHz, ^{13}C-NMR: 100.6 MHz), Bruker Avance-III (^{1}H-NMR: 600 MHz, ^{13}C-NMR: 151 MHz). For ^{1}H-^{13}C HSQC and ^{1}H-^{13}C HMBC NMR experiments and for variable temperature NMR experiments, a three-channel Bruker Avance Neo spectrometer (500 MHz) equipped with a 5mm BBFO SmartProbe was used. Chemical shifts are referenced to residual solvent signals (chloroform-d_1: 7.26 ppm and 77.16 ppm, dimethylsulfoxide-d_6: 2.50 ppm and 39.52, and acetone-d_6: 2.05 ppm and 29.84 for ^{1}H-NMR and ^{13}C-NMR respectively) and reported in parts per million (ppm) relative to tetramethylsilane (^{1}H, ^{13}C). Infrared spectra were recorded on a spectrometer (Bruker Tensor 27, Bruker, Ettlingen, Germany) equipped with a diamond ATR unit. Electron spray ionization (ESI) mass spectra were recorded on a 1200-series HPLC-system or a 1260-series Infinity II HPLC-system (Agilent, Santa Clara, CA, USA) with binary pump and integrated diode array detector coupled to a LC/MSD-Trap-XTC-mass spectrometer (Agilent) or a LC/MSD Infinitylab LC/MSD (G6125B LC/MSD). High resolution mass spectrometry (HRMS) was performed using a JEOL JMS T100LC Accu-TOF mass spectrometer controlled by Mass Center software version 1.3.4 m (JEOL Inc., Tokyo, Japan). Melting points were determined by using a Krüss-Optronic (Hamburg, Germany) KSP 1 N digital melting point meter.

Analytical HPLC was performed using an Agilent 1260 Infinity system with a binary pump, a diode array detector, and an LC/MSD InfinityLab LC/MSD (G6125B LC/MSD) mass spectrometer. A Nucleodur C18 HTec column (1.8 µm, 4.6 mm × 30 mm, 40 °C with gradient elution (acetonitrile/water (+0.1% formic acid)) and a flow rate of 1.0 mL/min was used.

Continuous Flow Apparatus

High purity PFA tubing, PEEK mixers and unions, and Super Flangeless nuts and ferrules were purchased from IDEX Scientific (Oak Harbor, WA, USA). Syringe pumps were Harvard (Holliston, MA, USA) PhD Ultra syringe pumps with 8 mL or 20 mL stainless steel Harvard syringes or Syrris Asia (Royston, UK) pumps equipped with 250–500 µL (green) syringes. Solutions for flow reactions were prepared under an argon atmosphere using oven-dried volumetric glassware.

Laboratory Safety Statement

CAUTION: Commercial solutions of reagents such as *n*-butyllithium and lithium diisopropylamide are highly reactive and can be pyrophoric depending on concentrations. Use proper techniques for handling pyrophoric and water-reactive materials and ensure all reagents are fully quenched before work-up. In addition, chemical structures described herein may display bioactive properties. Handle with care.

Relevant Precedent for Synthesis of Bedaquiline

Table S1. Additional examples of synthesis of **1** reported in process patent applications and improvements described herein.

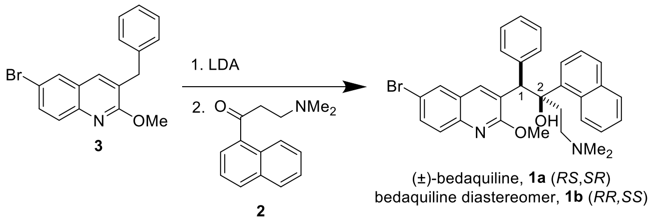

(±)-bedaquiline, **1a** (*RS*,*SR*)
bedaquiline diastereomer, **1b** (*RR*,*SS*)

Source	Base	Additive	Yield 1a [%][a]
Janssen 2004[2]	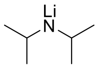	None	9%[b]
Janssen 2006[3]	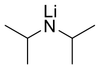	None	32%
Shanghai Institute of Pharmaceutical Industry 2017[4]	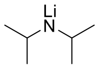	None	34%
Chinese Academy of Medical Sciences 2019[5]	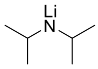	None	14%
Mylan 2020[6]	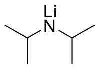	None	23%
Mylan 2020[6]	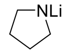	None	14%
**This work**	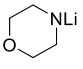 or 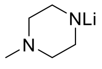 or 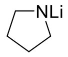	**LiBr**	**56–61%**

Yield: yield of isolated product [a] Adjusted for purity where reported [b] Isolated by column chromatography rather than recrystallization.

Synthesis of Starting Materials

Compounds **3** and **S1** (the hydrochloride salt of **2**) were obtained from WuXi AppTech (see Appendix) for use in reaction development described herein. The material prepared by WuXi AppTech was used across all labs for reaction optimization and scale-up. Synthesis of **3** was also explored in-house to validate and improve upon reported procedures and methods are described herein.

Liberation of 3-(dimethylamino)-1-(naphthalen-1-yl)propan-1-one (2) from salt S1

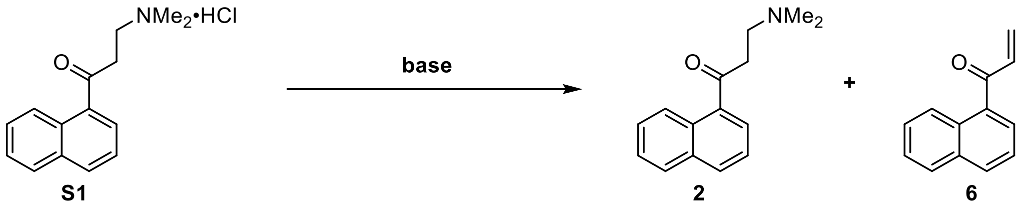

Scheme S1: Liberation of 3-(dimethylamino)-1-(naphthalen-1-yl)propan-1-one (**2**).

The free amine **2** was prepared by modification of a reported procedure.[3] A 25-mL round-bottom flask was charged with **S1** (502 mg, 1.90 mmol) and DI water (1.5 mL) was added. The mixture was stirred for 5 min until dissolution was observed. An aqueous solution of 25 wt% sodium hydroxide (2 mL) was added, and stirring was continued for 10 min. Additional DI water (4 mL) was added. Aggregation of water-insoluble **2** was observed. CH_2Cl_2 (6 mL) was added and stirring was continued for 5 min. The biphasic mixture was transferred to a separatory funnel. The layers were separated, and the aqueous layer was extracted with CH_2Cl_2 (5 mL). The organic layers were combined and washed with water, then dried over $MgSO_4$, filtered, and concentrated under reduced pressure. The title compound was isolated as a yellow oil (393 mg, 1.73 mmol, 91%). The ^{1}H NMR spectrum ($CDCl_3$) agreed with reported spectra.[3]

Notes. As the free amine **2** is prone to undergo elimination to enone **6**, it has to be freshly prepared from its hydrochloride salt **S1** (Scheme **S1**). Different approaches for preparing **2** were examined. When salt **S1** is dissolved in water and the amine **2** is liberated by adding $NaHCO_3$ or NaOH solution, significant amounts of elimination byproduct **6** are observed by LC-MS if extended reaction time is used, or if elevated temperatures (≥ 25 °C) are used during rotary evaporation. Alternatively, the salt **S1** was suspended in CH_2Cl_2 and washed with saturated $NaHCO_3$ solution using a separatory funnel to obtain **2** after solvent removal.

Synthesis of 3-benzyl-6-bromo-2-methoxyquinoline (3)

In summary, quinoline **3** was prepared according to a modified method by Kong et al. (Scheme **S2**) starting from readily available 4-bromoaniline, which was *N*-acylated to amide **S3** in 79% yield using 3-phenylpropanoyl chloride (**S2**).[7] In the next step, a Vilsmeier-Haack reaction and subsequent condensation furnished quinoline **S4** in 72% yield. After nucleophilic substitution with sodium methoxide and recrystallization, the desired product **3** was obtained in 96% yield. All steps were performed on a 5–10 g scale avoiding any chromatographic purification step. After recrystallization from MeOH, **3** had a purity of 99% (HPLC, 254 nm).

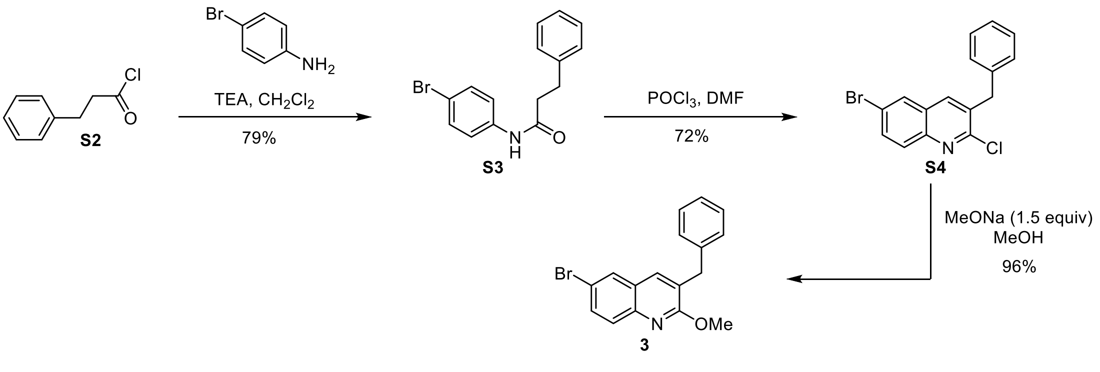

Scheme S2: Synthesis of 3-benzyl-6-bromo-2-methoxyquinoline (**3**).

N-(4-Bromophenyl)-3-phenylpropanamide (S3)

4-Bromaniline (10.6 g, 61.7 mmol, 1.1 equiv) was dissolved in CH_2Cl_2 (50 mL) and triethylamine (9 mL). The solution was cooled to 0 °C and 3-phenylpropanoyl chloride (**S2**,10.0 g, 59.3 mmol, 1.0 equiv) was added dropwise and after complete addition, the reaction mixture was stirred overnight at room temperature. The reaction was quenched with water (200 mL) and brought to pH 2 with 10% aqueous HCl solution. The slurry was stirred for 1 h with ice-bath cooling and the colorless solid was filtered and washed with diethyl ether. After drying in vacuo, a colorless solid was obtained. The analytical data are consistent with those reported in the literature.[7,8]

Yield: 14.3 g (47.1 mmol, 79%), colorless solid.
R$_f$: 0.5 (^{c}Hex/EtOAc 2:1)
Melting point: 148.8–150.4 °C.
^{1}H-NMR, COSY (300 MHz, $CDCl_3$): δ/ppm = 7.43–7.36 (m, 2H, *m*-H, Ph-Br), 7.35–7.27 (m, 4H, *m*-H, Ph and *o*-C, Ph-Br), 7.55–7.19 (m, 3H, *o*-H, and *p*-C, Ph) 7.12 (s, 1H, NH), 3.04 (t, *J* = 7.6 Hz, 2H, Ph-CH_2), 2.65 (t, *J* = 7.6 Hz, 2H, O=C-CH_2).
^{13}C-NMR, HSQC, HMBC (75 MHz, $CDCl_3$): δ/ppm = 170.4 (C=O), 140.5 (*ipso*-C, Ph), 136.8 (*ipso*-C, Ph-Br), 131.9 (*m*-C, Ph-Br), 128.7 (*m*-C, Ph), 128.4 (*o*-C, Ph), 126.5 (*p*-C, Ph), 121.4 (*o*-C, Ph-Br), 116.9 (*p*-C, Ph-Br), 39.5 (**C**-C=O), 31.5 (**C**-C-Ph).
IR: 3675, 3294, 2973, 2901, 2605, 2498, 1658, 1590, 1522, 1073, 817.
MS (ESI): *m/z* (%) = 304.0 (100), 306.0 (96) $[M+H]^+$.

3-Benzyl-6-bromo-2-chloroquinoline (S4)

A flask was charged with DMF (14 mL, 180 mmol, 4.0 equiv) and it was cooled to 0 °C. Under stirring, $POCl_3$ (32.9 g, 360 mmol, 8.0 equiv) was slowly added dropwise. After complete addition, the mixture was stirred for 1 h at room temperature followed by addition of a solution of **S3** (13.7 g, 45.2 mmol, 1.0 equiv) in MeCN (22 mL). The mixture was stirred overnight at 80 °C. After completion, MeCN was partially removed and the mixture was poured in ice-water (200 mL) and stirred until **S4** precipitates. The colorless needles are separated by filtration, washed with 0 °C MeOH and dried in vacuo. The analytical data are consistent with those reported in the literature.[7,9]

Yield: 10.7 g (32.2 mmol, 72%), slightly yellow solid.
R$_f$: 0.58 (^{c}Hex/EtOAc 4:1)
Melting point: 108.7–109.1 °C.
^{1}H-NMR, COSY (300 MHz, $CDCl_3$): δ/ppm = 7.91–7.85 (m, 2H, 5-H and 8-H), 7.75 (dd, *J* = 9.0 Hz, 2.1 Hz 1H, 7-H), 7.68 (s, 1H, 4-H) 7.42–7.21 (m, 5H, Ph), 4.24 (s, 2H, CH_2).
^{13}C-NMR, HSQC, HMBC (75 MHz, $CDCl_3$): δ/ppm = 152.1 (C-1), 145.2 (C-8a), 137.7 (*ipso*-C, Ph), 137.1 (C-4), 134.6 (C-3), 133.5 (C-7), 130.0 (C-8), 129.4 (*o*-C, Ph, C-5), 129.0 (*m*-C, Ph), 128.67 (C-4a), 127.1 (*p*-C, Ph), 121.0 (C-6), 39.2 (CH_2).
IR: 3674, 2987, 2972, 2901. 1452, 1406, 1394, 1382, 1251, 1230, 1075, 1066.
MS (ESI): *m/z* (%) = 332.0 (77), 334.0 (100) $[M+H]^+$.

3-Benzyl-6-bromo-2-methoxyquinoline (3)

3-Benzyl-6-bromo-2-chloroquinoline (**S4**) (6.48 g, 19.5 mmol, 1.0 equiv) was dissolved in MeOH (50 mL) and under stirring a 30% sodium methoxide solution in MeOH (3.70 mL, 29.2 mmol, 1.5 equiv) was added. The mixture was refluxed overnight. Upon cooling to –20 °C, the precipitate was filtered off and washed with cold water. After recrystallization from MeOH, the desired product **3** was obtained as colorless needles. The analytical data are consistent with those reported in the literature.[7,9]

Yield: 6.13 g (18.7 mmol, 96%), colorless needles.
R$_f$: 0.44 (^{c}Hex/EtOAc 50:1)
Melting point: 84.7–85.1 °C.
^{1}H-NMR, COSY (300 MHz, $CDCl_3$): δ/ppm = 7.75 (d, *J* = 2.2 Hz, 1H, H-5), 7.70 (d, *J* = 8.9 Hz, 1H, H-8), 7.61 (dd, *J* = 8.9 Hz, 2.2 Hz, 1H, H-7), 7.49 (s, 1H, 4-H), 7.38–7.29 (m, 2H, *m*-H, Ph), 7.28–7.21 (m, 3H, *o*-H and *p*-H, Ph), 4.09 (s, 3H, OMe), 4.03 (s, 2H, CH_2).
^{13}C-NMR, HSQC, HMBC (75 MHz, $CDCl_3$): δ/ppm = 161.3 (C-1), 144.2 (C-8a), 139.0 (*ipso*-C, Ph), 136.0 (C-4), 132.0 (C-7), 129.4 (*o*-C, Ph), 129.2 (C-5), 128.7 (*m*-C, Ph), 128.6 (C-8), 127.2 (C-2), 126.8 (C-4a), 126.6 ((*p*-C, Ph), 117.2 (C-6), 53.9 (OMe), 36.2 (CH_2).
IR: 3674, 2987, 2901, 1622, 1599, 1567, 1492, 1461, 1399, 1252, 1064.
MS (ESI): *m/z* (%) = 328.0 (100), 330.0 (98) $[M+H]^+$.

Synthesis of Compounds for Mechanistic Investigation

3-(Dimethylamino)-1-(naphthalen-1-yl)propan-1-one-2,2-d$_2$ (2-d$_2$)

Compound **2-d$_2$** was synthesized for use in mechanistic experiments (*vide infra*).
Deuterium oxide (8 mL) was added to a vial containing **2** (1.74 g, 7.65 mmol). The reaction was vigorously stirred at room temperature for 4 days. The reaction was extracted with CH_2Cl_2 (3 x 10 mL) and washed with saturated aqueous sodium chloride solution. The organic phase was dried over Na_2SO_4 and the solvent was removed under reduced pressure and further dried under vacuum.
Yield: 1.57 g (6.89 mmol, 90%), yellow oil
^{1}H-NMR (600 MHz, $CDCl_3$): δ/ppm = 8.61 (d, *J* = 8.7 Hz, 1H), 7.97 (d, *J* = 8.2 Hz, 1H), 7.87 (ddd, *J* = 6.9, 5.1, 1.3 Hz, 2H), 7.59 (ddd, *J* = 8.5, 6.9, 1.3 Hz, 1H), 7.56–7.46 (m, 2H), 2.81 (s, 2H), 2.29 (s, 6H).
^{13}C-NMR (151 MHz, $CDCl_3$): δ/ppm = 136.1, 134.0, 132.6, 132.5, 130.2, 128.4, 127.9, 127.4, 126.5, 125.9, 124.4, 54.7, 45.5.
HRMS (DART): *m/z* calc'd for $C_{15}H_{16}D_2NO^+$: 230.1514 [*M*+H]$^+$; found: 230.1481.

6-Bromo-2-methoxy-3-(phenylmethyl-d)quinoline (3-d$_1$, obtained as mixture with 3)

Compound **3-d$_1$** was synthesized to confirm NMR assignments for mechanistic experiments (*vide infra*).
In an oven-dried vial containing anhydrous THF (2 mL) under N_2 atmosphere, freshly distilled *N*-methylpiperazine (88 µL, 0.79 mmol, 1.3 equiv) was added. The resulting solution was cooled to 0 ºC followed by slow addition of titrated *n*-BuLi (2.45 M in hexanes, 322 µL, 0.79 mmol, 1.3 equiv). After 20 min, a solution of **3** (200 mg, 0.61 mmol, 1.0 equiv) in anhydrous THF (2 mL) was added dropwise into the reaction mixture, which was then warmed to room temperature and stirred for additional 3 min. The reaction was quenched with TFA-d (139 µL, 1.8 mmol, 3 equiv). The solvent was removed and the crude material was purified by silica gel column chromatography (hexanes/EtOAc 9:1). The purified material contained a mixture of **3** and **3-d$_1$** in a ratio of approximately 4:1 by ^{1}H NMR analysis.
NMR: Spectra of the mixture were identical to that of **3** except for the appearance of the following resonances: ^{1}H NMR (600 MHz, $CDCl_3$): δ/ppm = 3.97 (s, -CDH-); ^{13}C{^{1}H} (151 MHz, $CDCl_3$): δ/ppm = 35.8 (-CDH-). DEPT-135 and ^{1}H-^{13}C HSQC further confirmed the assignment of these peaks (see Spectra section).

3-Benzyl-2-methoxyquinoline (5)

An authentic sample of compound **5** was synthesized to confirm its observation as a side product.
Quinoline **3** (200 mg, 0.61 mmol) was dissolved in 2 mL of dry THF under nitrogen atmosphere, and the vial containing this solution was cooled to –78 °C. Titrated *n*-BuLi was added dropwise (2.5 M solution in THF, 1.3 equiv), causing a reddish color to form. The reaction was stirred at –78 °C for 1 h, then quenched by slow addition of 3 mL of saturated aqueous NH_4Cl solution. Full consumption of quinoline **3** was observed by LC-MS and ^{13}C NMR. ^{1}H NMR analysis indicated that **5** was obtained in 95% assay yield. Column chromatography over silica gel (Hexanes/EtOAc, 8:2) resulted in 90% yield of isolated product (corrected for purity by ^{1}H NMR analysis).
Yield: 0.137 g (90%), white solid
^{1}H-NMR (600 MHz, $CDCl_3$): δ/ppm = 7.77 (d, *J* = 8.4 Hz, 1H), 7.56–7.46 (m, 3H), 7.29–7.22 (m, 3H), 7.21–7.14 (m, 3H), 4.03 (s, 3H), 3.98 (s, 2H).
^{13}C-NMR (151 MHz, acetone-d$_6$): δ/ppm = 205.2, 160.8, 145.5, 139.7, 137.1, 129.0, 128.7, 128.4, 127.1, 126.7, 126.2, 125.9, 125.6, 124.0, 52.9, 35.7.
The analytical data are consistent with those reported in the literature.[10]

1-(Naphthalen-1-yl)-3-(pyrrolidin-1-yl)propan-1-one (7)

An authentic sample of compound **7** was synthesized to confirm its observation as a side product.
Ketone **2** (300 mg, 1.32 mmol) was dissolved in 1 mL of CH_2Cl_2, then pyrrolidine (0.54 mL, 6.60 mmol, 5 equiv) was added to the solution. The reaction was stirred at room temperature for 12 h. Full consumption of ketone **2** was observed by TLC and ^{1}H NMR analysis. The crude reaction mixture was washed with DI water (2 x 5 mL), then the aqueous layer was extracted with CH_2Cl_2 (2 x 5 mL). The combined organic layers were dried with anhydrous Na_2SO_4 and solvent was removed under reduced pressure. The sample was purified by column chromatography in silica gel (DCM/MeOH, 9.5:0.5). Ketone **7** was obtained in 87% yield of isolated product (corrected for purity, ^{1}H NMR).
Yield: 0.291 g (87%), yellow oil
^{1}H-NMR (600 MHz, $CDCl_3$): δ/ppm = 8.57 (d, *J* = 8.6 Hz, 1H), 7.93 (d, *J* = 8.2 Hz, 1H), 7.87–7.79 (m, 2H), 7.58–7.42 (m, 3H), 3.28 (t, *J* = 7.4 Hz, 2H), 2.95 (t, *J* = 7.4 Hz, 2H), 2.59–2.48 (m, 4H), 1.80–1.69 (m, 4H).
^{13}C-NMR (151 MHz, $CDCl_3$): δ/ppm = 203.4, 136.0, 133.9, 132.5, 130.1, 128.4, 127.8, 127.3, 126.4, 125.8, 124.4, 54.1, 51.3, 41.7, 23.5.
HRMS (DART): *m/z* calc'd for $C_{17}H_{20}NO^+$: 254.1545 [*M*+H]$^+$; found: 254.1540.

Establishing a Reproducible Baseline Procedure for Optimization

Temperature Sensitivity: Altering Quenching Methods

Quinoline **3** (50 mg, 0.15 mmol, 1.0 equiv) was added to a dry 2-dram vial, followed by a dry stir bar. The vial was placed under high vacuum and dried for 2 h at room temperature. THF (500 µL) was added and the solution was cooled in an IPA/dry ice bath. LDA (130 µL of 1.5 M solution in THF/heptane/PhEt, 1.3 equiv) was added dropwise, at which point the reaction takes on a dark purple color. The mixture was stirred for 1 h. Ketone **2** (41 mg, 0.18 mmol, 1.2 equiv) was weighed into a dry 2-dram vial and placed under high vacuum for 30 min. The ketone was dissolved in THF (1 mL), then added dropwise to the stirring reaction mixture over 3 min. The resulting mixture was stirred for a further 30 min. The reaction mixture was quenched by dropwise addition of quench solution (25 wt% NH_4Cl or 1 M AcOH in THF, as indicated). After a yellow color was observed, the cooling bath was removed and the mixture was allowed to warm to room temperature. Note: on addition of NH_4Cl (aq) solution, freezing of the aqueous mixture occurs, resulting in a biphasic mixture. The mixture was stirred until a homogenous room temperature solution was obtained. Benzyl benzoate (28.5 µL, 0.150 mmol, 1 equiv) was added and the vial was shaken to dissolve. An aliquot of the organic layer was taken and the solvent was removed from the aliquot under reduced pressure. The residue was suspended in $CDCl_3$ and analyzed by ^{1}H NMR spectroscopy with a 30 s relaxation delay to ensure accurate quantification.

Table S2. Initial assessment of lithiation/1,2-addition reaction parameters indicating the importance of reagent addition and temperature control.

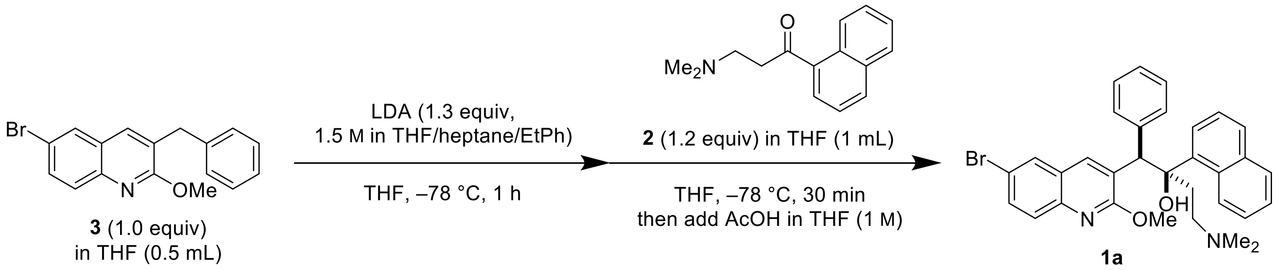

entry	deviation from scheme	AY [%][a] 1a	AY [%][a] 1 (d.r.)[b]
1	None (AcOH quench)	26	53 (1.0:1.0)
2	quench NH_4Cl (aq)	17	35 (0.9:1.0)
3	Add **3** to LDA	22	45 (1.0:1.0)
4	warm to rt before quench	0	0
5	$CeCl_3$ (1.0 equiv) + LDA, then add **3**	23	42 (1.2:1.0)

[a] Assay yield determined by ^{1}H NMR spectroscopy using benzyl benzoate as internal standard

[b] AY **1a** : AY **1b**

Stability of Lithiated Quinoline 3a Under Different Conditions

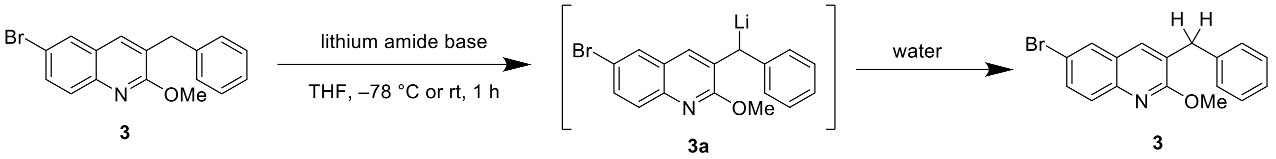

Scheme S3. Assay for stability of **3a** under different reaction conditions.

Experimental Procedure. Amine (^{i}Pr$_2$NH, pyrrolidine or *N*-methylpiperazine, 0.91 mmol, 1.5 equiv) was added to a vial containing anhydrous THF (2 mL) at 0 °C. Then, *n*-BuLi (2.5 M in THF, 0.79 mmol, 1.3 equiv) was added dropwise. The reaction mixture was kept at the same temperature over 20 min, then transferred to a bath at −78 °C or moved to room temperature. Quinoline **3** (200 mg, 0.61 mmol) was dissolved in anhydrous THF (2 mL) and then added dropwise to the vial containing the lithium amide base. The reaction mixture was stirred under nitrogen atmosphere for the indicated time (15−60 min) prior to quenching with water (3 mL). Triphenylmethane (0.61 mmol, 1.0 equiv) was added to the sample as the NMR internal standard. The organic phase was separated and dried with anhydrous Na_2SO_4. The solvent was removed under reduced pressure prior to ^{1}H NMR analysis. Each reaction was performed in duplicate and the results averaged. As shown in Figure S1, recovery of **3** is significantly higher at −78 °C and with cyclic amine bases as compared to diisopropylamine.

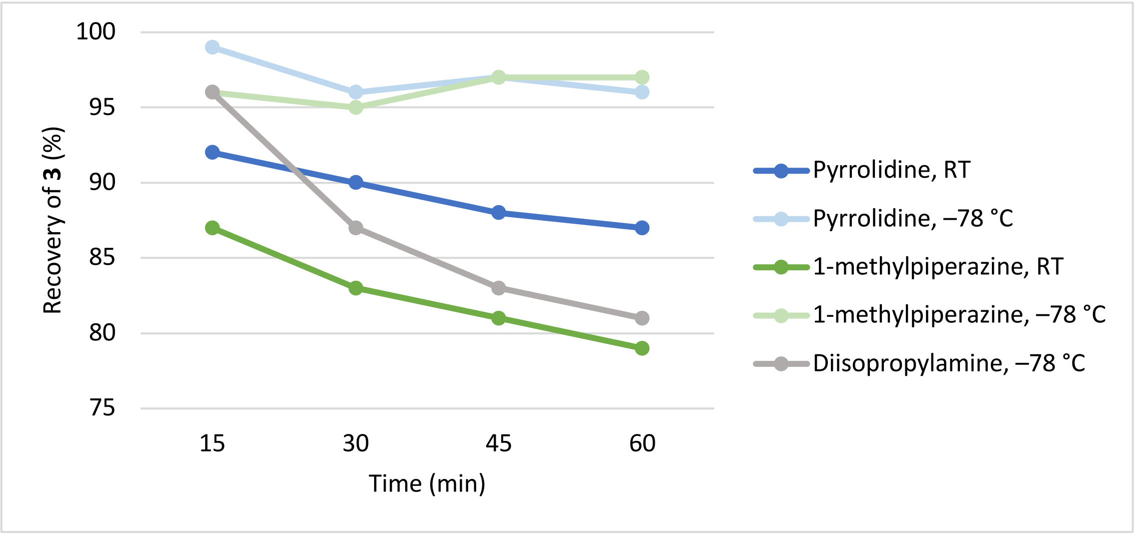

Figure S1. Stability of lithiated quinoline **3a** as determined by recovery of **3** after quench. Data points are an average of duplicate experiments.

Influence of Step 2 (1,2-Addition) Temperature on Yield of 1

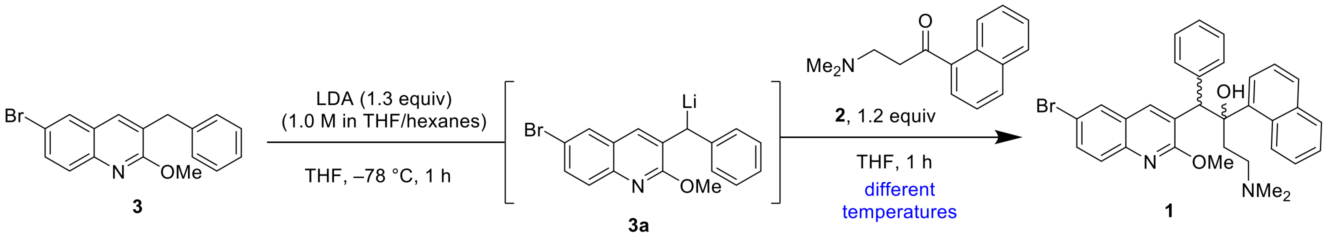

Scheme S4. Assay to determine the influence of temperature for Step 2 on yield of **1**.

Experimental Procedure. Quinoline **3** (150 mg, 0.46 mmol, 1.0 equiv) was dissolved in anhydrous THF (0.5 mL) and the reaction mixture was cooled to −78 °C. Commercial LDA solution (1 M in THF/hexanes, 0.60 mmol, 0.60 mL, 1.3 equiv) was added dropwise and reaction mixture was stirred under argon atmosphere. After a period of 1 h, a solution of ketone **2** (125 mg, 0.55 mmol, 1.2 equiv) in anhydrous THF (0.5 mL) was added dropwise to the vial containing lithiated quinoline **3a**. After addition of the ketone **2**, the reaction vial was transferred to a temperature-controlled bath (−78, −40 or −20 °C), and the reaction was stirred for additional 1 h. The resulting mixture was quenched with a saturated aqueous solution of NH_4Cl (1 mL). Mesitylene was added as the NMR internal standard (0.46 mmol, 1.0 equiv) prior to phase separation. The organic phase was dried with anhydrous Na_2SO_4 and concentrated prior to ^{1}H NMR analysis.

^{1}H NMR and visual analysis: At –78 °C (dry ice/acetone bath), 35% AY of **1** was obtained. At higher temperatures, no bedaquiline (**1**) was observed in the ^{1}H NMR spectrum of the crude reaction. A difference in color was observed depending on the temperature that the second step was performed. When the 1,2-addition was carried out at −78 °C, the purple color formed during lithiation step remained until the moment of the quench with aqueous solution of NH_4Cl. However, when the temperature was increased, the addition of ketone **2** (−40 or −20 °C) caused a rapid color change from purple to brown. The ^{1}H NMR spectroscopy and LC-MS analysis for the latter cases showed formation of some side products, among them the debrominated quinoline **5**, but most of the mass balance (80–90%) consisted of the starting materials **2** and **3**.

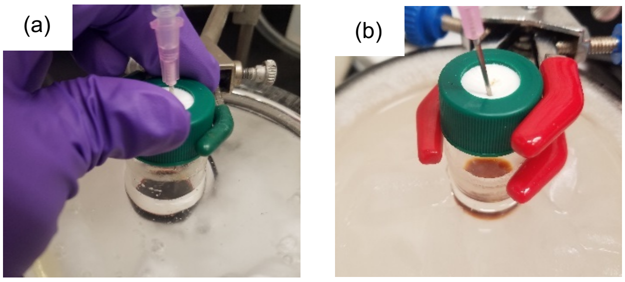

Figure S2. Color of the reaction mixture after ketone **2** addition: (a) Reaction at −78 °C, intense purple color during both steps; (b) Reaction at −40 °C, brown color developed during second step.

Unified Procedure: Baseline for Reaction Optimization Across Research Sites

The following list describes a general procedure which guided our three research sites (JGU Mainz, VCU, MIT) to align our methods for reaction optimization, mechanistic investigations, and other experimental efforts. This procedure is used unless otherwise noted.

1. Pre-dry three vials or flasks, either by flame-drying or by heating in a 140 ºC oven for a minimum of 1 h.
2. Charge quinoline **3** (200 mg, 1.0 equiv) into a flask, and put under high vacuum for 1 h. (FLASK A)
3. Charge additive (if necessary) into another flask, and put under high vacuum for 1 h. (FLASK B)
4. Charge ketone **2** (164 mg, 1.2 equiv) to a third flask, and put under high vacuum for at least 1 h. (FLASK C)
5. FLASK B. Charge flask with anhydrous THF (1.0 mL, 5 vol) and amine (1.5 equiv, dried over CaH_2 and distilled before use, or commercially redistilled). Cool to −78 °C in a bath of dry ice and acetone or isopropanol. Hold at temperature for 10 min.
6. FLASK B. Add *n*-BuLi (1.6 or 2.5 M in hexanes, titrated prior to use). Upon complete addition, allow reaction mixture to stir for 10 min.
7. FLASK A. Add anhydrous THF (2.0 mL, 10 vol) and mix to dissolve the quinoline **3**.
8. Transfer the contents of FLASK A into FLASK B dropwise over the course of 10 min. Stir for 1 h at −78 °C.
9. FLASK C. Add anhydrous THF (3.0 mL, 15 vol) to dissolve the ketone **2**.
10. Transfer the contents of FLASK C into FLASK B dropwise over the course of 15 min. Stir for 1 h at −78 °C.
11. Add saturated aqueous NH_4Cl (1 mL, approx. 5 equiv) to FLASK B very slowly (approximately one drop every 20 seconds). Remove FLASK B from cooling bath and allow to warm to room temperature when visibly quenched (reaction mixture turns from deep purple to yellow).
12. Separate the organic layers. Extract the aqueous with THF or CH_2Cl_2 (3 x 1 mL). Remove solvent under reduced pressure. (CRUDE). *Note: Later it was determined that choice of workup solvent influenced the yield of observed product due to solubility of **1**.*
13. Dissolve CRUDE in desired solvent. Add internal standard for ^1H NMR analysis and dissolve CRUDE in $CDCl_3$. Take ^1H NMR spectrum with 30 s relaxation delay.

Table S3. Results of “baseline procedure” by different operators at different institutions.

Entry	Operator, Institution	Scale of **3**	AY [%][a] **1a**	AY [%] **1** (d.r.)[b]
1	A, MIT	200 mg	20	41 (1.0 : 1.1)
2	B, JGU Mainz	50 mg	25	52 (1.0 : 1.1)
3	C, VCU	200 mg	19	46 (1.0 : 1.3)

[a] Assay yield determined by ^1H NMR analysis using internal standard
[0] AY **1a** : AY **1b**

Mechanistic Investigations

Deuterium Quenching of Forward Reaction

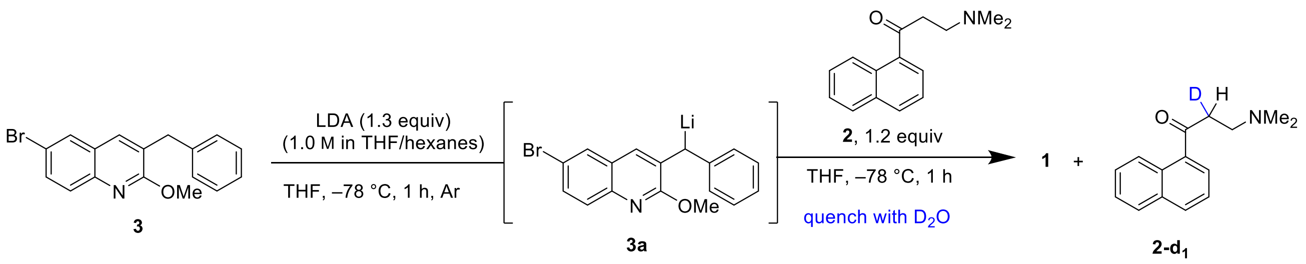

Scheme S5. Forward reaction for formation of bedaquiline **1** with deuterium oxide quenching.

Experimental Procedure. Quinoline **3** (150 mg, 0.46 mmol, 1.0 equiv) was dissolved in anhydrous THF (0.5 mL) and the reaction mixture was cooled to −78 °C (dry ice/acetone bath). Commercial LDA (1 M in THF/hexanes, 0.6 mmol, 0.6 mL, 1.3 equiv) was added dropwise and reaction was stirred under argon atmosphere. After a period of 1 h, a solution of ketone **2** (0.55 mmol, 1.2 equiv) in anhydrous THF (0.5 mL) was added dropwise to the vial containing the lithiated quinoline **3a**. The reaction mixture was stirred for additional 1 h at the same temperature, then quenched with deuterium oxide (2 mL). After phase separation, the organic layer was dried with anhydrous Na_2SO_4, and the solvent removed under reduced pressure prior to ^1H NMR analysis.

Notes. Analysis of the ^1H NMR spectrum of the crude reaction mixture, after quenching with deuterium oxide. No deuterium incorporation into quinoline **3** was observed. Approx. 45% of deuterium incorporation at the α-position of ketone **2** was observed.

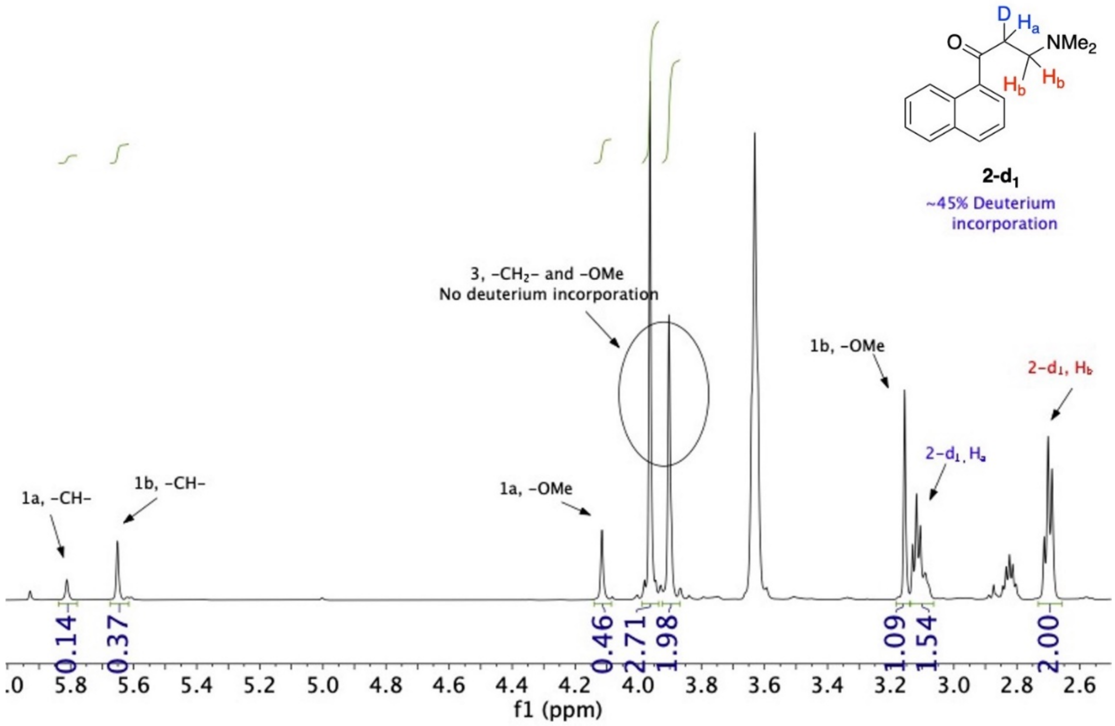

Figure S3. ^1H NMR spectrum of the crude reaction product after quenching with deuterium oxide.

Control Experiment for Deuterium Quenching Assays: Background Incorporation of Deuterium into Ketone 2 by Enolization

Experimental Procedure. A series of control experiments were performed to test for background H/D exchange between the ketone **2** and deuterium oxide. In these tests, ketone **2** (125 mg, 0.55 mmol) was dissolved in a THF-D_2O mixture (0.90 mL, 2 mL), and this reaction mixture was monitored over the course of 1 h by ^{1}H NMR spectroscopy.

Notes. These experiments showed that deuterium can be incorporated into the ketone **2** simply by reacting **2** with D_2O at room temperature; however, this exchange is slow relative to the workup and assay procedure used. As indicated in the table below, incorporation of 48% of deuterium into the ketone **2** was observed after stirring the solution during 1 h. In the bedaquiline synthesis, the quench process and sample preparation for NMR analysis take less than 15 min. However, due to the complex matrix in solution post-quench, background deuterium incorporation should be considered when interpreting results of this assay.

Table S4. Deuterium incorporation over time when ketone **2** is reacted with deuterium oxide.

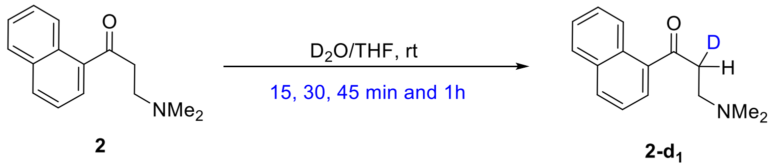

Reaction Time [min]	Deuterium Incorporation [%]
15	12
30	24
45	37
60	48

Assay to Determine Whether 3a Acts as a Base: Reaction of 3a with 2-d$_2$

Once enolization of **2** was suspected, we desired to know whether ketone **2** was reacting only with the lithium amide base or also with lithiated quinoline **3a**. To understand if intermediate **3a** acts both as base and nucleophile, we reacted **3a** with deuterated ketone **2-d$_2$**.

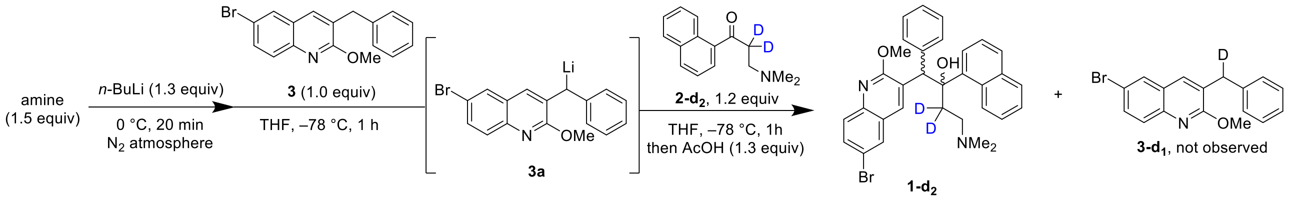

Scheme S6. Reaction of **3a** with **2-d$_2$** to assay whether **3a** acts as a base for undesired enolization of **2**.

Experimental Procedure. In an oven-dried vial containing anhydrous THF (2 mL), freshly distilled pyrrolidine or ^{i}Pr$_2$NH (0.91 mmol, 1.5 equiv) was added, and the resulting solution was cooled to 0 °C followed by slow addition of titrated *n*-BuLi (320 µL of 2.45 M in hexanes, 0.79 mmol, 1.3 equiv). After 20 min, the reaction mixture was cooled to −78 °C, and a solution of quinoline **3** (200 mg, 0.61 mmol, 1.0 equiv) in anhydrous THF (2 mL) was added dropwise and stirred under nitrogen atmosphere. After a period of 1 h, a solution of ketone **2-d$_2$** (170 mg, 0.73 mmol, 1.2 equiv) in anhydrous THF (2 mL) was slowly added to the vial containing **3a**. The reaction mixture was stirred for additional 1 h at the same temperature. The resulting mixture was quenched with AcOH (45 µL, 0.79 mmol, 1.3 equiv), and the solvent removed from the sample prior to ^{1}H NMR analysis. **3-d$_1$** was not observed using pyrrolidine or ^{i}Pr$_2$NH.

Notes. The ^{1}H NMR spectrum of the crude reaction using LDA was complex, causing difficulties in the quantification of deuterium incorporation. Isolation of **3** by column chromatography (hexanes:EtOAc, 9:1) and further analysis by LC-MS indicated that the main side product contaminating the sample was the debrominated quinoline **5**. ^{13}C NMR lacked C-D coupling at 36 ppm that would be expected in **3-d$_1$**.

Reverse Reaction at −78 °C and Room Temperature Using LDA

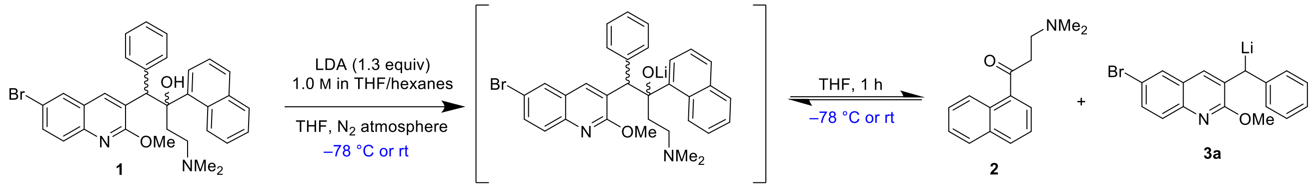

Scheme S7. Formation of **2** and **3a** by treatment of bedaquiline with LDA at different temperatures.

Experimental Procedure. Bedaquiline **1** (d.r. 1:0.4, **1a**:**1b**, 69% purity, 63.5 mg, 0.23 mmol) was dissolved in anhydrous THF (10 mL) with mild heating. This solution was held at room temperature or transferred to a −78 °C bath (dry ice/acetone), as indicated. Commercial LDA solution (300 μL of 1.0 M in THF/hexanes, 0.30 mmol, 1.3 equiv) was added dropwise and the mixture was stirred for 1 h under nitrogen atmosphere. The reaction mixture was quenched with saturated aqueous NH_4Cl solution (10 mL). Extraction was performed with CH_2Cl_2 (2 x 10 mL) and the organic phase was dried with anhydrous Na_2SO_4 and concentrated under reduced pressure. Mesitylene (0.23 mmol, 1.0 equiv) was added as an internal standard and the resulting mixture was analyzed by ^1H NMR spectroscopy.

Reaction at −78 °C. 16% yield of **2** was observed. When the reaction was kept at −78 °C, the color of the mixture rapidly changed from light yellow to purple, similar to when LDA is added to the quinoline **3**. The color remained the same during the entire reaction period (1 h) (Figure S4). The purple color suggests formation of lithiated quinoline **3a**. After the quench, approximately 16% yield of **2** was observed, with full mass balance (99%) across starting material and products.

Reaction at Room Temperature. 82% yield of **2** was observed. When reaction was carried out at room temperature, the first drops of LDA caused the purple color to form and disappear rapidly. By the end of the LDA addition, the purple color persisted for 5 to 10 min before becoming yellow again (Figure S4). ^1H NMR analysis showed the complete comsumption of the bedaquiline **1** at room temperature. Mass balance decreases from 99% for the −78 °C to 82% based on recovery of **2**, indicating that higher temperature can also favor other side reactions.

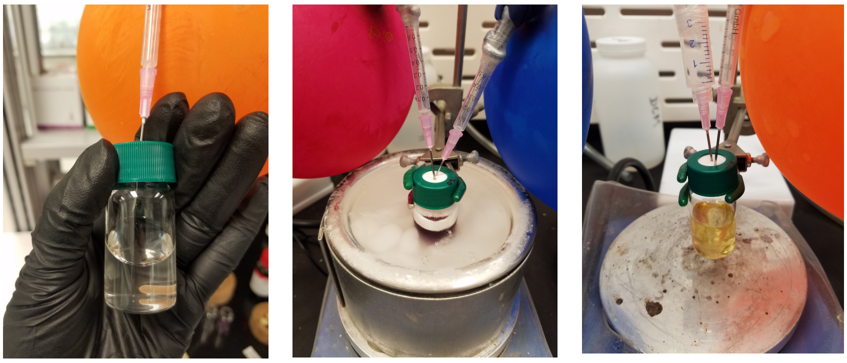

Figure S4. (left) Bedaquiline **1** in THF before addition of LDA; (middle) reaction at −78 °C; (right) reaction at room temperature

^{1}H NMR Spectroscopic Assays for Formation of 3a

Characterization of 3a (^{1}H NMR, THF-d_8) at –78 °C

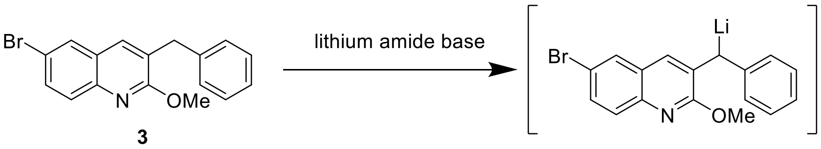

3 **3a**

Scheme S8. Illustration of formation of **3a**, reaction to be observed directly by ^{1}H NMR spectroscopy.

The NMR spectrometer used for this assay was a Bruker Avance Neo operating at 500.18 MHz. The spectrometer was cooled to –78 °C using a liquid nitrogen heat exchanger.

In a nitrogen-filled glovebox, an oven-dried 5 mm NMR tube equipped with a septum screw-cap was charged with a solution of **3** (25 mg, 0.08 mmol) in THF-d_8. A ^{1}H NMR spectrum was recorded at –78 °C and showed that the -OMe and -CH_2- signals at 4.03 and 4.04 ppm are observable but not well resolved. The resonance at 8.04 ppm (which corresponds to the C5 proton on the quinoline ring, or in other words the singlet ^{1}H situated *ortho*- to the -Br substituent) was chosen as a suitable handle to assay conversion of starting material on addition of LDA.

Characterization of 3a (^{1}H NMR, THF-d$_8$) at –78 °C

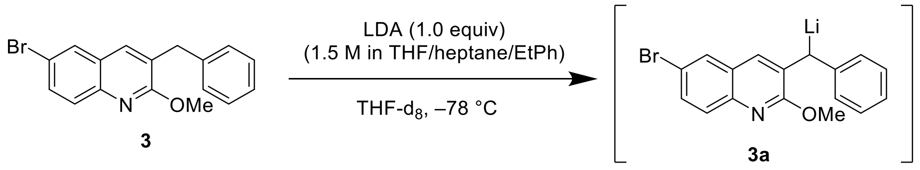

Scheme S9. Reaction of **3** with LDA to form **3a** in THF-d$_8$ for ^{1}H NMR analysis.

^{1}H NMR Analysis of Commercial LDA Solution (Figure S5, spectrum 1). An oven-dried 5 mm NMR tube was charged with THF-d$_8$ in a glovebox. The NMR tube was capped and removed from the glovebox and cooled to –78 °C in an IPA/dry ice bath. A commercial solution of LDA was added (50 µL of 1.6 M solution in THF/hexanes/PhEt, 0.07 mmol). This solution was maintained in an IPA/dry ice bath and transferred to a pre-cooled NMR spectrometer for analysis at –78 °C.

^{1}H NMR Analysis of 3a in THF-d$_8$ (Figure S5, spectra 2–4). A solution of **3** (23 mg, 0.07 mmol) in THF-d$_8$ (0.75 mL) was prepared in a glovebox. The solution was transferred to an oven-dried 5 mm NMR tube and removed from the glovebox. The sample was cooled to –78 °C under argon atmosphere and an initial ^{1}H NMR spectrum at –78 °C was recorded. The sample was removed from the spectrometer and returned to an IPA/dry ice bath. A commercial solution of LDA (0.07 mmol, 50 µL of 1.6 M solution in THF/heptane/PhEt, 1.0 equiv) was added and the solution was mixed by vortex with intermittent cooling in the IPA/dry ice bath until a red/black color was observed (about 10 cycles of mixing over 3 min). The resulting mixture was observed by ^{1}H NMR analysis at –78 °C at t = 8, 10, 18, 25, 30, and 40 min. After 40 min, the sample was removed from the spectrometer and mixed by vortexing, taking care to minimize the time the sample was outside of the IPA/dry ice bath. Note: it is assumed that significant warming of the sample occurred during this time. The sample was returned to the spectrometer at –78 °C, and observed by ^{1}H NMR analysis at t = 50 and 60 min. The signal for PhEt at 2.65 ppm was used as an internal standard to monitor relative conversion of starting material and formation of product over time. Note that in this ^{1}H NMR spectroscopy experiment a relaxation delay of 1.00 s was used, so quantification of signals is only a rough approximation.

Results. Upon analysis of the solution of **3a**, ^{1}H NMR signals at 5.1 ppm and 3.9 ppm were assigned as the -CHLi- and -OMe resonances for the lithiated quinoline product **3a** (see Figure S8). The assignment of these resonances was confirmed by ^{1}H-^{13}C HMBC. The spectra taken at different timepoints were analyzed to generate a curve showing conversion of **3** to **3a** over time (see Figure S6). This experiment showed that initial reaction of LDA with **3** is rapid but incomplete. Additional consumption of **3** was observed on mixing (warming) between t=40 min and t=50 min, however no additional **3a** was observed during the same time period.

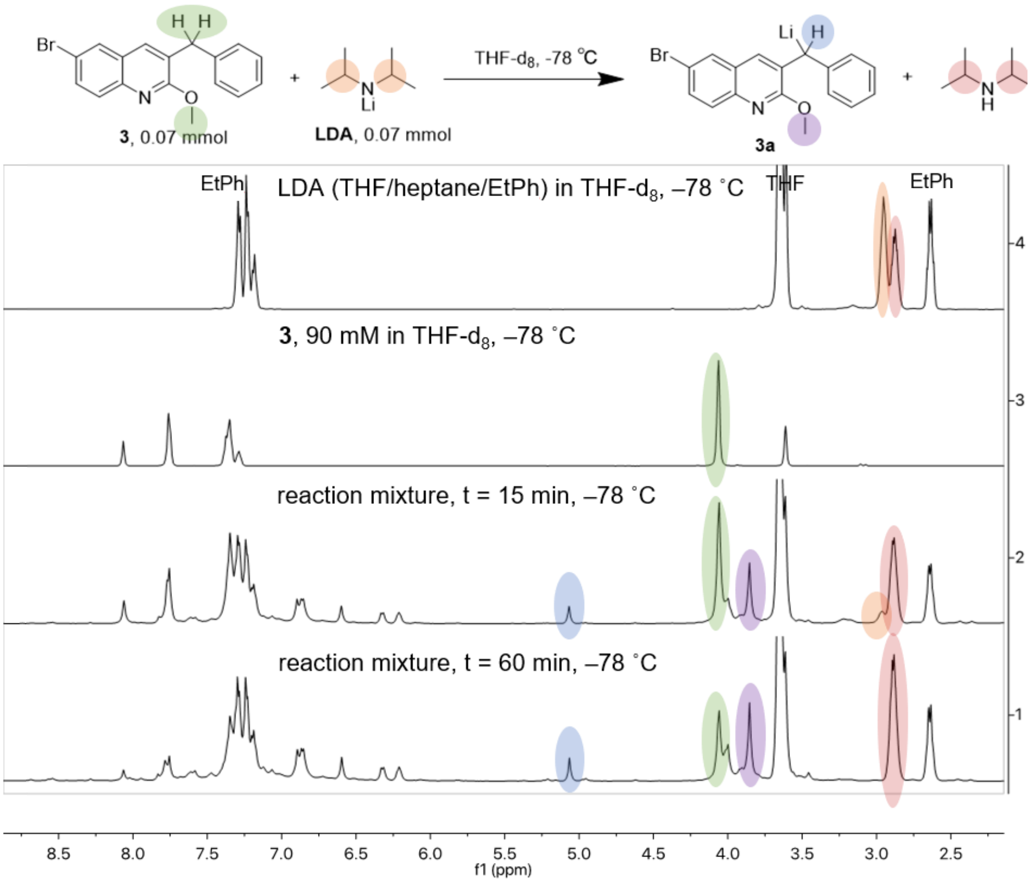

Figure S5. ^{1}H NMR spectra showing observation of **3a** at –78 °C and corresponding peak assignments.

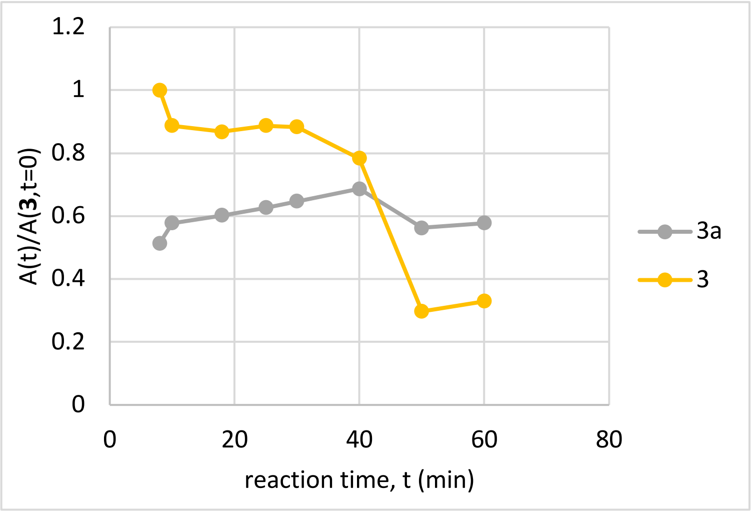

Figure S6. Illustration of ^{1}H NMR assay of time course of lithiation at –78 °C. Presence of **3** and **3a** measured by integration of ^{1}H NMR resonance at 8.04 ppm and 5.08 ppm, respectively, and reported as a ratio of absolute signal of these resonances at each timepoint (A(t)) vs. absolute signal of compound **3** at t = 0. The PhEt signal at 2.63 ppm was used as an internal standard to calibrate the absolute signal observed in each experiment. Note that a relaxation delay of 1 s was used in these ^{1}H NMR experiments, thus calculated values are approximations and significant error to the calculated values should be assumed.

Variable Temperature ^{1}H NMR Experiment for Reaction Mixture Containing 3a

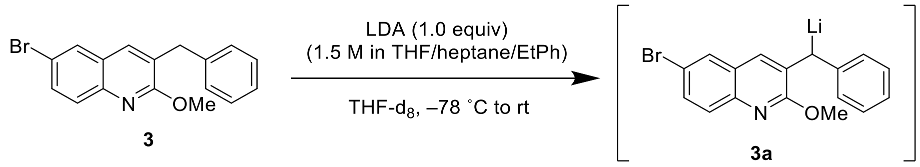

Scheme S10. Formation of **3a** by reaction of **3** with LDA in THF-d$_8$.

The experiments described above by Figure S5 and Figure S6 suggest that increasing temperature leads to lower mass balance of **3** + **3a**. To investigate the influence of temperature on the reaction mixture containing **3a**, a variable temperature ^{1}H NMR experiment was performed.

Experimental Procedure. A Bruker Avance Neo spectrometer operating at 500.18 MHz was cooled to –61 ˚C using a liquid nitrogen heat exchanger. Meanwhile, a 5 mm NMR tube equipped with a rubber septum was dried with a heat gun under vacuum, back-filled with argon, brought into a nitrogen-filled glovebox, and charged with a solution of **3** (23 mg, 0.07 mmol) in THF-d$_8$ (1 g). The sample was removed from the glovebox and cooled in an IPA/dry ice bath under inert atmosphere. A solution of LDA (0.07 mmol, 50 µL of 1.6 M commercial solution in THF/hexanes/PhEt) was added at –78 ˚C in an IPA/dry ice bath and the resulting solution was mixed by vortexing with intermittent cooling in the IPA/dry ice bath until a red/black color was observed (about 3 min).

Result. The dark red-black sample was analyzed by ^{1}H NMR spectroscopy at –61 ˚C and showed approximately 32% conversion of **3** and 7% of lithiated species **3a**. The sample was allowed to warm to room temperature inside the spectrometer, showing 58% conversion of **3** and 11% of lithiated species **3a** after 40 min. Warming does not improve conversion of **3** to **3a**. (see stacked spectra in NMR Spectra section)

Interpretation of Results. This confirmed the initial observation that conversion of **3** occurs on warming in the presence of LDA, but undesirable reactions occur and formation of **3a** is low.

^{1}H NMR Assay for 3a Formation from Lithium Pyrrolidide and 3 at Room Temperature

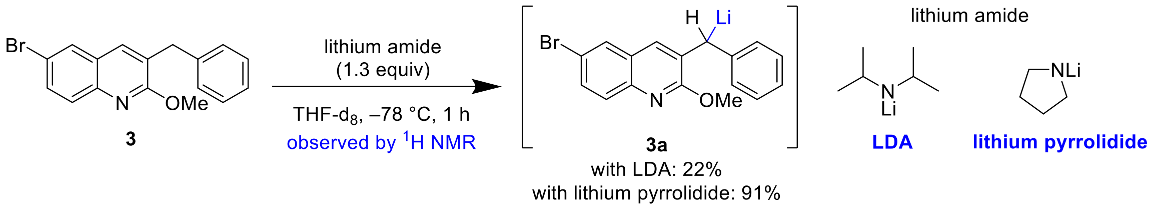

Scheme S11. Quantitative observation of **3a** by ^{1}H NMR analysis after treatment of **3** with LDA or lithium pyrrolidide.

Experimental Procedure. In a glovebox, a dry 2-dram vial was charged with a solution of **3** (50 mg, 0.15 mmol) and THF-d_8 (550 µL). A second 2-dram vial was charged with a magnetic stirrer and THF-d_8 (550 µL). The vials were capped and removed from the glovebox. To the second vial, mesitylene (21 µL, 0.15 mmol), followed by diisopropyl amine (32 µL, 0.23 mmol) was added, and the solution was cooled to –78 ˚C. A solution of *n*-BuLi, 2.6 M in hexanes (75 µL, 0.20 mmol) was added dropwise and the mixture was stirred for 10 min at –78 ˚C. The solution of **3** was added dropwise with stirring, then warmed to room temperature. The resulting dark red-black solution was transferred into an oven-dried and septum-capped 5mm NMR tube under inert atmosphere. The sample was then analyzed by ^{1}H NMR spectroscopy. 22% yield of **3a** was observed.

Following the above procedure, the same experiment was performed with pyrrolidine (19 µL, 0.23 mmol). 91% yield of **3a** was observed. The resulting spectra of reactive intermediate **3a** are included in the Spectra section.

Reaction Optimization

Changing the Identity of the Secondary Amine Base

General Procedure for Screening Secondary Amine Bases. An oven-dried 20 mL vial was charged with a stir bar, set under argon atmosphere and equipped with a septum cap. THF (3.3 mL) was added followed by pyrrolidine (80 µL, 0.98 mmol), and the solution was cooled to –40 ˚C with 60:40 ethylene glycol / water in a dry ice bath. *n*-BuLi (0.79 mmol, titrated commercial solution in hexanes) was added dropwise, resulting in a reaction concentration of 0.26 M. The resulting mixture was stirred for 20 min, then moved to rt and stirred for an additional 20 min. The solution was moved to an IPA / dry ice bath and cooled to –78 ˚C by stirring for 5 min. Concurrently, quinoline **3** (200 mg, 0.61 mmol) was weighed into an oven-dried 2-dram vial and dried under vacuum for 1 h, then dissolved in THF (3 mL). The room temperature solution of **3** was added dropwise to the prepared lithium amide solution at –78 ˚C resulting in a dark purple solution. The solution was stirred for 1 h –78 ˚C. Ketone **2** (166 mg, 0.73 mmol) was weighed into a dry 2-dram vial and dried under vacuum for 1 h. THF (3 mL) was added to dissolve, and the ketone was added dropwise –78 ˚C. After addition, the resulting mixture was stirred for an additional 30 min –78 ˚C. The reaction mixture was quenched at –78 ˚C by slow dropwise addition of 2 mL 25 wt% aqueous ammonium chloride solution. The solution was moved to room temperature after a sunflower yellow color was observed. On warming, the solution generally lightens to a sandy brown color. Benzyl benzoate (59 µL, 0.31 mmol) was added to the biphasic mixture and shaken to dissolve. The organic layer was sampled and the solvent was removed. The resulting residue was dissolved in $CDCl_3$ and analyzed by ^1H NMR spectroscopy using a 30 s relaxation delay. The yield was calculated by normalizing the signal at 5.4 ppm (benzyl benzoate CH_2) to 1.02, and reporting the resulting integral for the signals at 5.9 ppm (desired isomer, *RS*,*SR* – **1a**) and 5.8 ppm (undesired isomer, *RR*,*SS* – **1b**).

Table S5. Tabulated Results of Secondary Amine Base Screen.

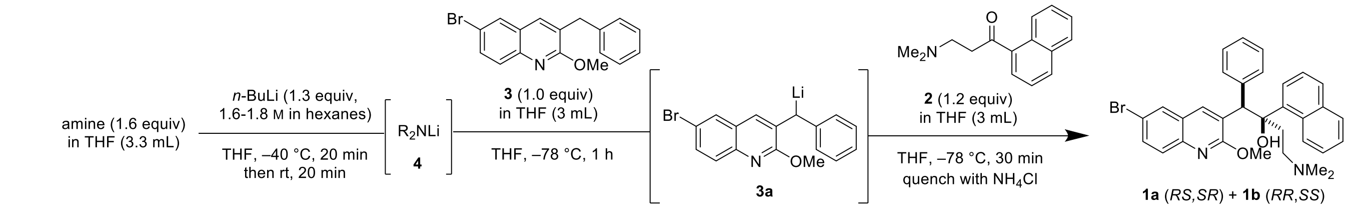

entry	amine	amine water content [mol %][a]	AY 1 [%][b]	d.r. [% and ratio, 1a:1b][b]
1	diisopropylamine	7	41	20:21 (1:1.1)
2	diisopropylamine	0.1	42	20:22 (1:1.1)
3	pyrrolidine	0.3	55	27:28 (1:1.0)
4	piperidine	2	42	20:22 (1:1.1)
5	morpholine	0.4	14	7:7 (1:1.0)
5 (repeat)	morpholine, new reagent bottle	-	28	12:16 (1:1.3)
6	diethylamine	-	32	14:18 (1:1.3)
7	2,2,6,6-tetramethylpiperidine	-	37	18:19 (1:1.1)
8	1-methylpiperazine	0.3	65	29:36 (1:1.2)
9	2-methypyrrolidine	1	35	17:18 (1:1.1)
10	*N,N′,N′*-trimethylethylenediamine	-	48	23:25 (1:1.1)
11	dicyclohexylamine	-	0	-
12	dimethylamine	2	53	26:27 (1:1.0)

AY = assay yield, [a] Determined by Karl Fischer titration of the amine prior to use

[b] Determined by ^1H NMR spectroscopy

Assaying Influence of Salt Additives on Diastereoselectivity

Experimental Procedure. In an oven dried Schlenk flask, LiBr (1.3 equiv) was dissolved in THF (0.5 mL) under argon atmosphere and LDA (0.6 M, 1.3 equiv, in THF, hexanes) was added dropwise. The solution was cooled to –78 °C and a solution of **3** (50 mg, 1.0 equiv) in THF (0.5 mL) was added dropwise. After stirring for 1 h at –78 °C, a solution of ketone **2** (42 mg, 1.2 equiv) in THF (1 mL) was added over 15 min and stirred for 30 min at –78 ˚C. The reaction was quenched by slow dropwise addition of sat. NH_4Cl aqueous solution (1 mL), followed by warming to room temperature after a yellow color is observed. CH_2Cl_2 and water were added and the organic phase was separated. The aqueous phase was extracted with CH_2Cl_2 (3x), the combined organic phases were dried over sodium sulfate and all volatiles were removed in vacuo. The d.r was determined by HPLC at 254 nm.

Table S6. Investigation of the influence of salt additives on diastereoselectivity of the 1,2-addition reaction

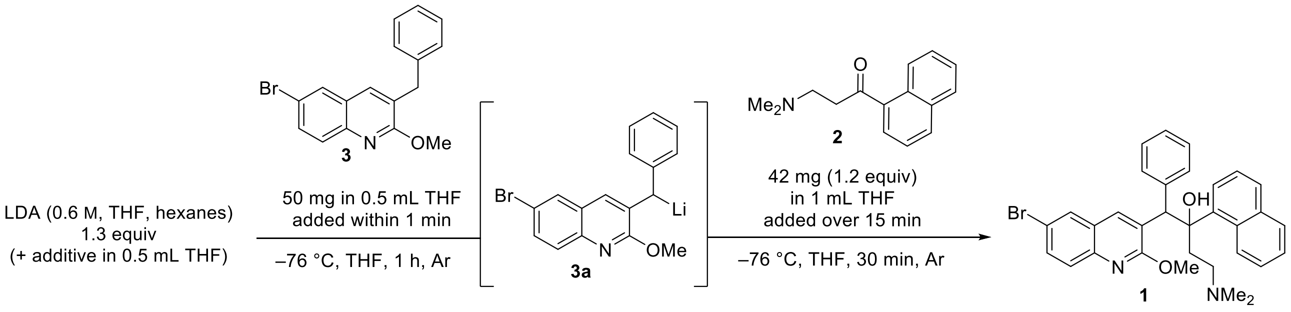

entry	additive	d.r.[a]
1	none	0.91 : 1.0
2	$MgBr_2{\cdot}OEt_2$	1.0 : 1.0
3	$ZnCl_2$	-[b]
4	LiBr	2.0 : 1.0
5	LiCl	1.2 : 1.0
6	LiI	1.1 : 1.0
7	LiBr (2-Me-THF)	-[c]
8	LiBr (DME)	0.4 : 1.0

[a] determined by HPLC (**1a** : **1b**) [b] no conversion (HPLC) [c] trace product (HPLC-MS)

Experimental Procedure for 50 mg Scale. An oven dried Schlenk flask was charged with the respective amine (1.5 equiv), a solution of LiBr (2.3 equiv) in THF (0.5 mL) under Ar-atmosphere. A solution of *n*-BuLi (1.9 M, 1.3 equiv) in hexanes was added slowly at 0 °C. The solution was cooled to –78 °C and a solution of **3** (50 mg, 1.0 equiv) in THF (0.5 mL) was added dropwise. After stirring for 1 h at –78 °C, a solution of ketone **2** (42 mg, 1.2 equiv) in THF (1 mL) was added over 15 min and stirred for 30 min at –78 ˚C. The reaction was quenched by slow dropwise addition of sat. NH_4Cl-solution (1 mL), followed by warming to room temperature after observation of a yellow color. CH_2Cl_2 and water were added and the organic phase was separated. The aqueous phase was extracted with CH_2Cl_2 (3x), the combined organic phases were dried over sodium sulfate and all volatiles were removed under vacuum. Yield and d.r. are determined by ^{1}H NMR spectroscopy using 1,4-bis(trimethylsilyl)benzene as internal standard.

Table S7. Impact of LiBr on diastereoselectivity in the 1,2-addition using different bases.

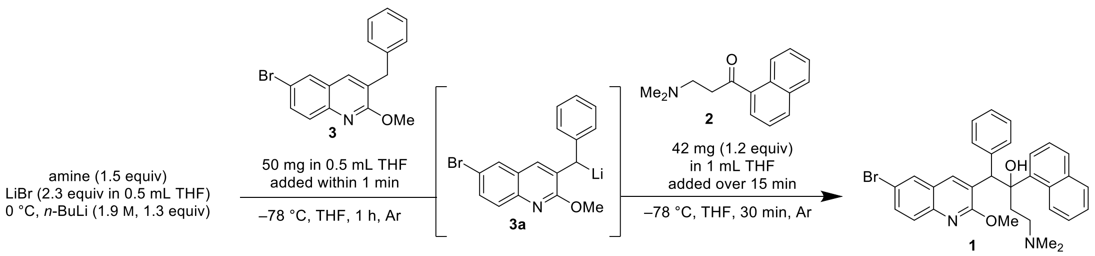

entry	amine	AY [%][a] 1a	AY [%][a] 1 (d.r.)[b]
1 [c]	diisopropylamine	25	37 (2.0 : 1.0)
2 [c]	dicyclohexylamine	0	0
3 [c]	pyrrolidine	53	74 (2.5 : 1.0)
4 [d]	pyrrolidine	57	80 (2.5 : 1.0)
5 [d]	dimethylamine	54	77 (2.4 : 1.0)
6 [d]	*N*-methylpiperazine	60	92 (1.9 : 1.0)
7 [d]	morpholine	61	87 (2.4 : 1.0)

[a] Determined by NMR using 1,4-bis(trimethylsilyl)benzene as internal standard.

[b] AY **1a** : AY **1b** [c] 50 mg scale [d] 200 mg scale

Table S8. Screening of LiBr equivalents using commercial LDA.

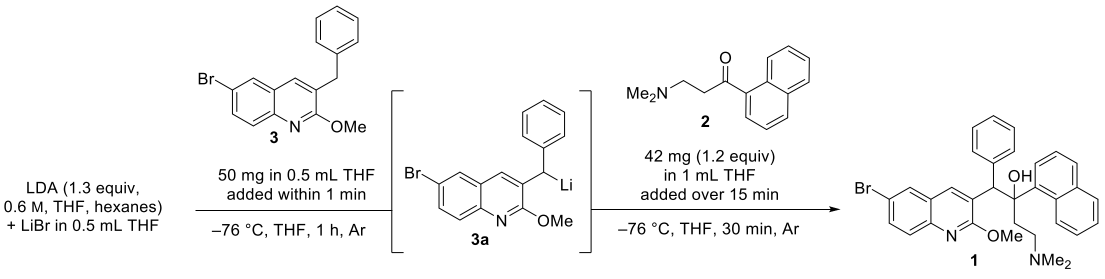

entry	LiBr [eq]	dr [1a:1b]	AY [%]
1[a]	0.6	1.1 : 1.0	-[c]
2[b]	1.1	1.8 : 1.0	36
3[b]	1.3	2.0 : 1.0	42
4[b]	1.5	2.0 : 1.0	43
5[b]	2.0	1.6 : 1.0	19
6[a]	2.6	1.3 : 1.0	-[c]

[a] Yield, d.r. determined by HPLC. [b] Yield, d.r. determined by ^{1}H NMR spectroscopy using 1,4-bis(trimethylsilyl)benzene as internal standard. [c] AY not determined.

Table S9. Synthesis of **1** at extended reaction times.

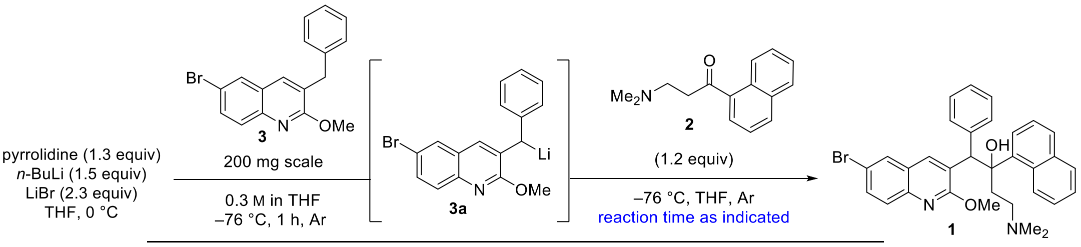

entry	Reaction time [h]	AY [%][a] 1a	AY 1 [%][a] (d.r)[b]
1	0.5	54	77 (2.5 : 1.0)
2	1.5	50	71 (2.5 : 1.0)
3	2	46	65 (2.4 : 1.0)

[a] Determined by NMR using 1,4-bis(trimethylsilyl)benzene as internal standard.
[b] AY **1a** : AY **1b.**

Table S10. Assaying influence of reaction time and temperature on yield and d.r. of **1**.

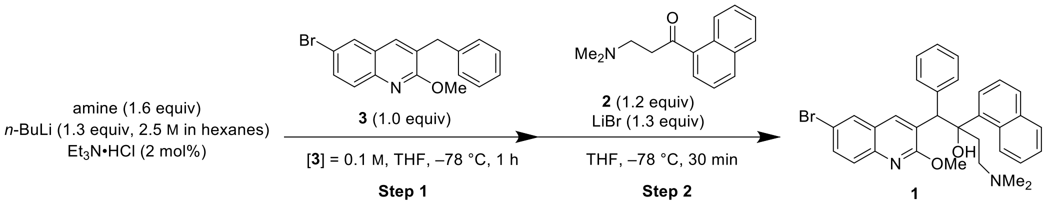

entry	base	variation	AY [%][a] 1a	AY [%][a] 1 (d.r.)[b]
1	pyrrolidine		45	69 (1.9 : 1.0)
2	pyrrolidine	t_2 = 4 min	45	68 (2.0 : 1.0)
3	*N*-methylpiperazine	t_2 = 4 min	48	75 (1.8 : 1.0)
4	*N*-methylpiperazine	T_1 = 23 °C<br>$t_1 = t_2$ = 2 min	48	74 (1.8 : 1.0)
5	morpholine	T_1 = 0 °C<br>$t_1 = t_2$ = 2 min	49	76 (1.8 : 1.0)

t = reaction time; T = reaction temperature
[a] Determined by ^{1}H NMR spectroscopy [b] AY **1a** : AY **1b**

General Procedure for Reaction Screening in Continuous Flow

Plug flow reactors (PFRs) were constructed from 0.03" ID high-purity PFA tubing and connected with IDEX fittings (PEEK nuts, unions, and T-mixers). For cooled reactions, reagent streams were equipped with a 100–200 µL precooling loop before joining other reagents at T-mixers. The precooling loops and PFRs were cooled using ice water or dry ice/isopropanol baths, or placed in room temperature water baths for room temperature reactions.

Preparation of quinoline **3** solution (**A**): To an oven-dried 10 mL volumetric flask with septum cap was added quinoline **3** (0.656 g, 2 mmol). The flask was placed under high vacuum for a minimum for 1 hour, then placed under argon. Anhydrous THF was added gradually while the flask was swirled until the solid had dissolved and the homogeneous solution reached 10 mL in volume.

Preparation of lithium pyrrolidide solution (**B**): To an oven dried 10 mL volumetric flask with stir bar and septum cap was added LiBr (0.399 g, 4.6 mmol) and/or $Et_3N{\cdot}HCl$ (0.006 g, 0.04 mmol) additives (as indicated). The flask was placed under high vacuum for a minimum of 1 hour, then placed under argon. Pyrrolidine (0.222 g, 0.256 mL, 3.1 mmol) and anhydrous THF (6 mL) were added with stirring and the flask was placed in an ice water bath. *n*-BuLi (nominally 1.6 M in hexanes, titrated[1] before use, 2.6 mmol) was added dropwise, followed by addition of anhydrous THF until the total volume reached 10 mL (stir bar was briefly lifted out of solution using a magnet to ensure accurate volume measurement). The solution was stirred at 0 °C for 20 min before using.

Preparation of ketone **2** solution (**C**): To an oven-dried 10 mL volumetric flask with septum cap was added ketone **2** (0.546 g, 2.4 mmol). The flask was placed under high vacuum for a minimum for 1 hour, then placed under argon. Anhydrous THF was added gradually while the flask was swirled until the ketone had dissolved and the homogeneous solution reached 10 mL in volume.

For each experiment, the reactors were flushed with anhydrous THF and cooled prior to equilibration. The reaction was allowed to equilibrate for 3 residence times before collection. Samples were collected on at least 0.1 mmol scale in a vial containing 1 M aqueous NH_4Cl solution. After collection, the layers were separated and the aqueous layer was extracted twice with THF (or CH_2Cl_2 for reactions using a methanol quench, to ensure clean phase separation). Benzyl benzoate was added as an NMR standard and the combined organic layers were dried by rotary evaporation followed by brief exposure to high vacuum. The entire sample was dissolved in $CDCl_3$ and a portion was taken for ^1H NMR assay yield determination. Additional details (reactor volumes and flow rates) of experiments presented in the main text are included in Table S11.

Table S11. Additional details of flow reaction in main text Table 2.

Table 2 Entry	Lithiation Reactor Volume (mL)	1,2-Addition Reactor Volume (mL)	Quench Reactor Volume (mL)	Flow Rate of A (mL/min)	Flow Rate of B (mL/min)	Flow Rate of C (mL/min)	Flow Rate of Quench (mL/min)
1, 2	2	2.5	0.2	0.1	0.1	0.1	0.2
3, 4	1	5	0.5	0.2	0.2	0.2	1
5	0.5	3	0.5	0.2	0.2	0.2	1
6	0.5	3	0.5	0.2	0.2	0.2	0.2
7	0.5	3	0.5	0.5	0.5	0.5	0.5

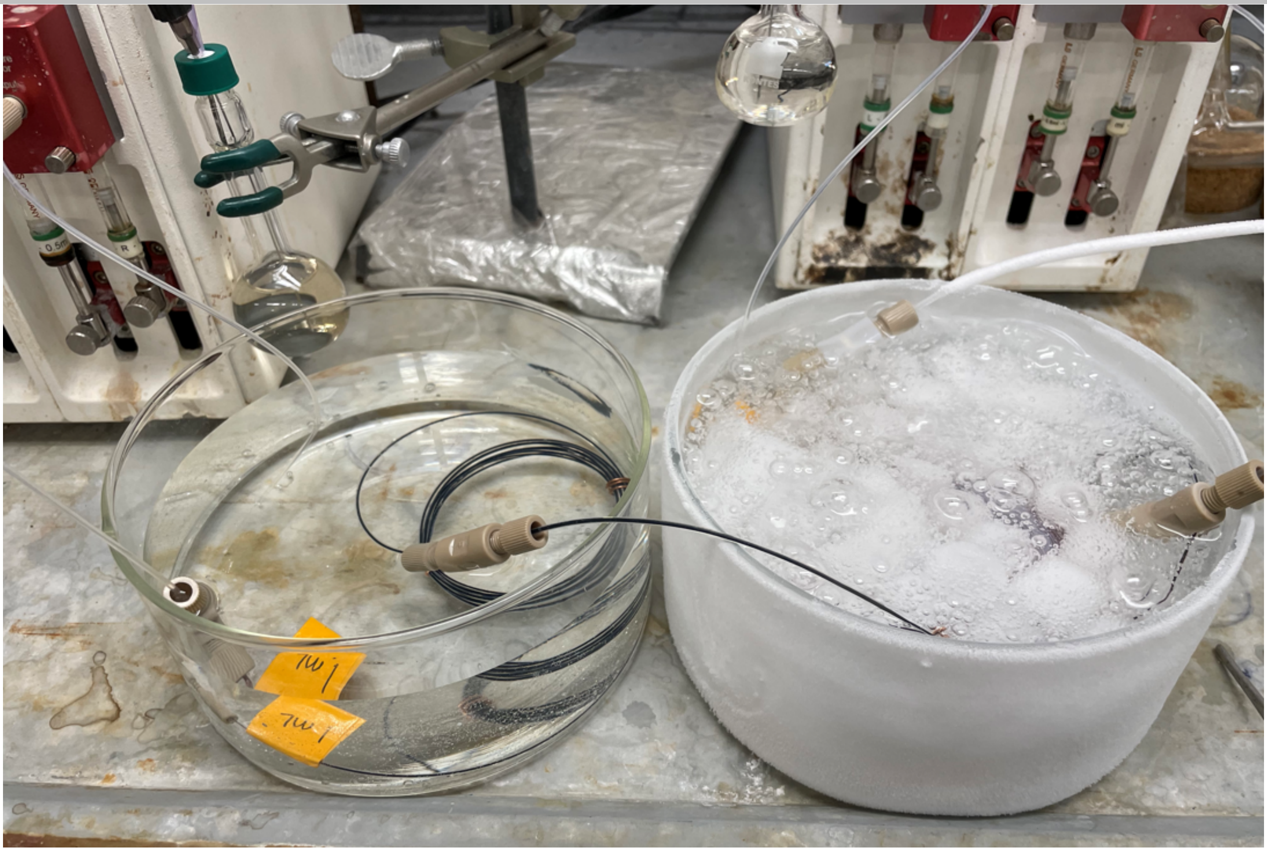

Figure S7. Continuous flow setup for synthesis of bedaquiline **1.** Left reactor coil shows characteristic color of formation of lithiated quinoline **3a** in a room temperature water bath. Right reactor coil submerged in IPA/dry ice bath for reaction of **3a** with **2** and subsequent reaction quench with MeOH.

Synthesis of (*RS*,*SR*)-1-(6-Bromo-2-methoxyquinolin-3-yl)-4-(dimethylamino)-2-(naphthalen-1-yl)-1-phenylbutan-2-ol (1a)

Experimental Procedure for Synthesis of (*RS*,*SR*)-1-(6-Bromo-2-methoxyquinolin-3-yl)-4-(dimethylamino)-2-(naphthalen-1-yl)-1-phenylbutan-2-ol (1a) on 1-gram Scale.
A flame-dried Schlenk flask (25 mL) was charged with a solution of LiBr (620 mg, 7.13 mmol, 2.3 equiv) in THF (5 mL) under argon atmosphere (gentle heating may be required for dissolving). The respective amine (pyrrolidine, *N*-methylpiperazine, or morpholine, 4.58 mmol, 1.5 equiv) was added, the solution was cooled in an ice bath and a solution of *n*-BuLi (1.3 equiv, 2.5 M in hexanes) was added slowly. After stirring for 15 min, the solution was cooled to –78 °C and quinoline (**3**, 1.00 g, 3.05 mmol, 1.0 equiv) in THF (5 mL) was added dropwise over 15 min. The mixture was stirred for 1 h at –78 °C. Afterwards, the ketone (**2**, 832 mg, 3.66 mmol, 1.2 equiv) was dissolved in anhydrous THF (3 mL) under argon atmosphere and added dropwise over 15 min to the deep purple reaction mixture. After 1 h, the reaction mixture was quenched by the slow addition of saturated aqueous NH_4Cl (2–3 mL). The cooling bath was removed and the two-phase mixture was transferred to a separatory funnel. The mixture was extracted with CH_2Cl_2 (3 x 10 mL) and the organic layers were dried over Na_2SO_4. The d.r. was determined by ^{1}H NMR spectroscopy. All volatiles were removed and the residue (**1**) was dissolved in 3–4 mL hot toluene. The solution was cooled slowly to room temperature and then refrigerated at 0–5 °C overnight. After refrigeration, the undesired diastereomer (**1b**) was filtered off and washed with 1–2 mL 0 °C toluene. The solvent was removed from the mother liquor and the residue was recrystallized two times from EtOH (3-4 mL). After filtration, washing with EtOH and drying in vacuo the desired diastereomer **1a** was obtained in yields up to 61% with d.r. up to 300 : 1 by ^{1}H NMR spectroscopy.

- Using morpholine (400 µL, 4.58 mmol), **1** was obtained with 2.4:1.0 d.r. prior to recrystallization. **1a** was obtained, (1.036 g, 1.87 mmol with >99% purity, 61%). **1b** was obtained (421 mg, 0.76 mmol, 25%, purity not measured).
- Using *N*-methylpiperazine (510 µL, 4.58 mmol), **1** was obtained with 1.8:1.0 d.r. prior to recrystallization. **1a** was obtained (1.011 g, 1.82 mmol with 99% purity, 60%)**.** **1b** was obtained (594 mg, 1.07 mmol, 35%, purity not measured).
- Using pyrrolidine (380 µL, 4.58 mmol), **1** was obtained with 2.4:1.0 d.r. prior to recrystallization. **1a** was obtained (942 mg, 1.70 mmol with >99% purity, 56%). **1b** was obtained (478 mg, 0.86 mmol, 28%, purity not measured).

Representative Experimental Procedure for Synthesis of (*RS*,*SR*)-1-(6-Bromo-2-methoxyquinolin-3-yl)-4-(dimethylamino)-2-(naphthalen-1-yl)-1-phenylbutan-2-ol (1a) as described in Table S12 on 5 g and 10 g Scale with 6 V or 10 V Solvent (example described here: 5 g, 10 V). In an oven-dried round-bottom flask charged with a stir bar, freshly distilled pyrrolidine (1.90 mL, 22.9 mmol, 1.5 equiv), anhydrous LiBr (610 mg, 35.1 mmol, 2.3 equiv) and anhydrous THF (15 mL, 3 V) were placed under N_2 atmosphere. After LiBr was completely dissolved, the reaction mixture was cooled to 0 ºC and titrated *n*-BuLi (19.8 mmol, 1.3 equiv) was added dropwise. After 20 min, the round-bottom flask was transferred to a −78 ºC bath (acetone/dry ice) and a solution of quinoline **3** (5.005 g, 15.2 mmol, 1.0 equiv) in anhydrous THF (25 mL, 5 V) was added dropwise by syringe pump (0.42 mL/min) over an 1 h period. Then a solution of ketone **2** (4.150 g, 19.29 mmol, 1.2 equiv) in anhydrous THF (10 mL, 2 V) was added into the reaction mixture dropwise by syringe pump (0.17 mL/min) at the same temperature. After the whole volume of the ketone **2** solution was added, the reaction was stirred for another 15 min, and then quenched by using 10 mL (2 V) aq. NH_4Cl (dropwise) at −78 ºC. The reaction mixture was directly poured into a separatory funnel. Water was added (20 mL) and the resultant mixture was extracted with CH_2Cl_2 (3 x 30 mL). The combined organic layers were concentrated under reduced pressure and purified by silica gel column chromatography or crystallization. Results of several representative experiments are tabulated below in Table S12.

Analytical Data of the (*RS,SR*)-Diastereomer 1a:
Habitus: colorless solid.
R_f: 0.35 (^{c}Hex/EtOAc 1:1)
Melting point: 193.4–193.6 °C.
IR: 2980, 2949, 2858, 2823, 2782, 1616, 1598, 1566, 1511, 1081, 753.
MS (ESI): *m/z* (%) = 555.2 (100), 557.2 (97) $[M+H]^+$.
^{1}H-NMR, COSY (300 MHz chloroform-d_1): δ/ppm = 8.90 (s, 1H), 8.61 (d, *J* = 8.8 Hz, 1H), 8.36 (s, 1H), 7.97 (d, *J* = 2.2 Hz, 1H), 7.94–7.85 (m, 2H), 7.75–7.58 (m, 4H), 7.52–7.44 (m, 1H), 7.36–7.27 (m, 1H), 7.17–7.09 (m, 2H), 6.94–6.86 (m, 3H), 5.89 (s, 1H), 4.21 (s, 3H), 2.67–2.41 (m, 1H), 2.17–1.88 (m, 9H).
^{13}C-NMR, HSQC, HMBC (75 MHz, chloroform-d_1): δ/ppm = 161.5, 143.9, 141.8, 140.7, 138.9, 134.8, 132.1, 130.1, 130.0, 129.9, 128.7, 128.3, 128.0, 127.5, 127.3, 127.0, 125.9, 125.4, 125.3, 125.2, 124.6, 117.1, 82.7, 56.5, 54.3, 49.7, 44.8, 33.6.
The analytical data are consistent with those reported in the literature.[7]

Table S12. Scale-up of bedaquiline **1** – variation of reaction concentration and use of *n*-BuLi solution with different concentrations.

entry	Solvent V_{total}	*n*-BuLi [M]	scale of 3 (g)	AY 1 (1a + 1b) [%][a]	d.r. ratio (1a : 1b)[a]	Yield 1 [%][b]	Yield 1a [g, mmol, % purity][a]	Yield 1a [%][c]	Yield 1b [g, mmol, % purity][a]	Yield 1b [%][c]
1[d]	10 V	2.45	5.0	87 (60 + 27)	2.2 : 1.0	79	3.97, 7.15, 90	42	1.59, 2.86, 84	16
2	10 V	1.40	5.0	90 (60 + 30)	2.0 : 1.0	80	5.31, 9.56, 90	56	2.12, 3.82, 92	23
3	10 V	1.40	10.0	90 (61 + 29)	2.1 : 1.0	77	11.61, 20.9, 88	60	3.21, 5.78, 90	17
4	6 V	1.40	5.0	88 (57 + 31)	1.8 : 1.0	73	5.05, 9.09, 91	54	1.75, 3.15, 91	18
5	6 V	2.40	10.0	88 (58 + 30)	1.9 : 1.0	75	10.02, 18.0, 87	52	4.28, 7.70, 91	23

[a] Assay yields, d.r., and purity determined by ^{1}H NMR spectroscopy, triphenylmethane as internal standard
[b] Yield = yield of isolated product. Yield **1** = Yield **1a** + Yield **1b**, corrected for purity
[c] Calculated by the product of isolated mass and purity
[d] purified by column chromatography. See further procedural notes below.

Conditions for column chromatography (5–10 g scale): Silica gel stationary phase, mobile phase 9:1 hexanes:EtOAc to 1:1 hexanes:EtOAc. See Table S12, entry 1 for details. Pure compound **1** was obtained as three fractions after chromatography, one of which contained a mixture of isomers **1a** and **1b**. Total combined yield of **1** (**1a**+**1b**) (7.77 g, 14.0 mmol, 83–90% purity, 80% yield of **1**). The fraction containing **1a** was isolated (3.97 g, 7.15 mmol, with 90% purity, 42% yield **1a**). The fraction containing **1b** was isolated (1.59 g, 2.86 mmol, with 84% purity, 16% yield **1b**).

Experimental procedure for crystallization (5–10 g scale): In a round-bottom flask containing the crude product, 4 V of toluene was added, and the resulting suspension heated to reflux for 10 min until fully dissolved. The solution was cooled to room temperature and refrigerated at 0–5 °C overnight. The pale-yellow solid was filtered and washed with toluene to obtain pure **1b**. The mother liquor was concentrated to dryness and the resulting residue was dissolved in 4 V of ethanol and stirred for 3 h at room temperature. The solid was filtered off and washed with cold ethanol to obtain **1a**. The final mother liquor contained only unreacted starting materials **2** and **3** by ^{1}H NMR spectroscopy.

Note. When crystallization was used as the isolation technique on >5 g scale, obtaining pure **1a** was challenging. When reaction was performed on 5.0 g scale (Table S12, Entry 2), the first solid fraction from toluene crystallization yielded pure **1b** (2.120 g, 30% yield), and the second solid fraction from ethanol crystallization yielded a mixture of both diastereomers (5.310 g, 60% yield, 14:1 d.r. of **1a** : **1b**). For the 10 g scale reaction (Entry 3), pure **1b** was obtained in 29% yield (3.210 g), and the second solid fraction corresponded to a mixture possessing 6:1 d.r. of **1a** : **1b** (11.612 g, 61% yield).

^{1}H and ^{13}C NMR Spectra

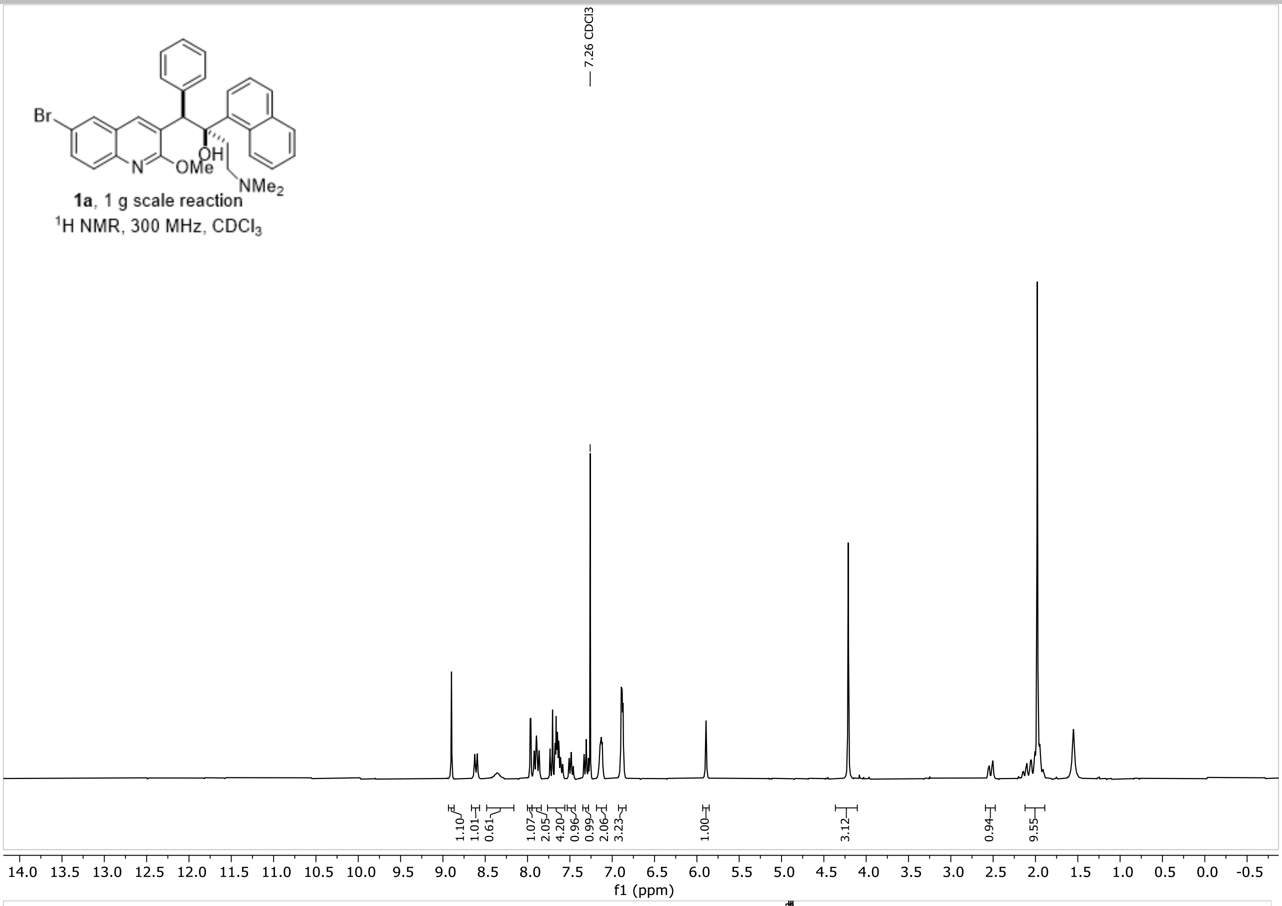
7.26 CDCl3
Br
N
OMe
OH
NMe2
1a, 1 g scale reaction
1H NMR, 300 MHz, CDCl3
1.10
1.01
0.61
1.07
2.05
4.20
0.96
0.99
2.06
3.23
1.00
3.12
0.94
9.55
14.0 13.5 13.0 12.5 12.0 11.5 11.0 10.5 10.0 9.5 9.0 8.5 8.0 7.5 7.0 6.5 6.0 5.5 5.0 4.5 4.0 3.5 3.0 2.5 2.0 1.5 1.0 0.5 0.0 -0.5
f1 (ppm)

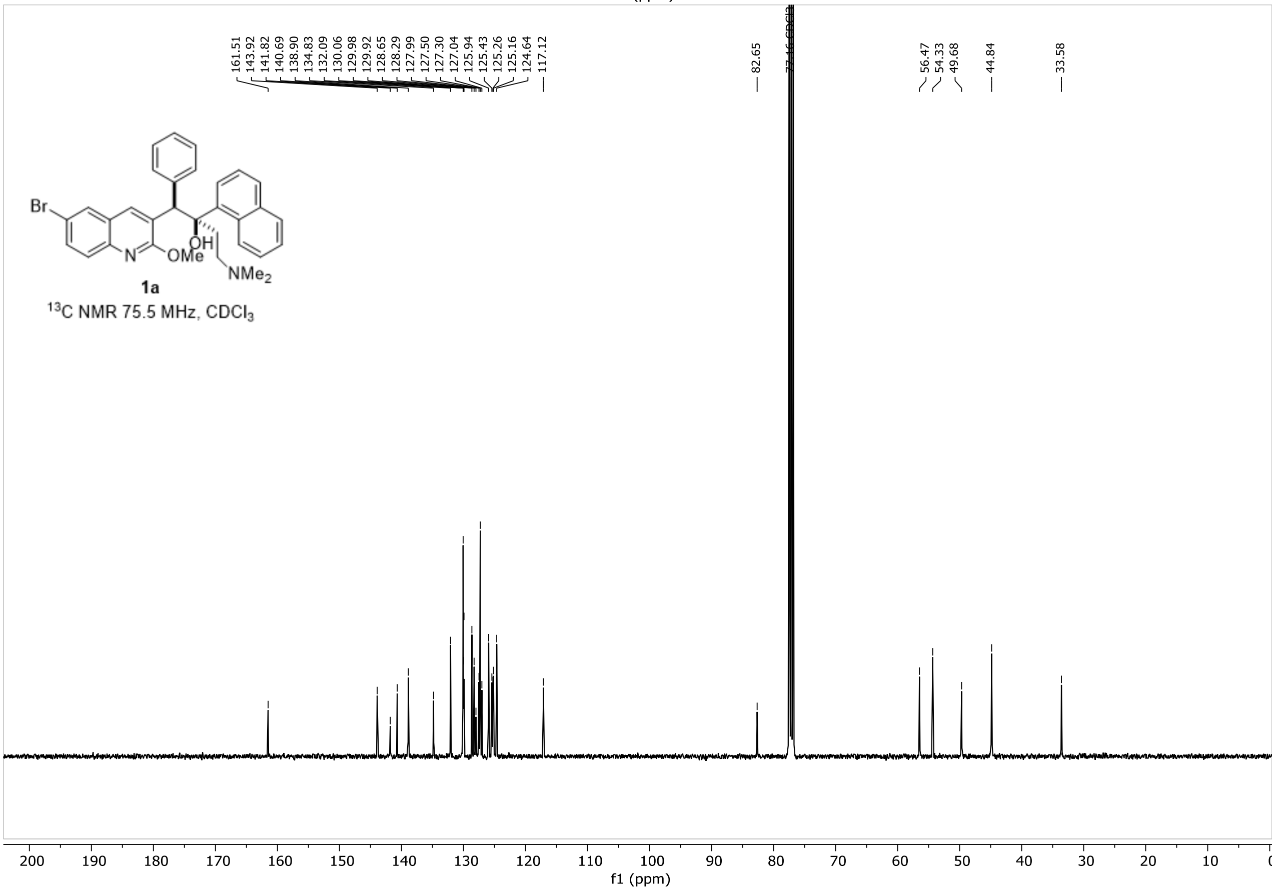
161.51
143.92
141.82
140.69
138.90
134.83
132.09
130.06
129.98
129.92
128.65
128.29
127.99
127.50
127.30
127.04
125.94
125.43
125.26
125.16
124.64
117.12
82.65
77.16 CDCl3
56.47
54.33
49.68
44.84
33.58
Br
N
OMe
OH
NMe2
1a
13C NMR 75.5 MHz, CDCl3
200 190 180 170 160 150 140 130 120 110 100 90 80 70 60 50 40 30 20 10
f1 (ppm)

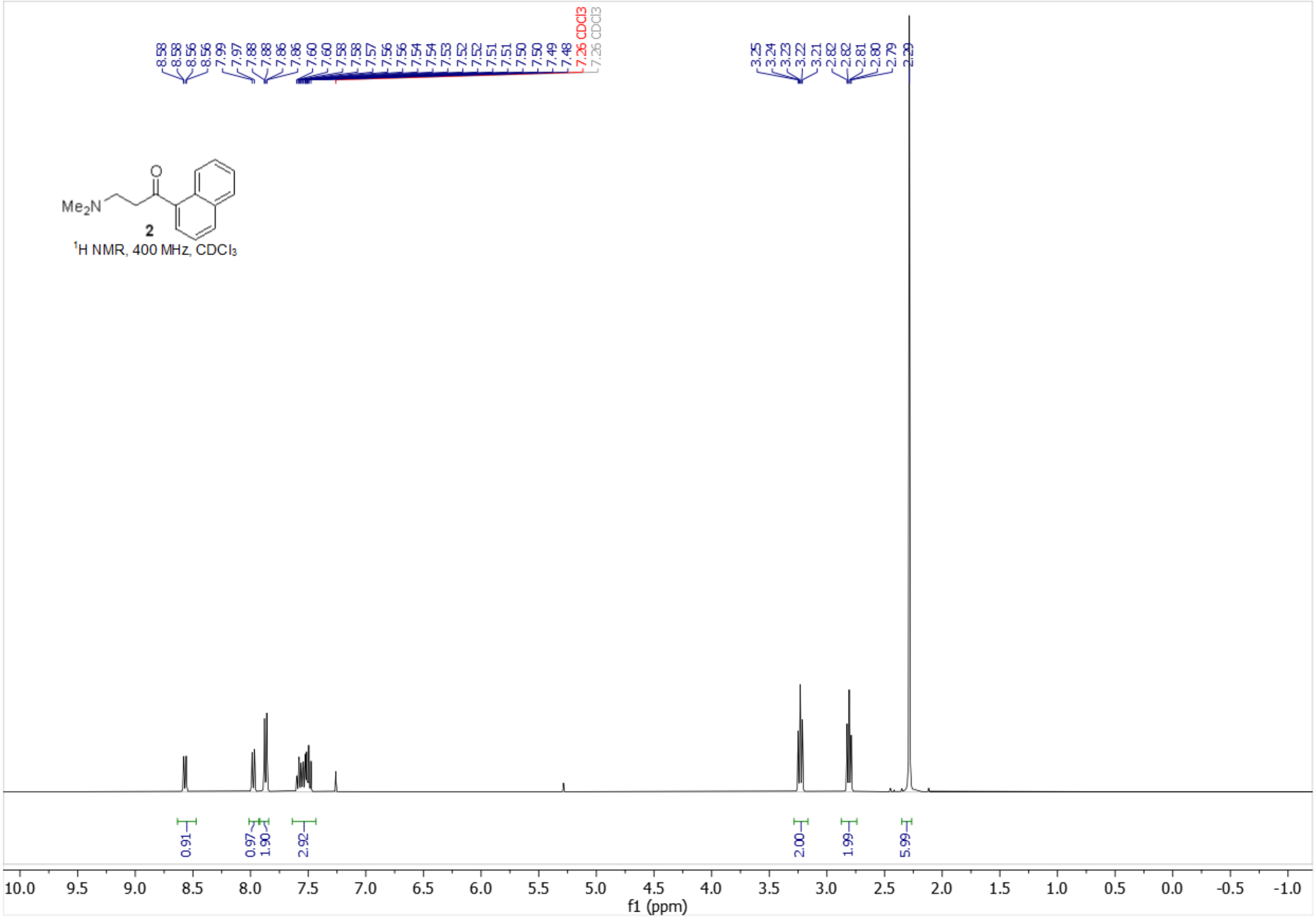
Me2N
2
1H NMR, 400 MHz, CDCl3
f1 (ppm)

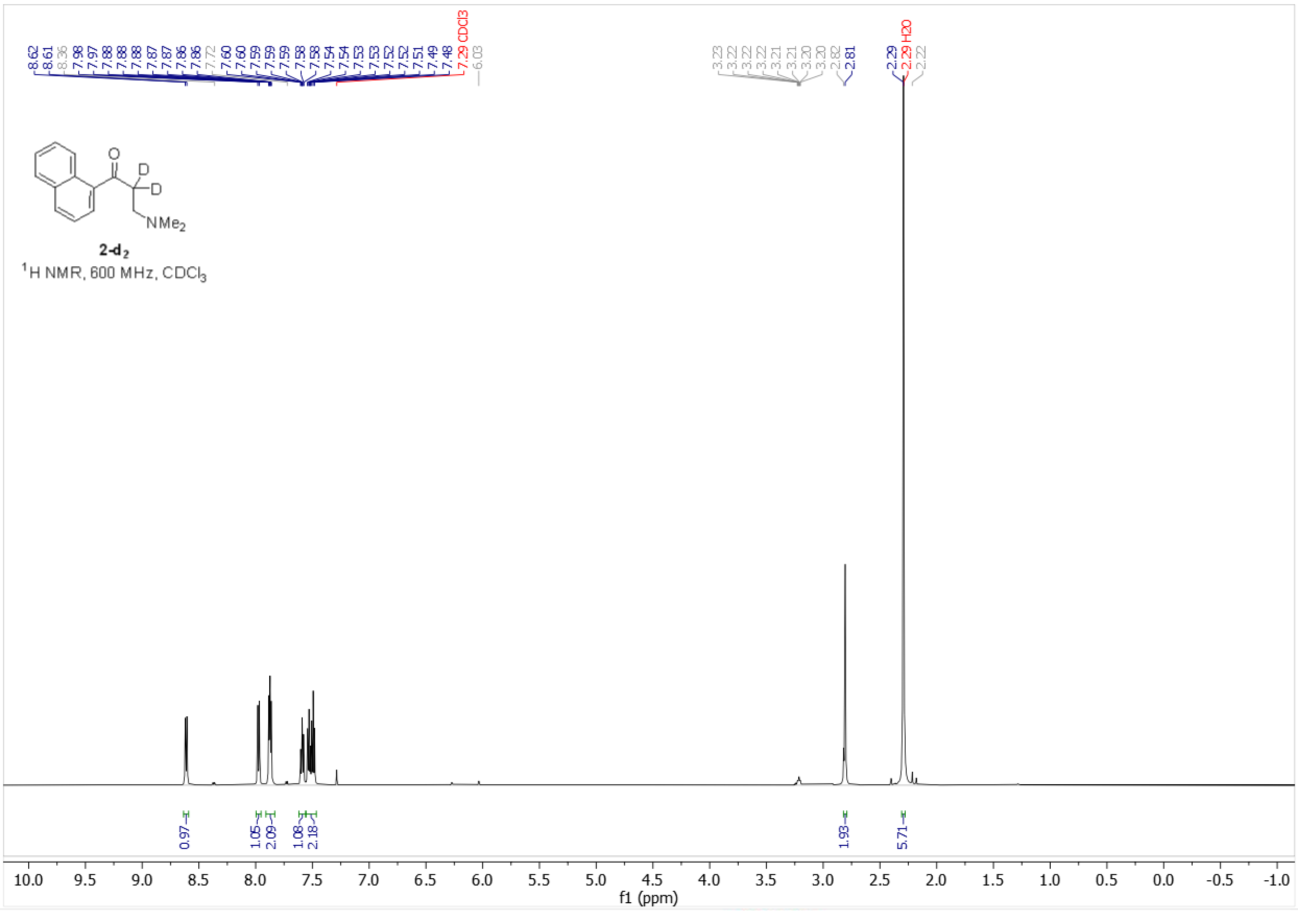
2-d2
^{1}H NMR, 600 MHz, CDCl3
7.29 CDCl3
2.29 H2O
f1 (ppm)

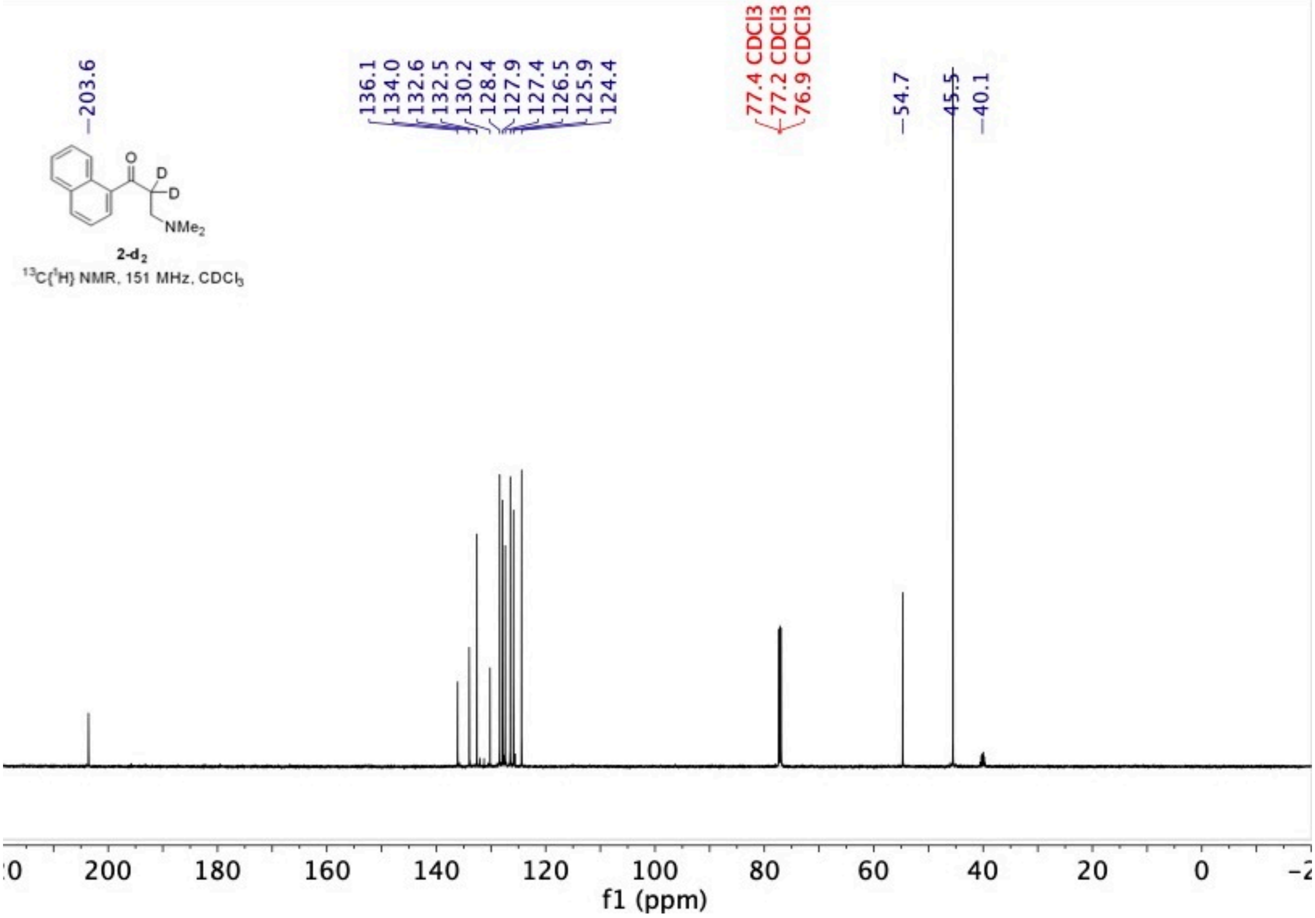
2-d2
^{13}C{^{1}H} NMR, 151 MHz, CDCl3
203.6
136.1
134.0
132.6
132.5
130.2
128.4
127.9
127.4
126.5
125.9
124.4
77.4 CDCl3
77.2 CDCl3
76.9 CDCl3
54.7
45.5
40.1
f1 (ppm)

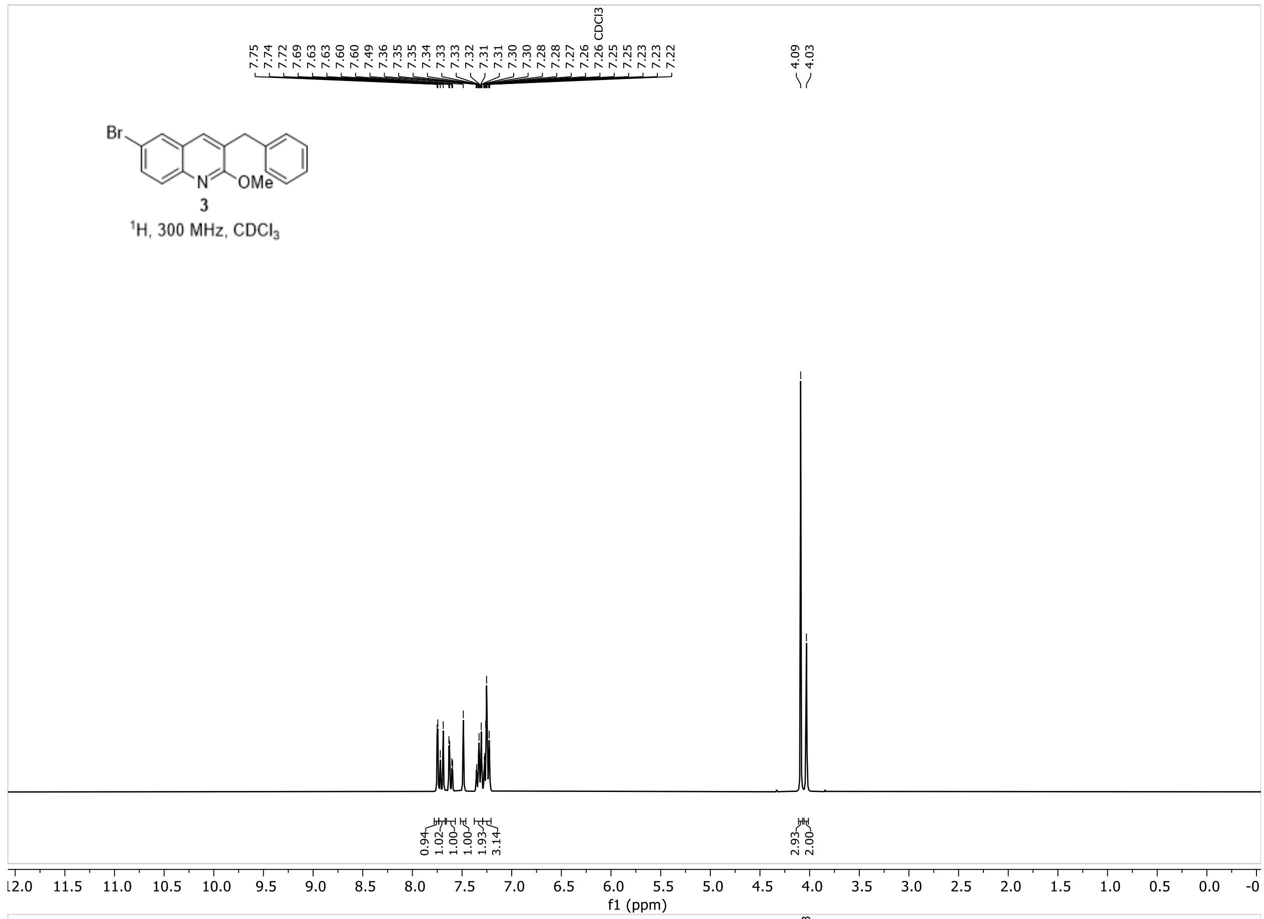
Br
N
OMe
3
^{1}H, 300 MHz, CDCl$_3$
f1 (ppm)

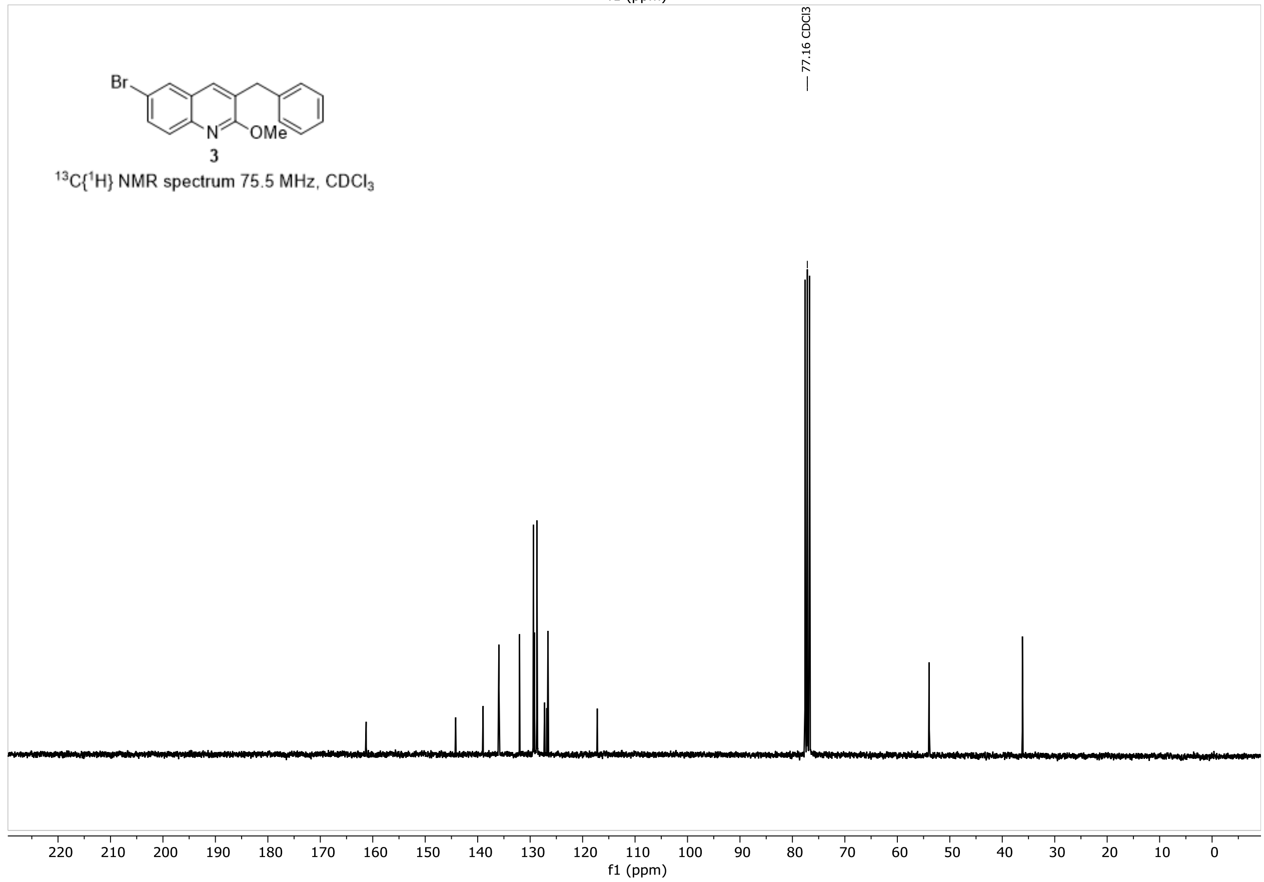
Br
N
OMe
3
^{13}C{^{1}H} NMR spectrum 75.5 MHz, CDCl$_3$
77.16 CDCl3
f1 (ppm)

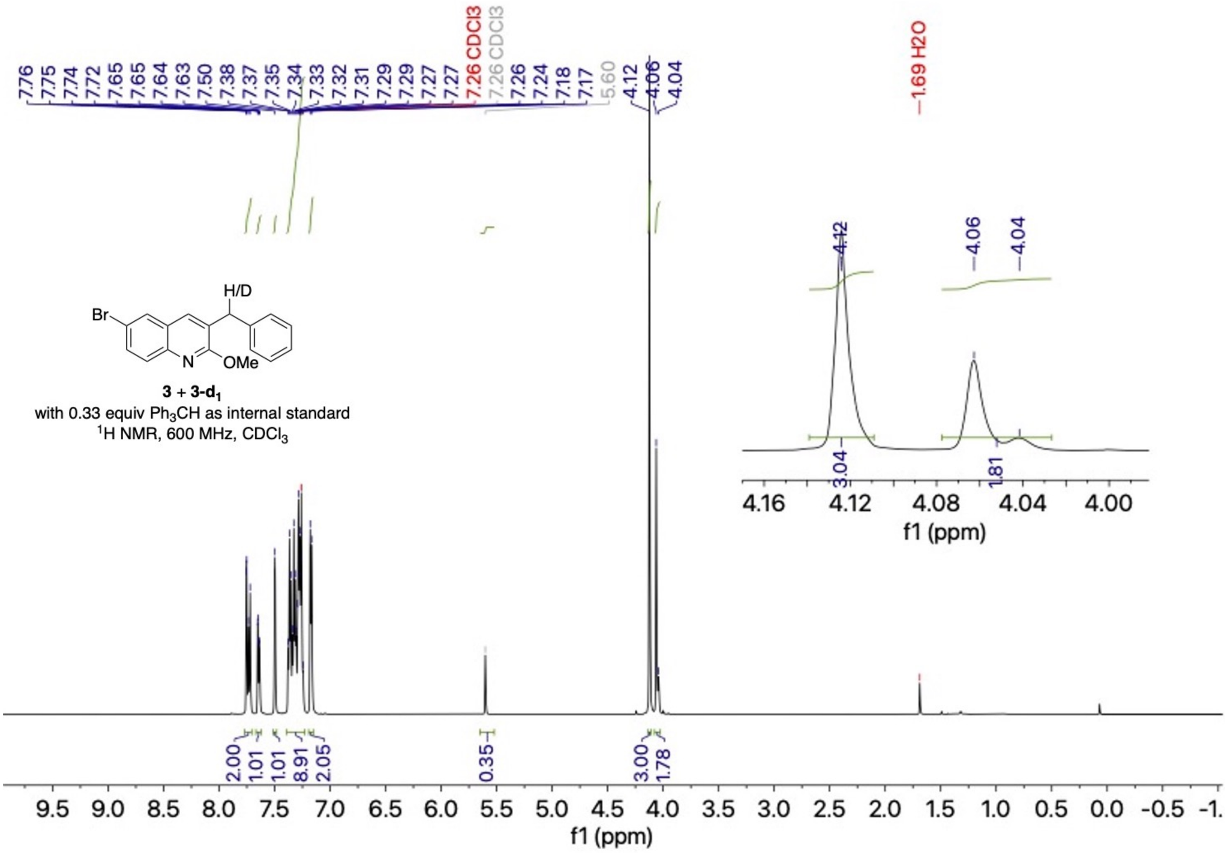
3 + 3-d1
with 0.33 equiv Ph3CH as internal standard
1H NMR, 600 MHz, CDCl3
f1 (ppm)

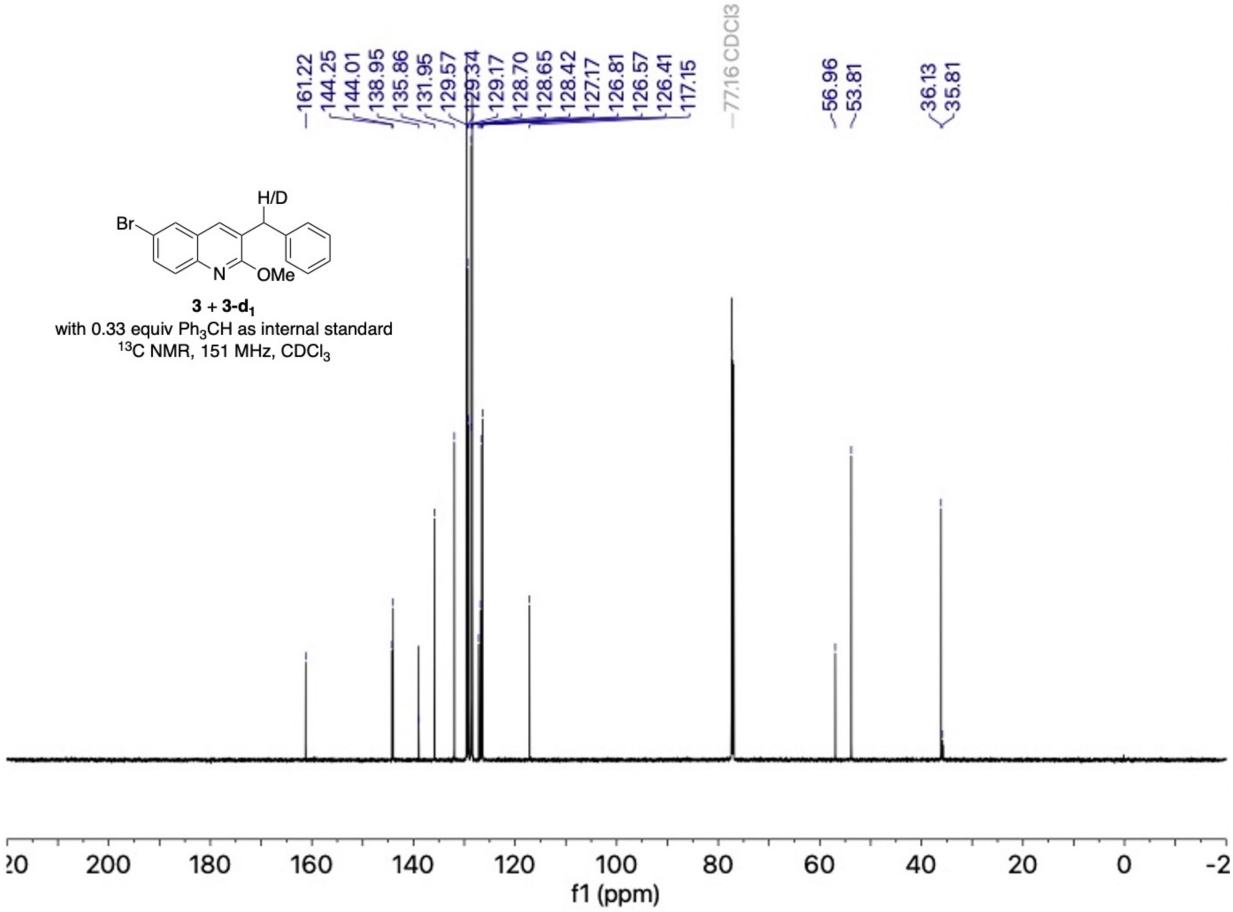
3 + 3-d1
with 0.33 equiv Ph3CH as internal standard
13C NMR, 151 MHz, CDCl3
f1 (ppm)

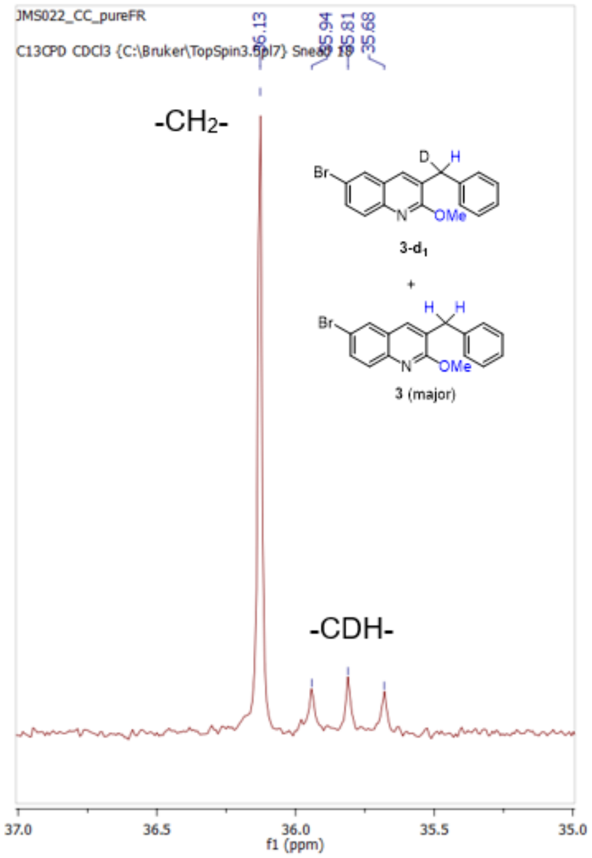
JMS022_CC_pureFR
C13CPD CDCl3 {C:\Bruker\TopSpin3.5pl7} Snead 18
36.13
35.94
35.81
35.68
-CH2-
-CDH-
Br
D
H
N
OMe
3-d1
+
H
H
3 (major)
f1 (ppm)

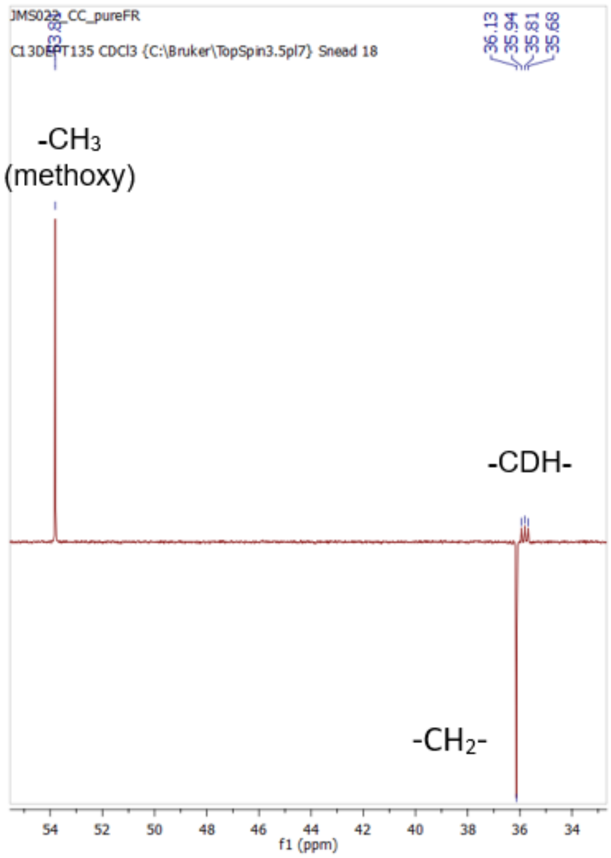
JMS022_CC_pureFR
C13DEPT135 CDCl3 {C:\Bruker\TopSpin3.5pl7} Snead 18
36.13
35.94
35.81
35.68
-CH3
(methoxy)
-CDH-
-CH2-
f1 (ppm)

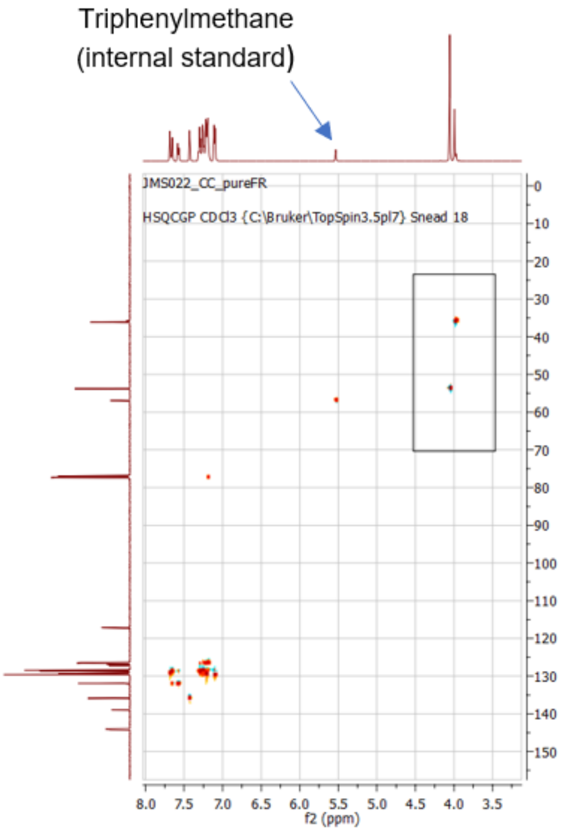
Triphenylmethane
(internal standard)
JMS022_CC_pureFR
HSQCGP CDCl3 {C:\Bruker\TopSpin3.5pl7} Snead 18
f2 (ppm)
f1 (ppm)

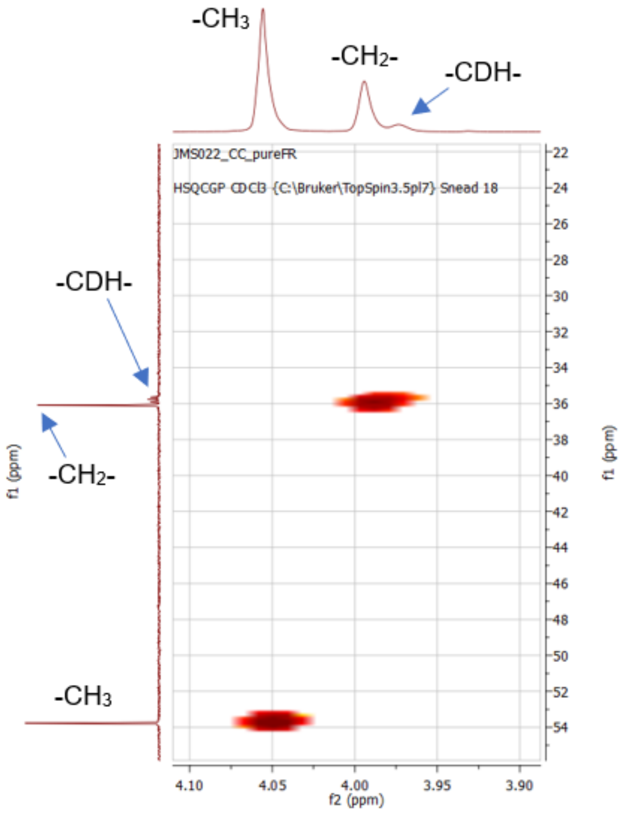
-CH3
-CH2-
-CDH-
JMS022_CC_pureFR
HSQCGP CDCl3 {C:\Bruker\TopSpin3.5pl7} Snead 18
-CDH-
-CH2-
-CH3
f2 (ppm)
f1 (ppm)

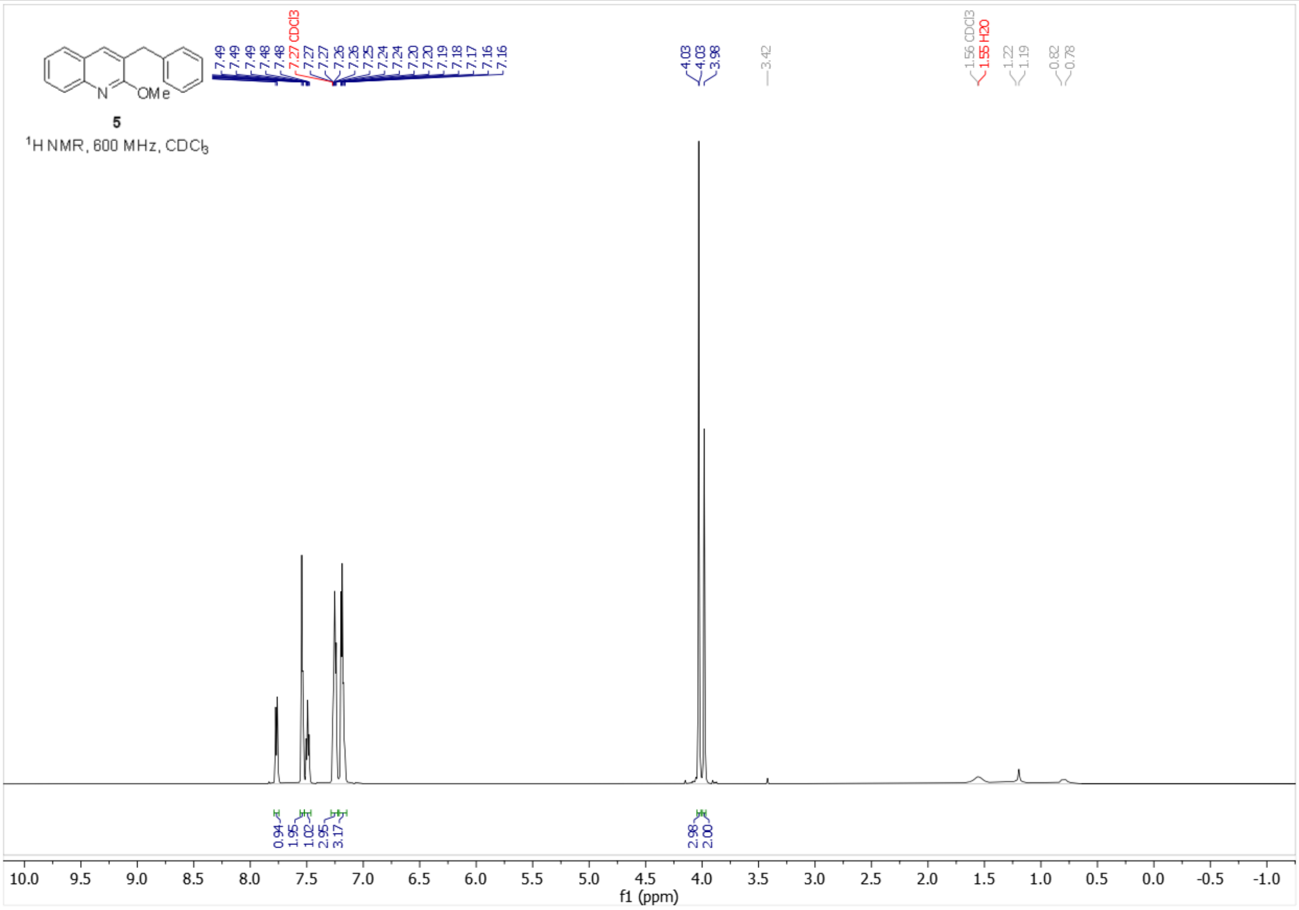
5
¹H NMR, 600 MHz, CDCl₃
f1 (ppm)

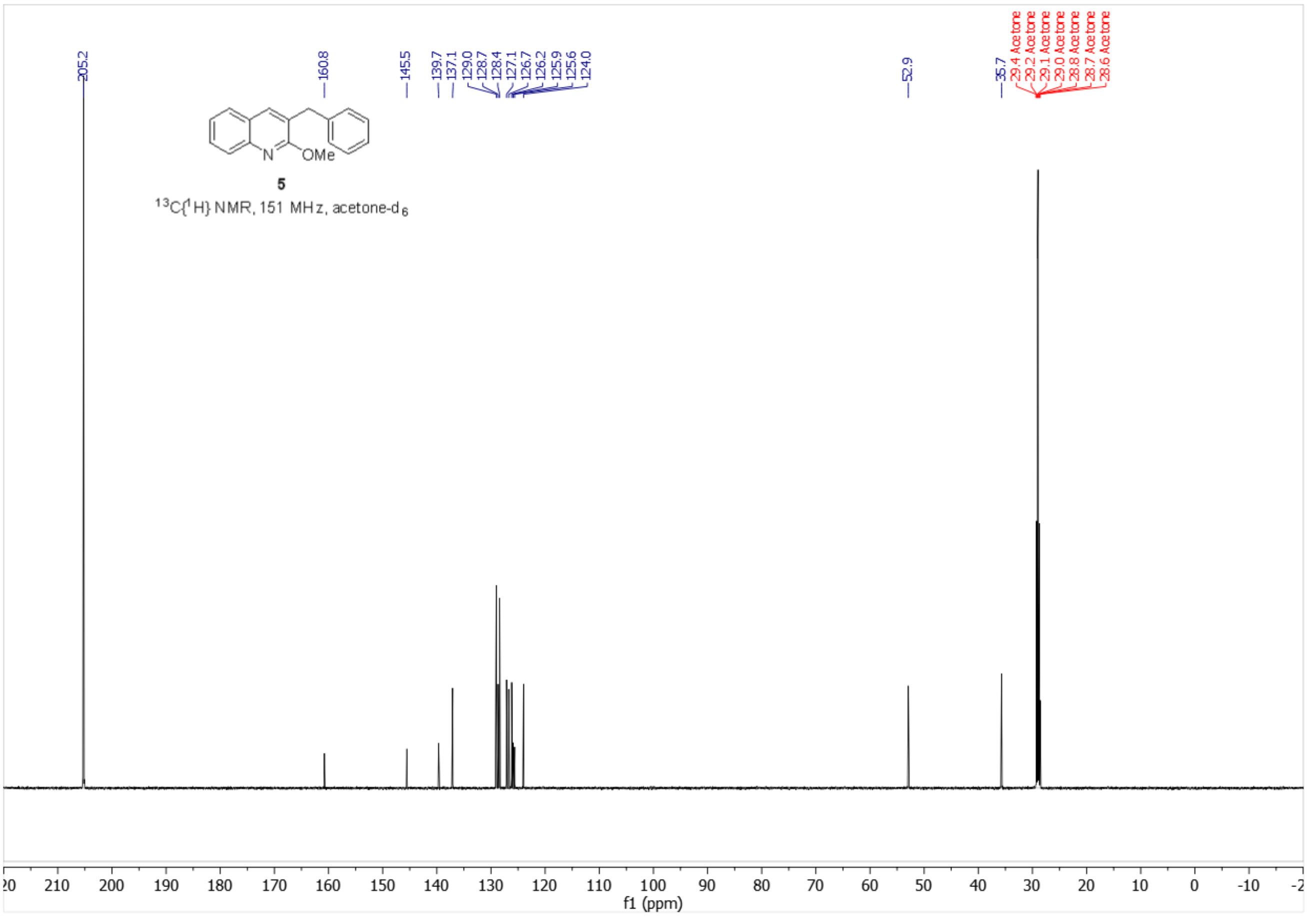
5
¹³C{¹H} NMR, 151 MHz, acetone-d₆
f1 (ppm)

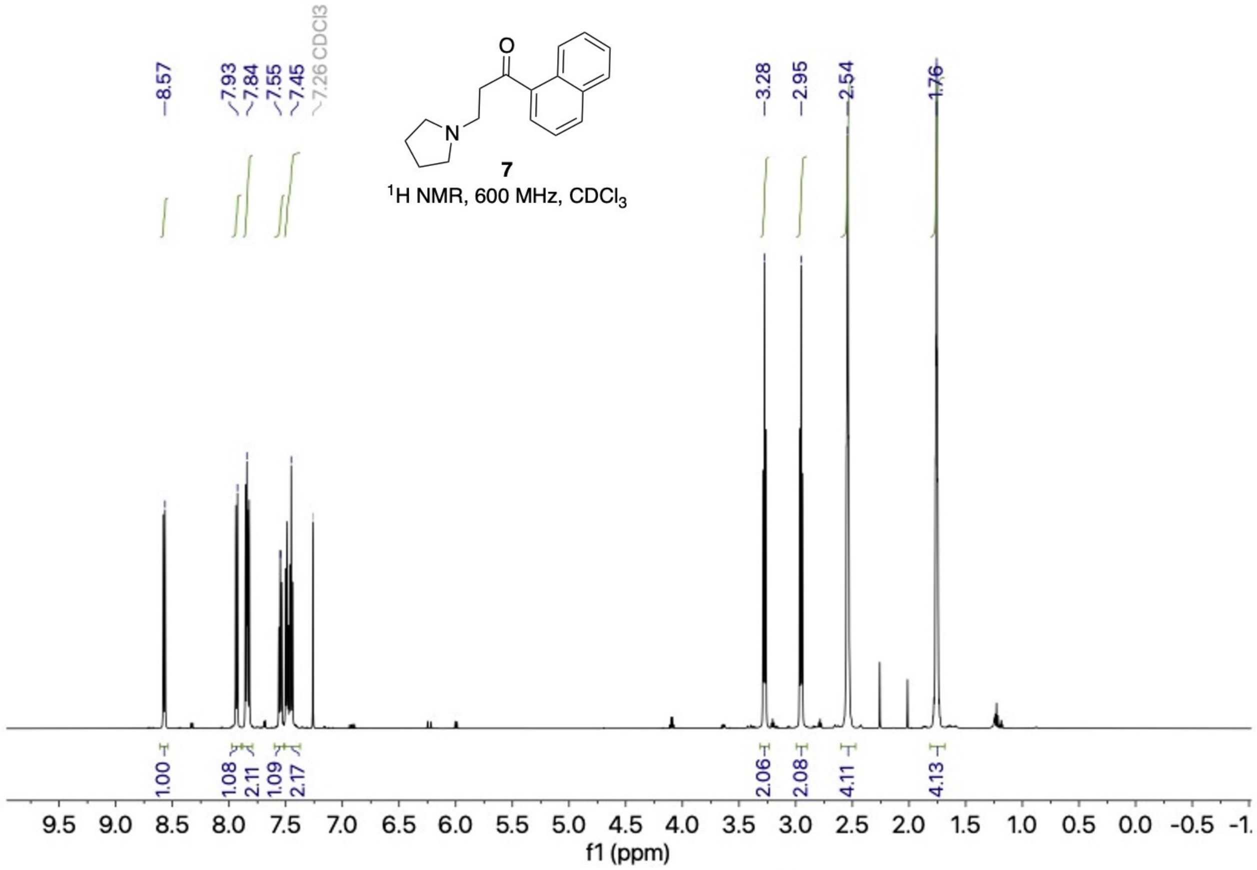
8.57
7.93
7.84
7.55
7.45
7.26 CDCl3
3.28
2.95
2.54
1.76
7
1H NMR, 600 MHz, CDCl3
1.00
1.08
2.11
1.09
2.17
2.06
2.08
4.11
4.13
f1 (ppm)

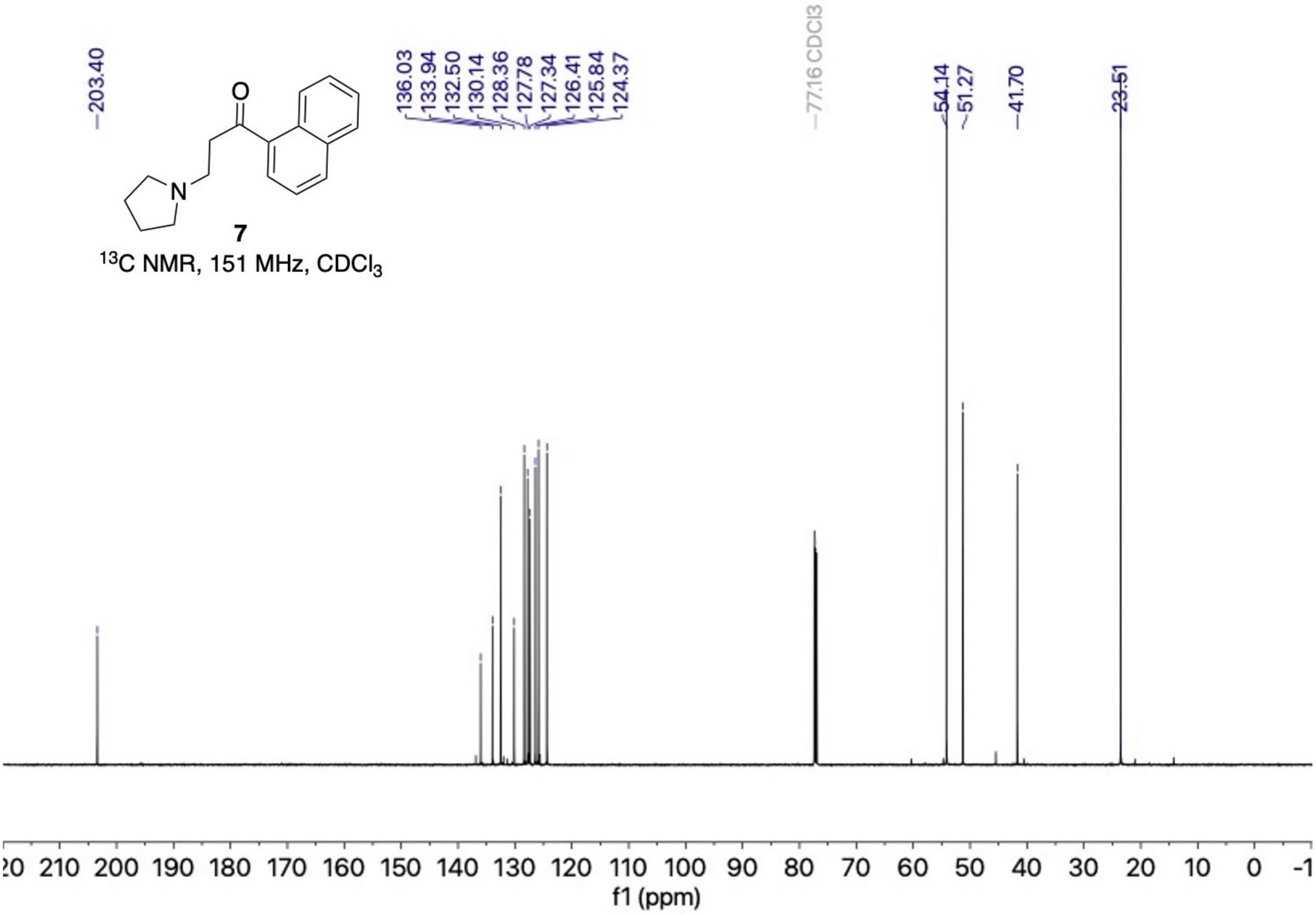
203.40
136.03
133.94
132.50
130.14
128.36
127.78
127.34
126.41
125.84
124.37
77.16 CDCl3
54.14
51.27
41.70
23.51
7
13C NMR, 151 MHz, CDCl3
f1 (ppm)

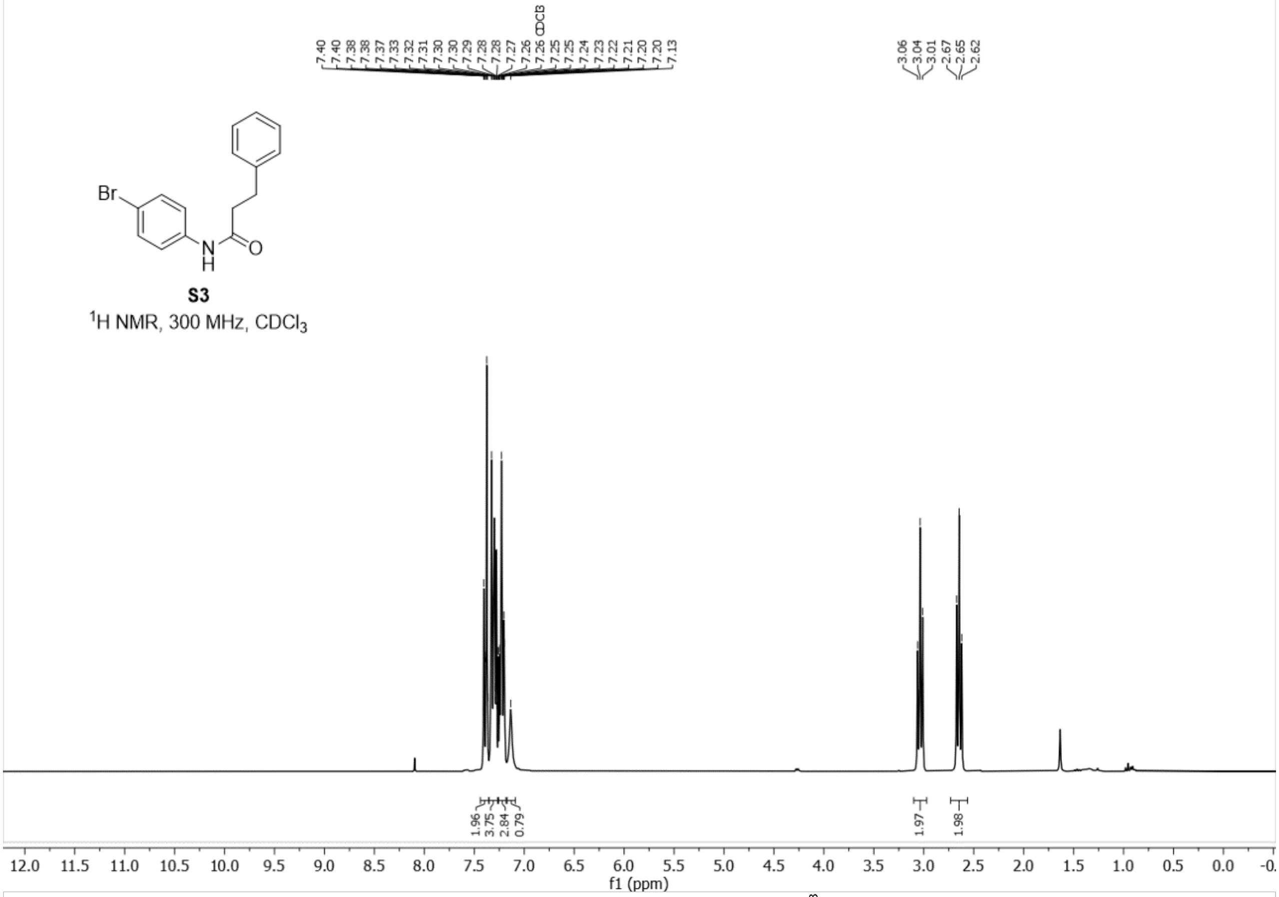
7.40
7.40
7.38
7.38
7.37
7.33
7.32
7.31
7.30
7.30
7.29
7.28
7.28
7.28
7.27
7.26
7.26 CDCl3
7.25
7.25
7.24
7.23
7.22
7.21
7.20
7.20
7.13
3.06
3.04
3.01
2.67
2.65
2.62
Br
N
H
O
S3
^{1}H NMR, 300 MHz, CDCl$_3$
1.96
3.75
2.84
0.79
1.97
1.98
f1 (ppm)

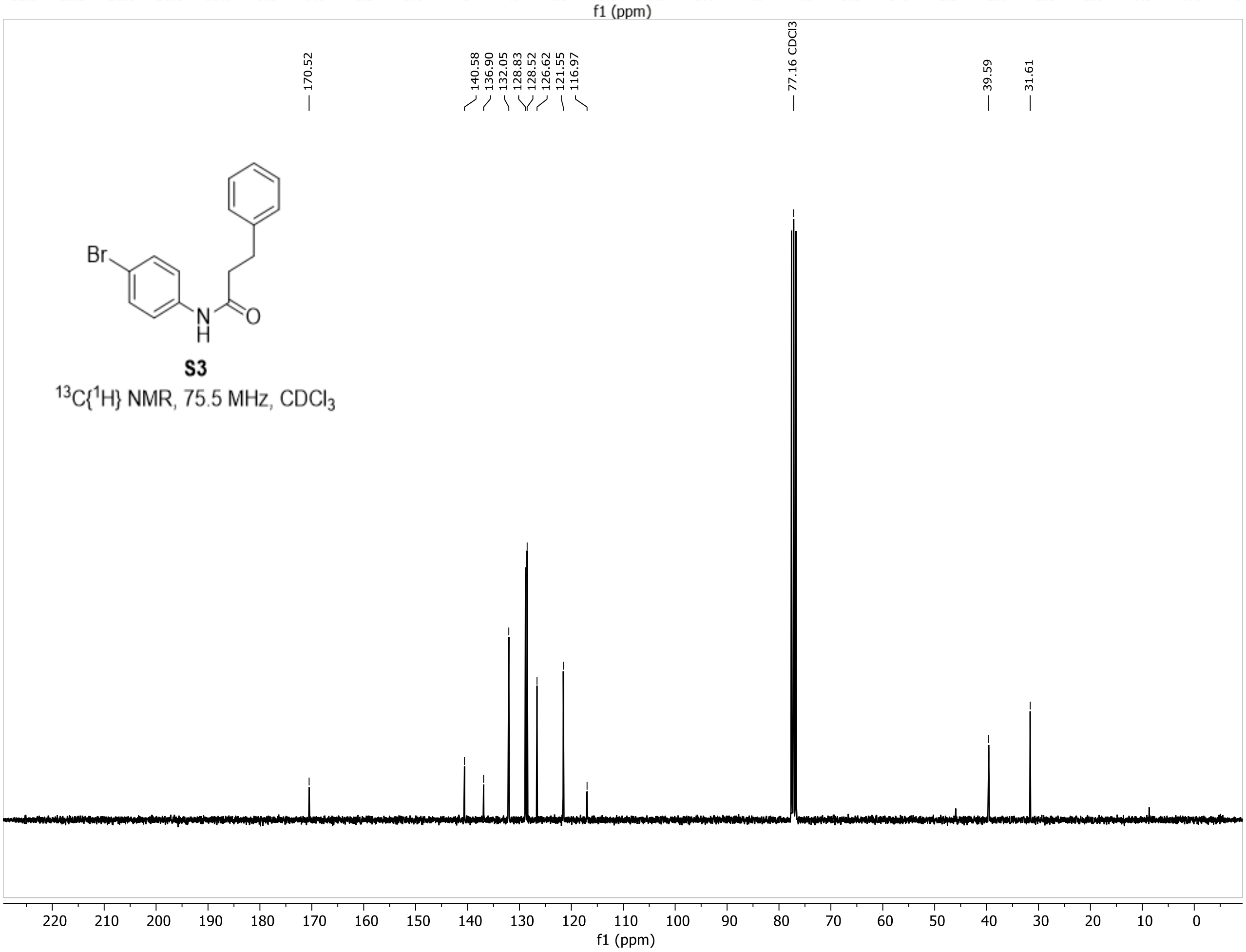
170.52
140.58
136.90
132.05
128.83
128.52
126.62
121.55
116.97
77.16 CDCl3
39.59
31.61
Br
N
H
O
S3
^{13}C{^{1}H} NMR, 75.5 MHz, CDCl$_3$
f1 (ppm)

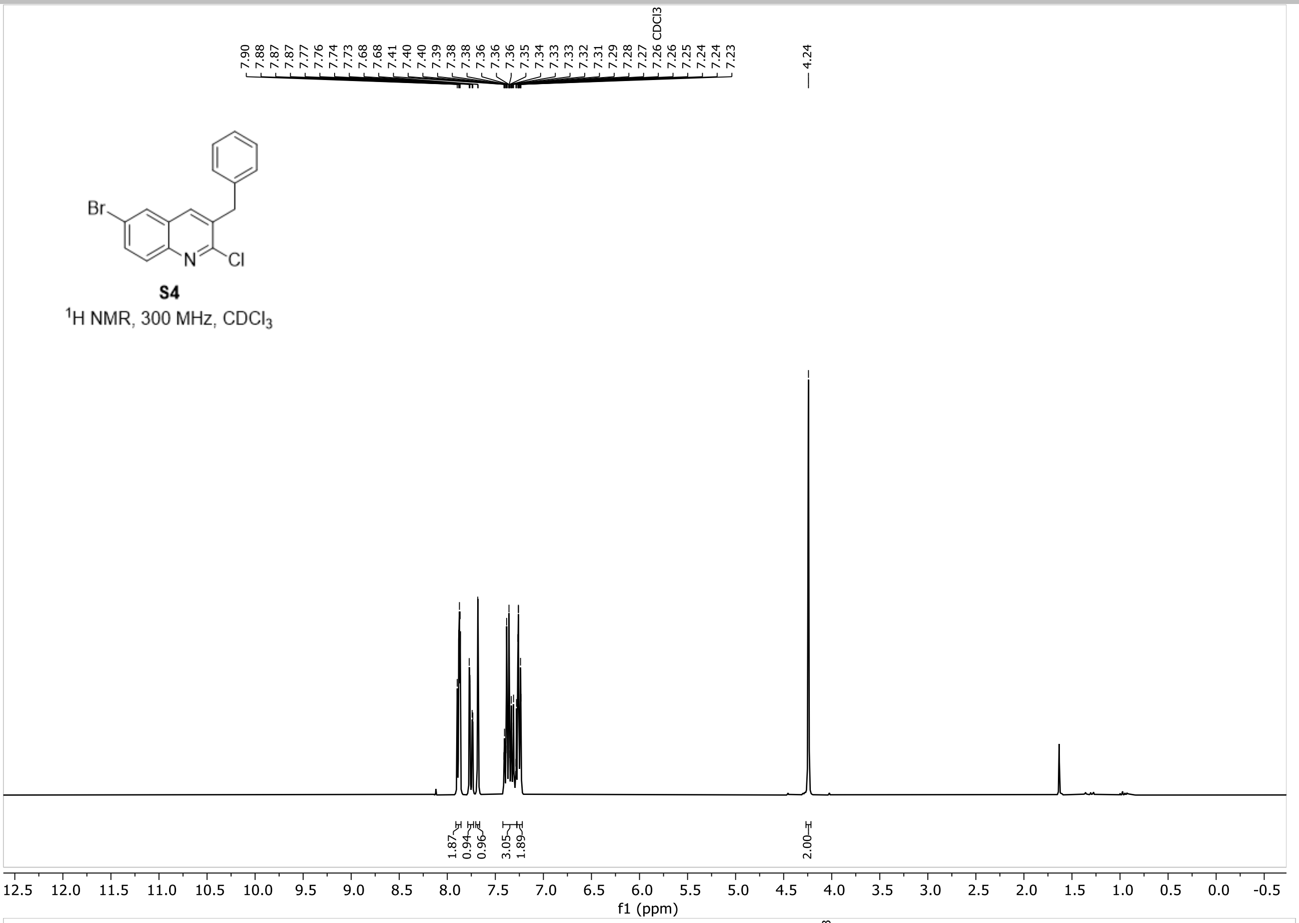
7.90 7.88 7.87 7.87 7.77 7.76 7.74 7.73 7.68 7.68 7.41 7.40 7.40 7.39 7.38 7.38 7.36 7.36 7.36 7.35 7.34 7.33 7.33 7.32 7.31 7.29 7.28 7.27 7.26 CDCl3 7.26 7.25 7.24 7.24 7.23
4.24
Br
N
Cl
S4
^{1}H NMR, 300 MHz, CDCl$_3$
1.87 0.94 0.96 3.05 1.89
2.00
f1 (ppm)

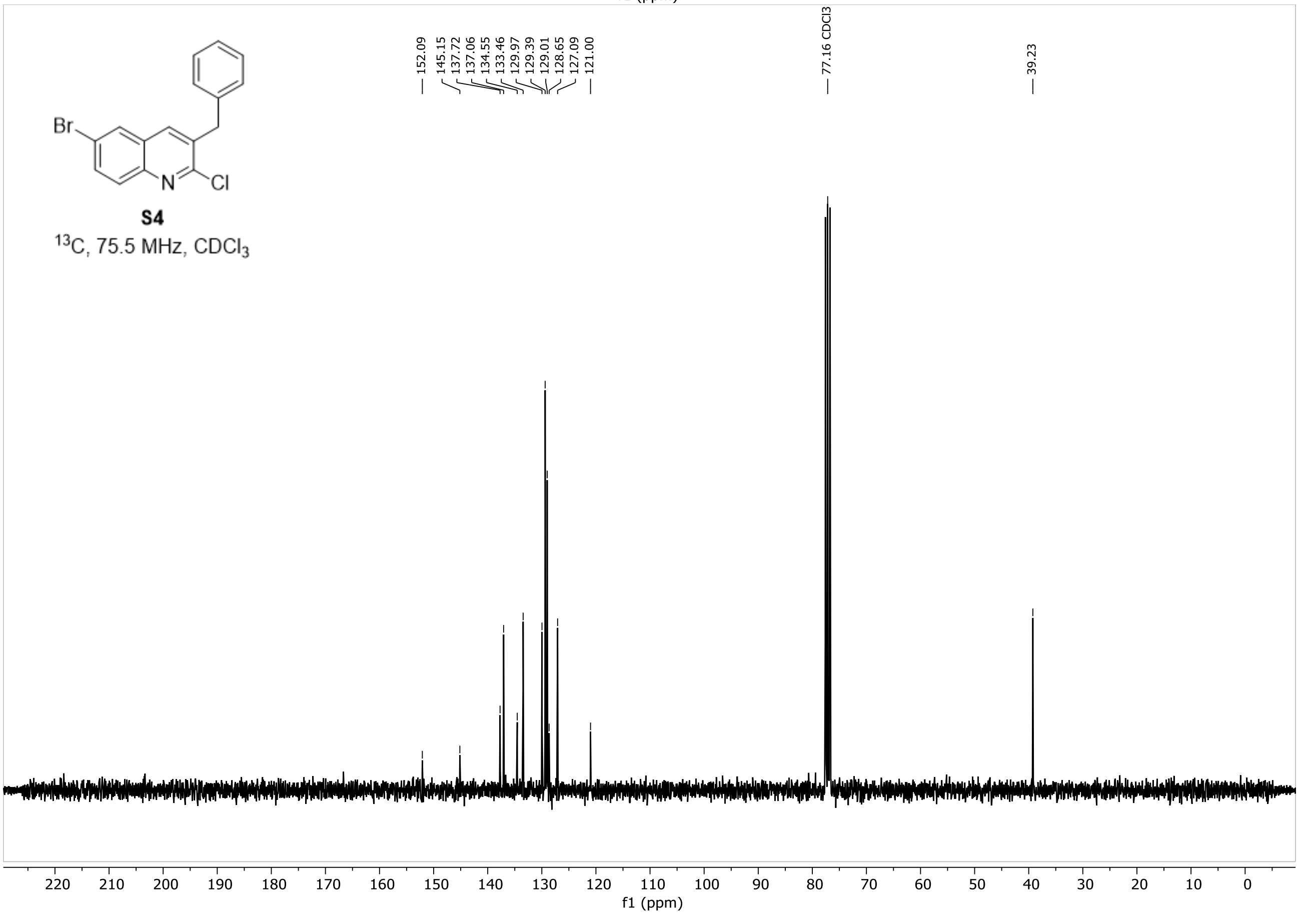
152.09 145.15 137.72 137.06 134.55 133.46 129.97 129.39 129.01 128.65 127.09 121.00
77.16 CDCl3
39.23
Br
N
Cl
S4
^{13}C, 75.5 MHz, CDCl$_3$
f1 (ppm)

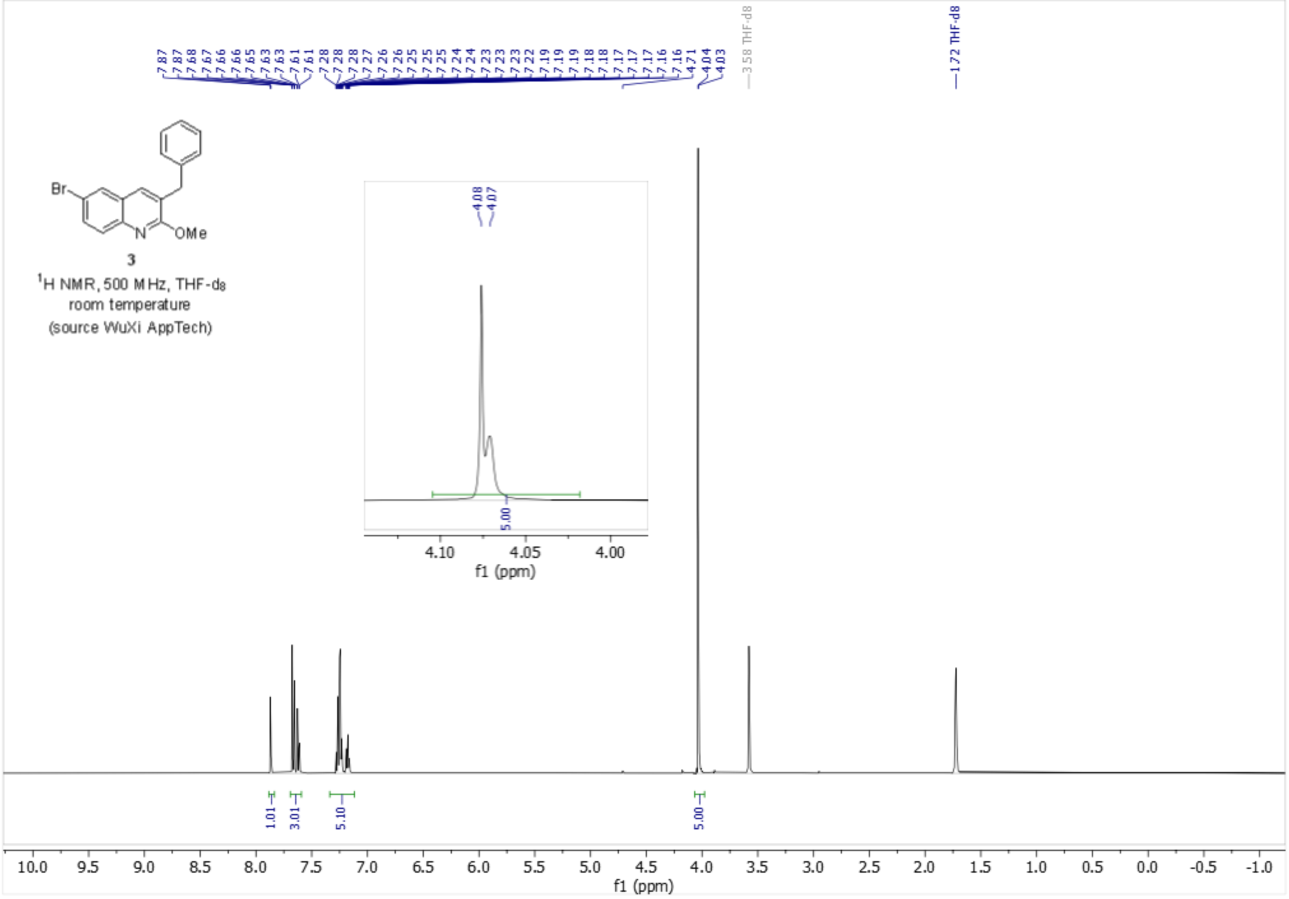
Br
N
OMe
3
1H NMR, 500 MHz, THF-d8
room temperature
(source WuXi AppTech)
3.58 THF-d8
1.72 THF-d8
4.08
4.07
5.00
1.01
3.01
5.10
5.00
f1 (ppm)

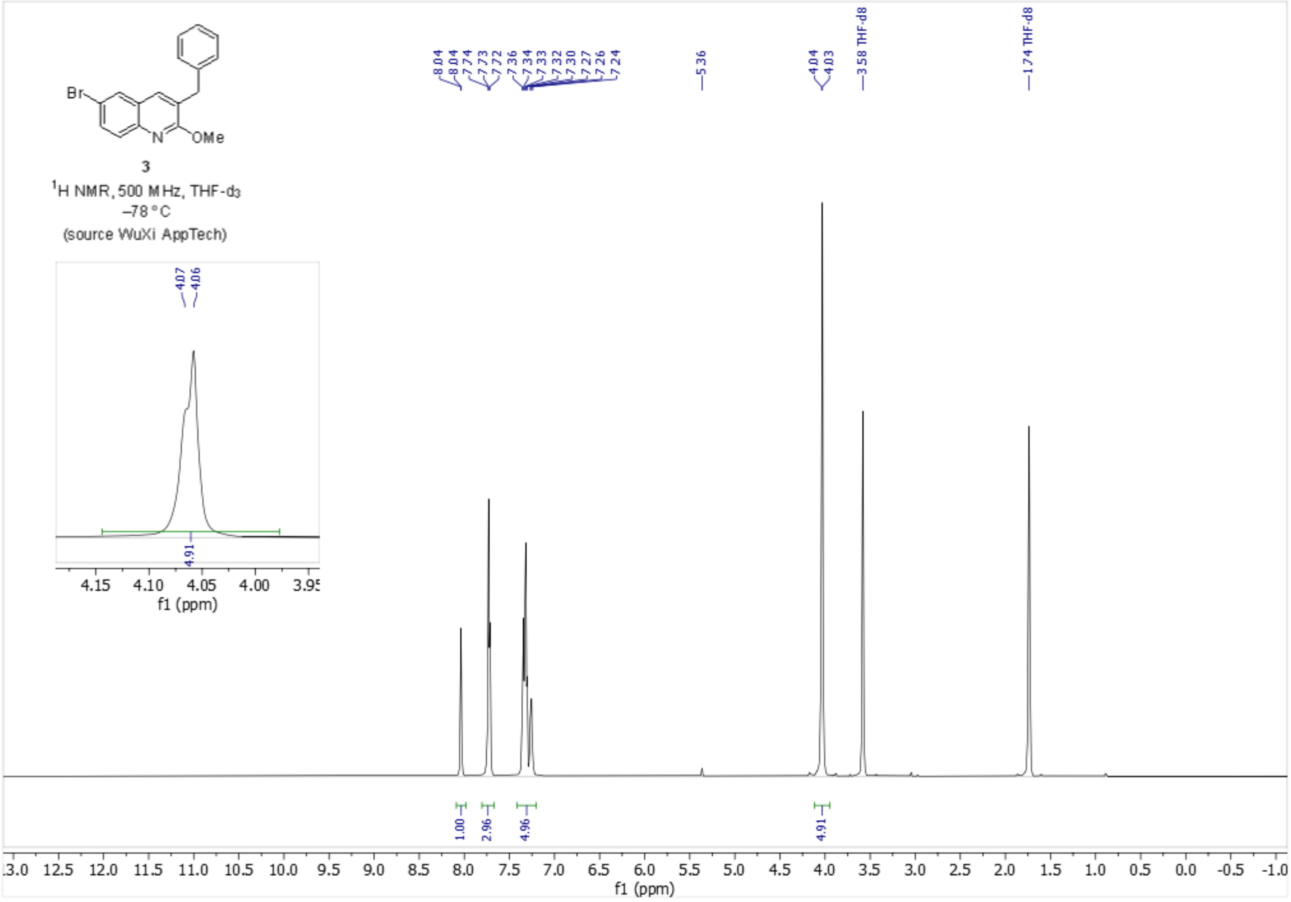
Br
N
OMe
3
1H NMR, 500 MHz, THF-d3
–78 °C
(source WuXi AppTech)
8.04
8.04
7.74
7.73
7.72
5.36
4.04
4.03
3.58 THF-d8
1.74 THF-d8
4.07
4.06
4.91
1.00
2.96
4.96
4.91
f1 (ppm)

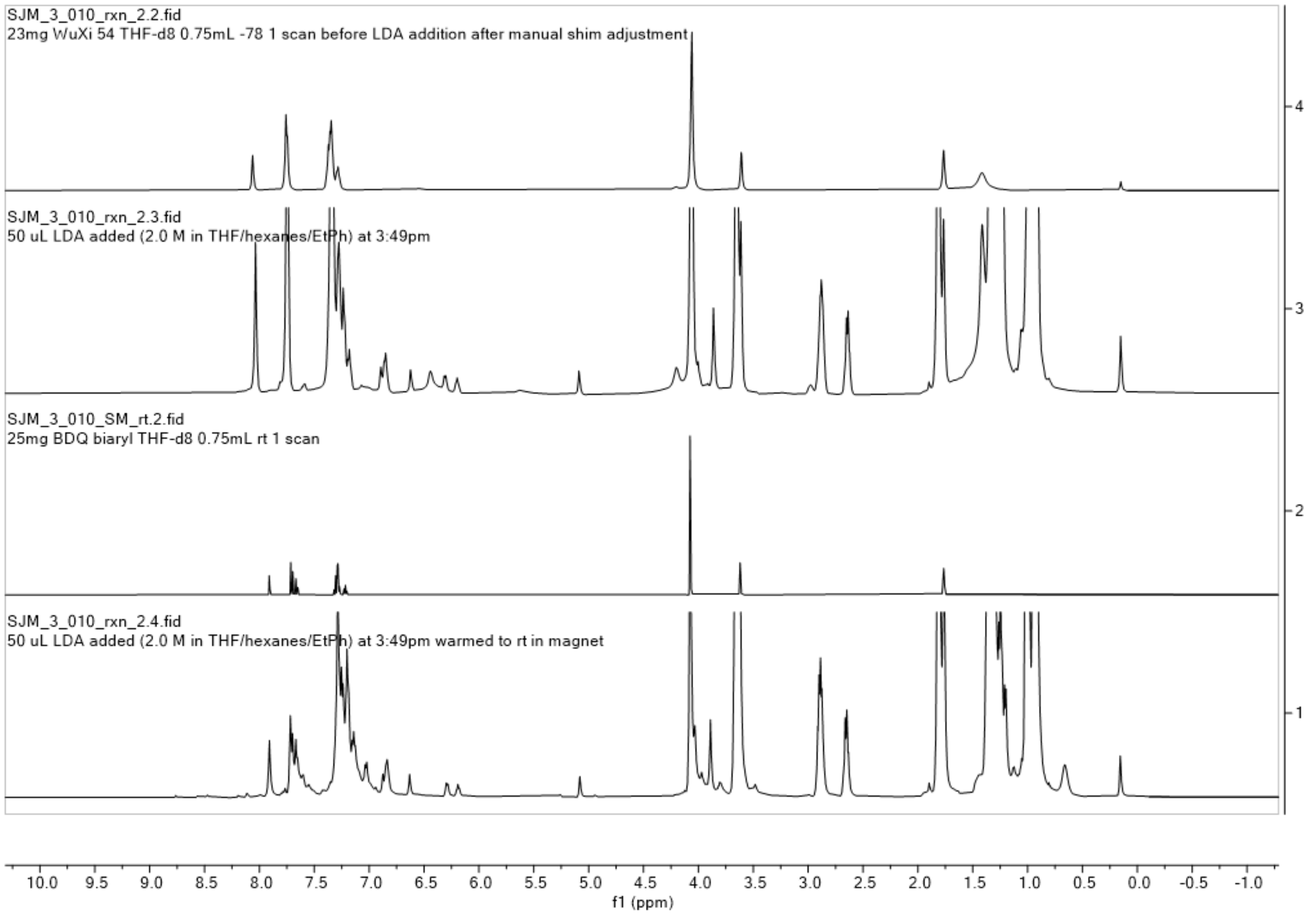
SJM_3_010_rxn_2.2.fid
23mg WuXi 54 THF-d8 0.75mL -78 1 scan before LDA addition after manual shim adjustment
SJM_3_010_rxn_2.3.fid
50 uL LDA added (2.0 M in THF/hexanes/EtPh) at 3:49pm
SJM_3_010_SM_rt.2.fid
25mg BDQ biaryl THF-d8 0.75mL rt 1 scan
SJM_3_010_rxn_2.4.fid
50 uL LDA added (2.0 M in THF/hexanes/EtPh) at 3:49pm warmed to rt in magnet
f1 (ppm)

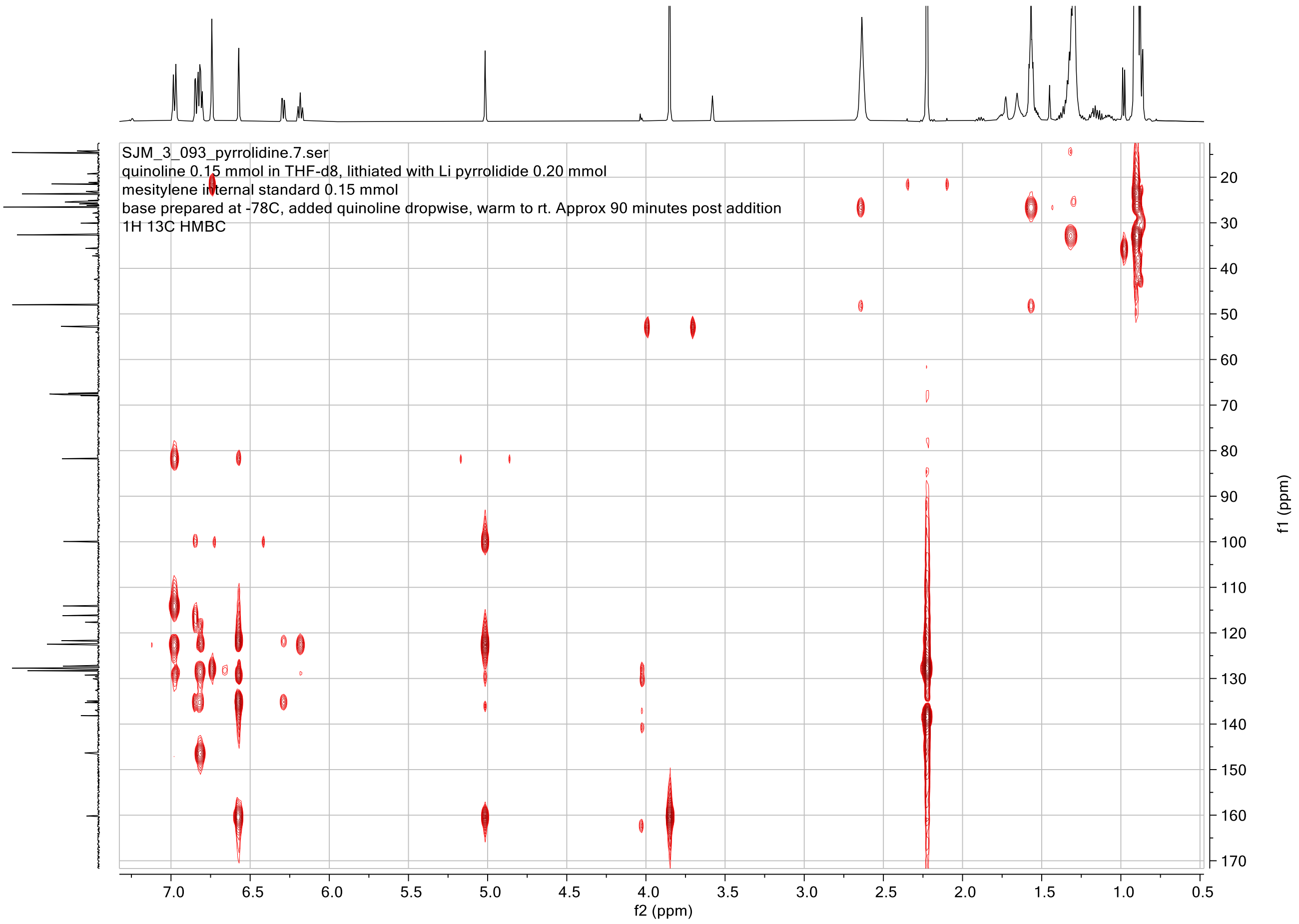
SJM_3_093_pyrrolidine.7.ser
quinoline 0.15 mmol in THF-d8, lithiated with Li pyrrolidide 0.20 mmol
mesitylene internal standard 0.15 mmol
base prepared at -78C, added quinoline dropwise, warm to rt. Approx 90 minutes post addition
1H 13C HMBC
f2 (ppm)
f1 (ppm)

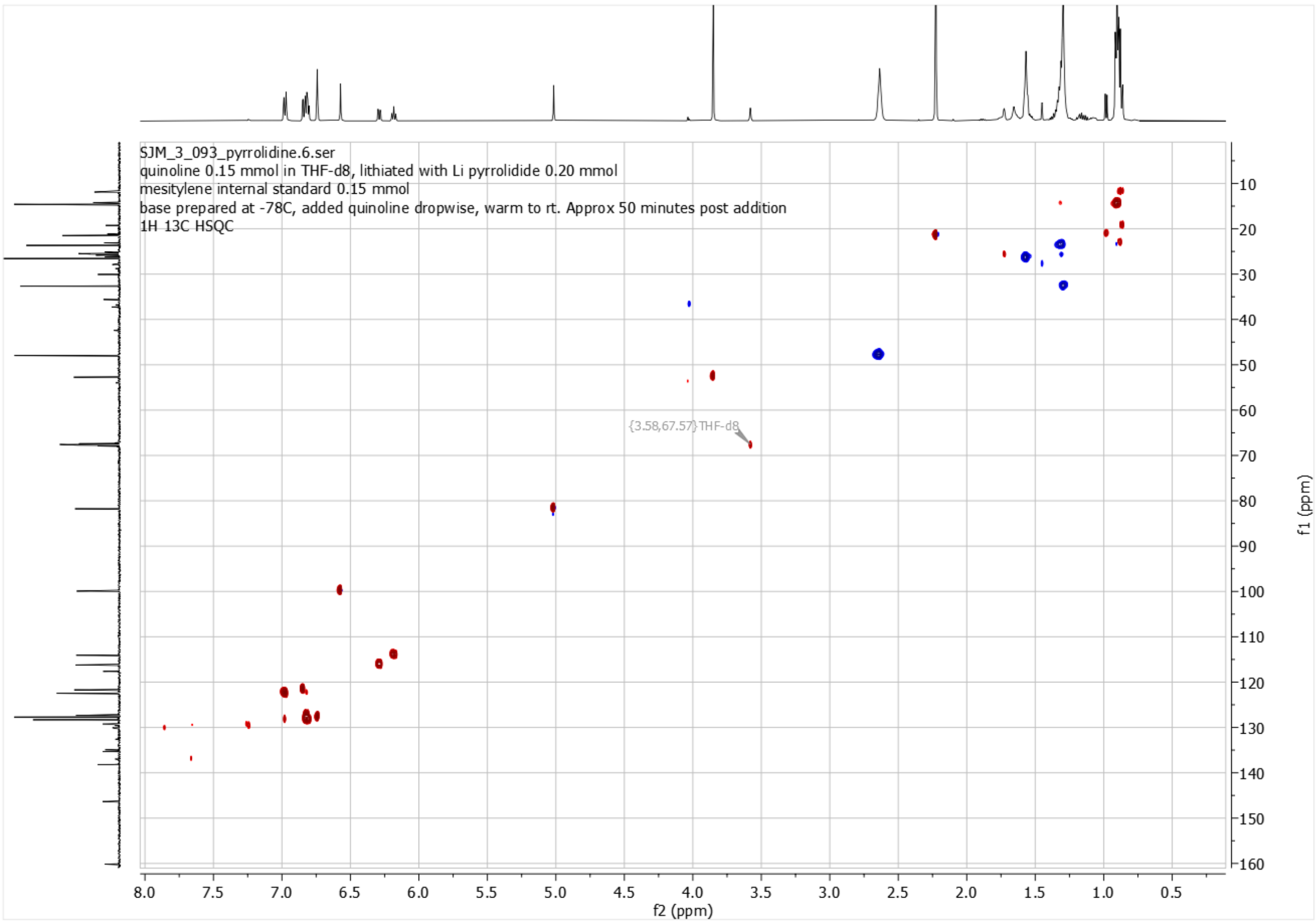

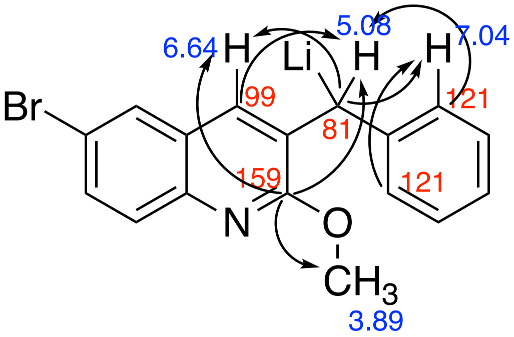

Figure S8. ^{1}H-^{13}C HMBC correlations with arrows illustrating observed correlations between ^{1}H (blue) and ^{13}C (red) resonances.

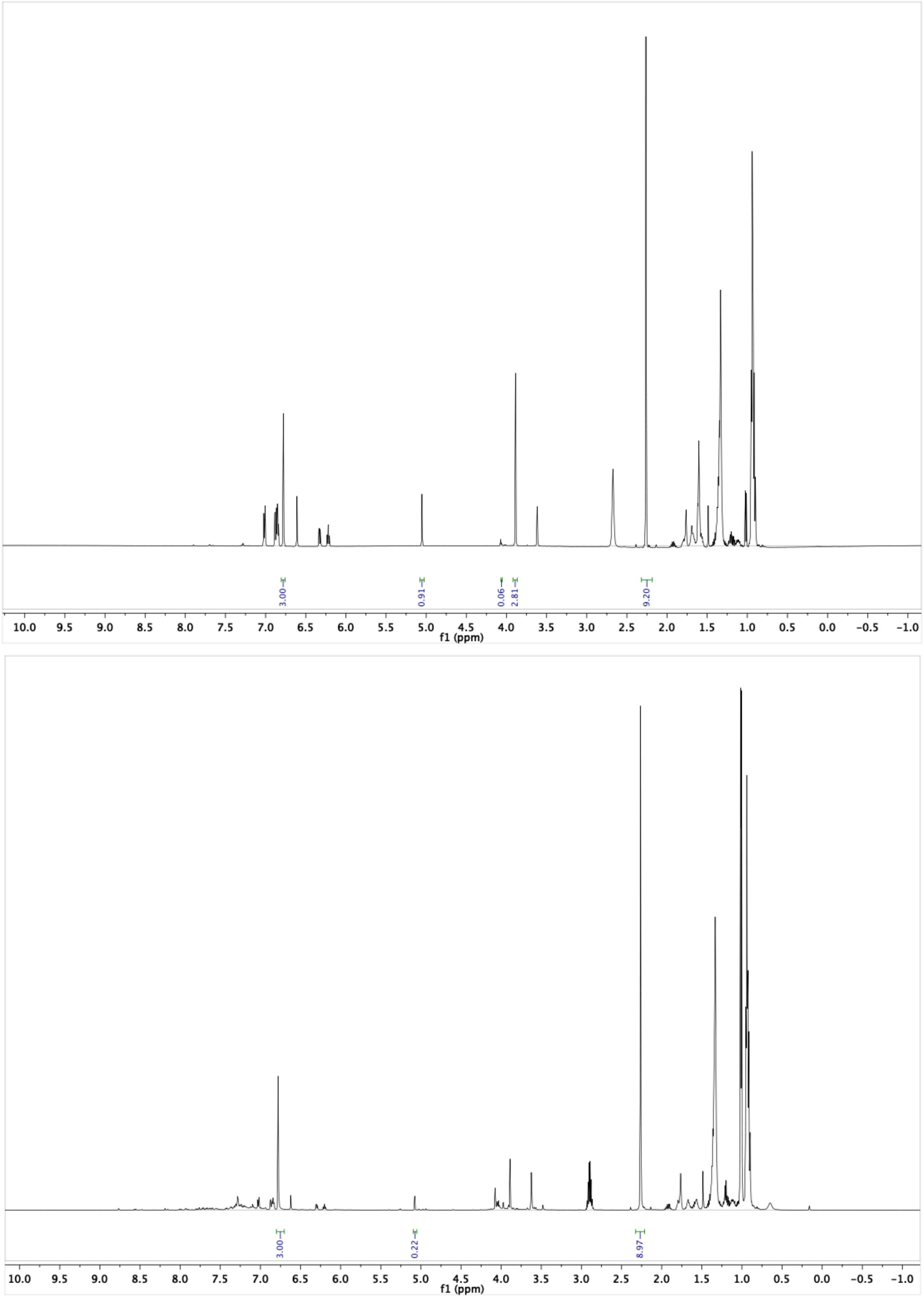

Figure S9. ^{1}H NMR spectrum of (top) lithium pyrrolidide / **3** with mesitylene internal standard showing 91% yield of **3a** with good mass balance and (bottom) lithium diisopropylamide / **3** with mesitylene internal standard showing 22% yield of **3a** with significant undesired reactions occurring.

References

[1] A. F. Burchat, J. M. Chong, N. Nielsen, *J Organomet Chem.* **1997**, *542*, 281–283.
[2] J. F. E. V. Gestel, J. E. G. Guillemont, M. G. Venet, H. J. J. Poignet, L. F. B. Decrane, D. F. J. Vernier, F. C. Odds, WO2004011436A1, **2004.**
[3] F. R. Porstmann, S. Horns, T. Bader, WO 125769A1, **2006**.
[4] F. Zhang, L. Pan, J. Zhu, X. Wang, L. Zhao, Z. Ren, Y. Song, WO 015793 A1, **2017**.
[5] W. Feng, D. Kong, CN 105017147B, **2019**.
[6] S. Sebastian, S. K. Singh, S. Polavarapu, U. Veera, WO 161743 A1, **2020**.
[7] D.-L. Kong, Y. Huang, L.-Y. Ren, W.-H. Feng, *Chin Chem Lett.* **2015**, *26*, 790–792.
[8] L. Wu, Y. Hao, Y. Liu, Q. Wang, *Org. Biomol. Chem.* **2019**, *17*, 6762–6770.
[9] R. Pathak, S. Madapa, S. Batra, *Tetrahedron* **2007**, *63*, 451–460.
[10] C. He, L. Preiss, B. Wang, L. Fu, H. Wen, X. Zhang, H. Cui, T. Meier, D. Yin, *ChemMedChem* **2016,** *12(2),* 106–119.

Appendix: Synthesis of S1 and 3 by WuXi AppTech

Synthesis of 3-(dimethylamino)-1-(naphthalen-1-yl)propan-1-one hydrochloride (S1) by WuXi AppTech:

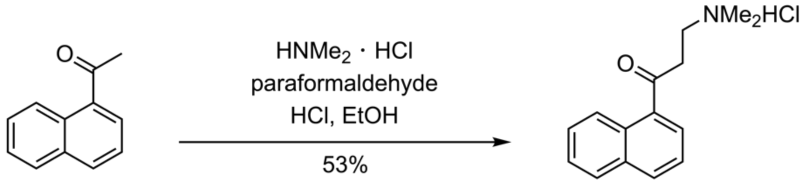

S1

Scheme S12: Synthesis of 3-(dimethylamino)-1-(naphthalen-1-yl)propan-1-one hydrochloride (**S1**) by WuXi AppTech.

Ketone salt **S1** was prepared from 1-acetonapthone according to a literature procedure.[6] 1-acetonaphthone (80 g, 470 mmol, 1 equiv), dimethylamine hydrochloride (57.5 g, 705 mmol, 1.5 equiv), and paraformaldehyde (30 g, 1.5 equiv) were dissolved in ethanol (140 mL). Concentrated hydrochloric acid (12 M, 10 mL, 120 mmol, 0.25 equiv) was added and the solution was warmed to 80 °C and stirred for 30 h. Upon completion, the reaction mixture was concentrated under reduced pressure to remove the majority of the ethanol, then cooled to 4–5 °C for 4 h to precipitate **S1**. The resulting suspension was filtered and the filter cake was washed with ethanol (2 x 50 mL) and dried in vacuo to afford **S1** as a white solid (68 g, 53%, 97% purity). The analytical data were consistent with those reported in the literature.[7]

Synthesis of 3-benzyl-6-bromo-2-methoxyquinoline (3) by WuXi AppTech:

Quinoline **3** was prepared starting from 3-phenylpropanoic acid (Scheme S13).[7] The acid **S5** was chlorinated to form acyl chloride **S2**, which was directly reacted with 4-bromoaniline to yield compound **S3**. A Vilsmeier-Haack reaction and subsequent condensation then furnished quinoline **S4**. After nucleophilic substitution with sodium methoxide and recrystallization, the desired product **3** was obtained in 62% overall yield from **S5** and 99% purity.

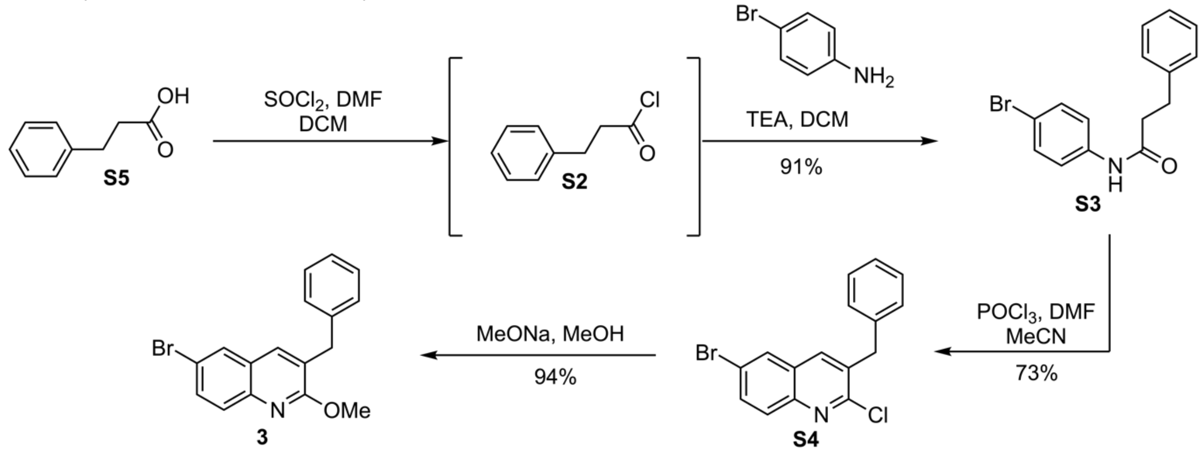

Scheme S13: Synthesis of 3-benzyl-6-bromo-2-methoxyquinoline (**3**) by WuXi AppTech.

N-(4-Bromophenyl)-3-phenylpropanamide (S3)

3-phenylpropanoic acid (100 g, 666 mmol, 1.0 equiv) and DMF (4.87 g, 66.6 mmol, 5.1 mL, 0.1 equiv) were dissolved in CH_2Cl_2 (1000 mL). $SOCl_2$ (158 g, 1.33 mol, 96.6 mL, 2.0 equiv) was added dropwise over 1 h at 0 °C. The solution was warmed to 20 °C and stirred for a further 2 h. During stirring, periodic 0.5 mL aliquots were removed and quenched with methanol (1 mL) for monitoring by TLC and ^{1}H NMR spectroscopy. Upon completion, the reaction was concentrated under reduced pressure to give **S2** (113 g, 100%) as a colorless oil. This material was used directly in the next step without further purification. 4-Bromoaniline (121 g, 704 mmol, 1.05 equiv) was dissolved in CH_2Cl_2 (1000 mL) and triethylamine (81 g, 804 mmol, 112 mL, 1.2 equiv) under nitrogen atmosphere. The solution was cooled to 0 °C and 3-phenylpropanoyl chloride (**S2**, 113 g, 670 mmol, 1.0 equiv) was added dropwise over 1 h. The solution was allowed to warm to 20 °C and stirred for an additional 1 hr. Ice water (500 mL) was then added to quench and a white solid precipitated upon addition. The resultant suspension was filtered to obtain the filter cake as a white solid. The filtrate was then partitioned and the aqueous phase was extracted with CH_2Cl_2 (2 x 500 mL). The combined organic layers were washed with 1 M aqueous HCl (2 x 500 mL) and saturated aqueous NaCl (2 x 300 mL), then dried over Na_2SO_4. The mixture was filtered and concentrated under reduced pressure until significant white precipitate formed. The suspension was then filtered to obtain the filter cake as a white solid. The combined filter cakes were dried in vacuo to yield **S3** (185 g, 91% yield, 100% purity). The analytical data were consistent with those reported in the literature.[7,8]

3-Benzyl-6-bromo-2-chloroquinoline (S4):

DMF (48 g, 658 mmol, 51 mL, 4.0 equiv) was cooled to 0 °C under nitrogen atmosphere. $POCl_3$ (202 g, 1.32 mol, 122 mL, 8.0 equiv) was added dropwise while stirring. After complete addition, the mixture was stirred for 1 h at 20 °C followed by addition of MeCN (150 mL) and **S3** (50 g, 164 mmol, 1.0 equiv). The reaction mixture was heated to 80 °C and stirred for a further 36 h. After completion, the reaction mixture was cooled to room temperature and added slowly to water (2000 mL), resulting in precipitation of **S4**. The suspension was filtered and the filter cake was washed with cold methanol (2 x 50 mL). The filter cake was then dried in vacuo to afford **S4** (41 g, 73%, 98% purity) as an off-white solid. The analytical data were consistent with those reported in the literature.[7,9]

3-Benzyl-6-bromo-2-methoxyquinoline (3):

To a suspension of **S4** (60 g, 177 mmol, 1.0 equiv) in MeOH (300 mL) was added a solution of sodium methoxide in methanol (5 M, 177 mL, 5 equiv). The reaction mixture was heated to 80 °C and stirred for 8 h under nitrogen atmosphere. The reaction mixture was then cooled to 20 °C and filtered, and the filter cake was washed with cold methanol (2 x 50 mL). The filter cake was added to water (200 mL) and the resulting suspension was stirred for 30 min at 20 °C. The suspension was then filtered and the filter cake was washed with water (100 mL) and dried in vacuo to yield **3** (55 g, 94% yield, 99% purity) as an off-white solid. The analytical data were consistent with those reported in the literature.[7,9]

^{1}H NMR Spectra for WuXi AppTech Products and Intermediates

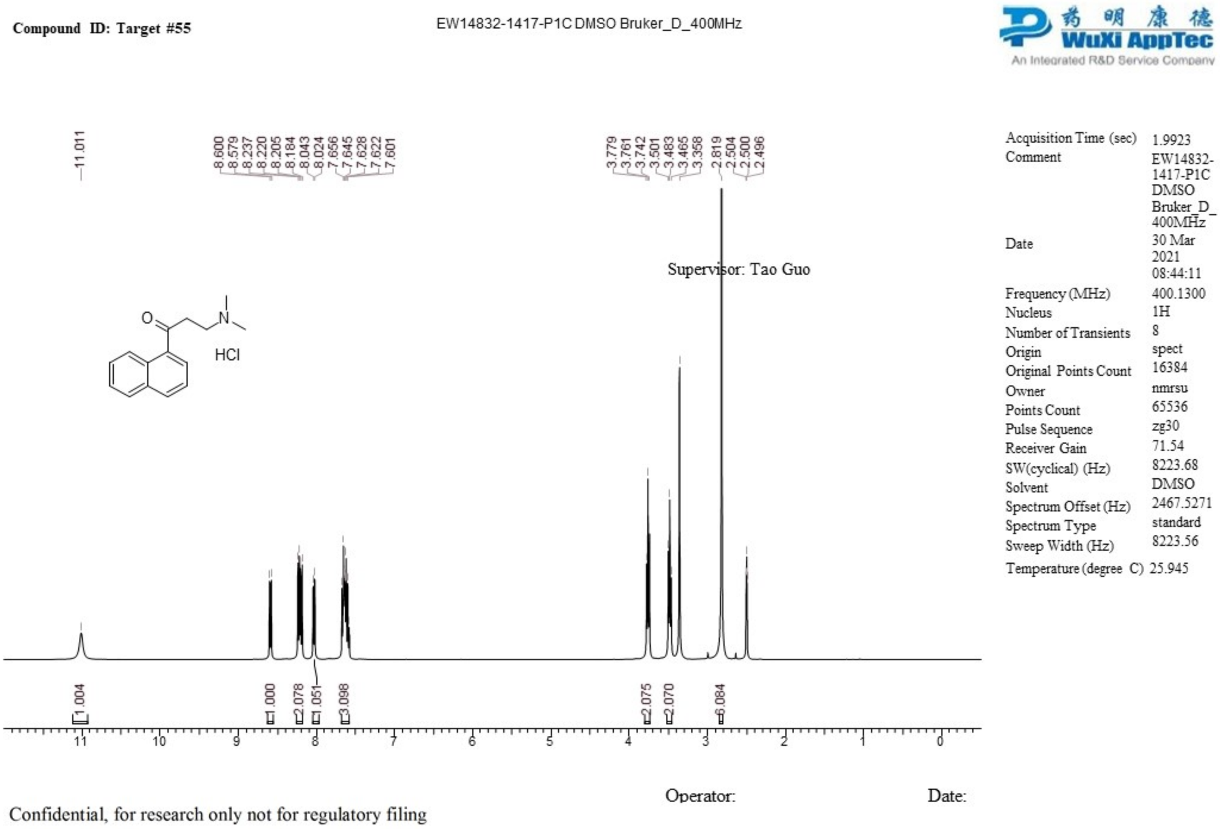

Figure S10. ^{1}H NMR spectrum of **S1**.

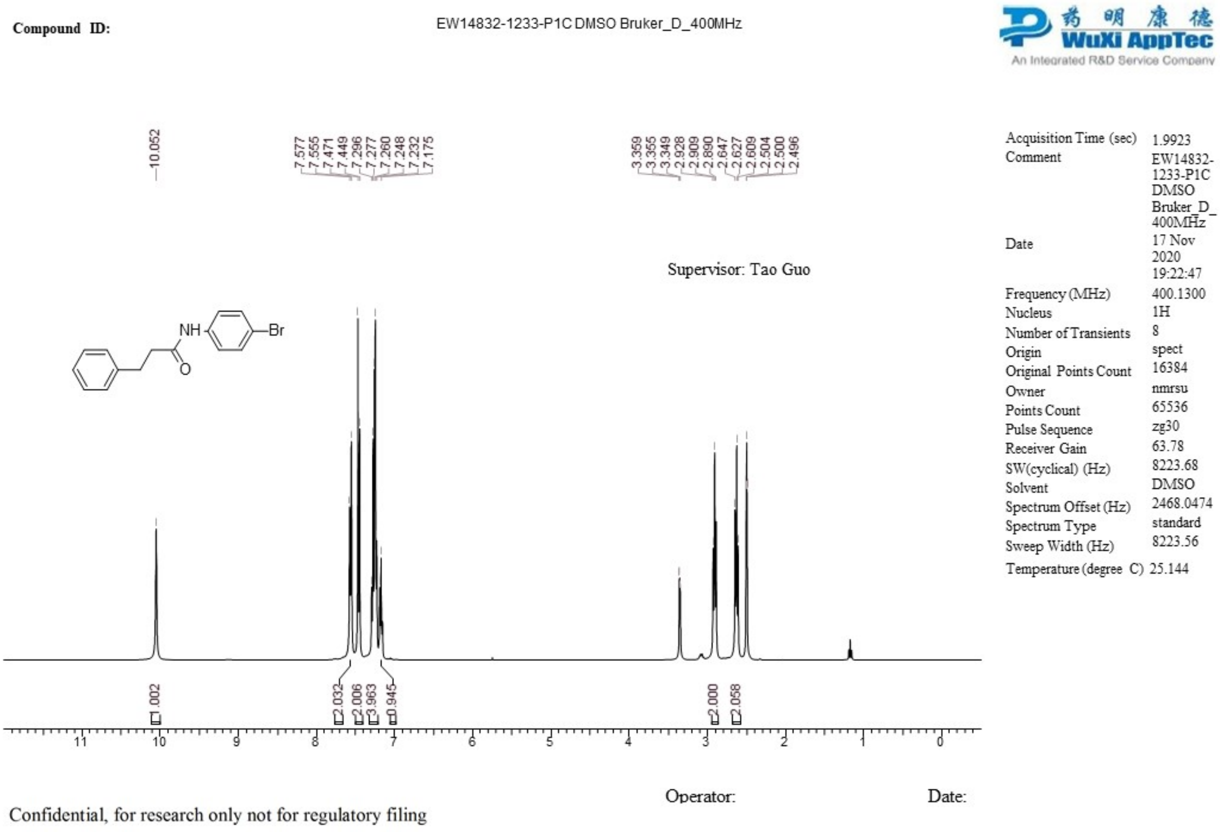

Figure S11. ^{1}H NMR spectrum of **S3**.

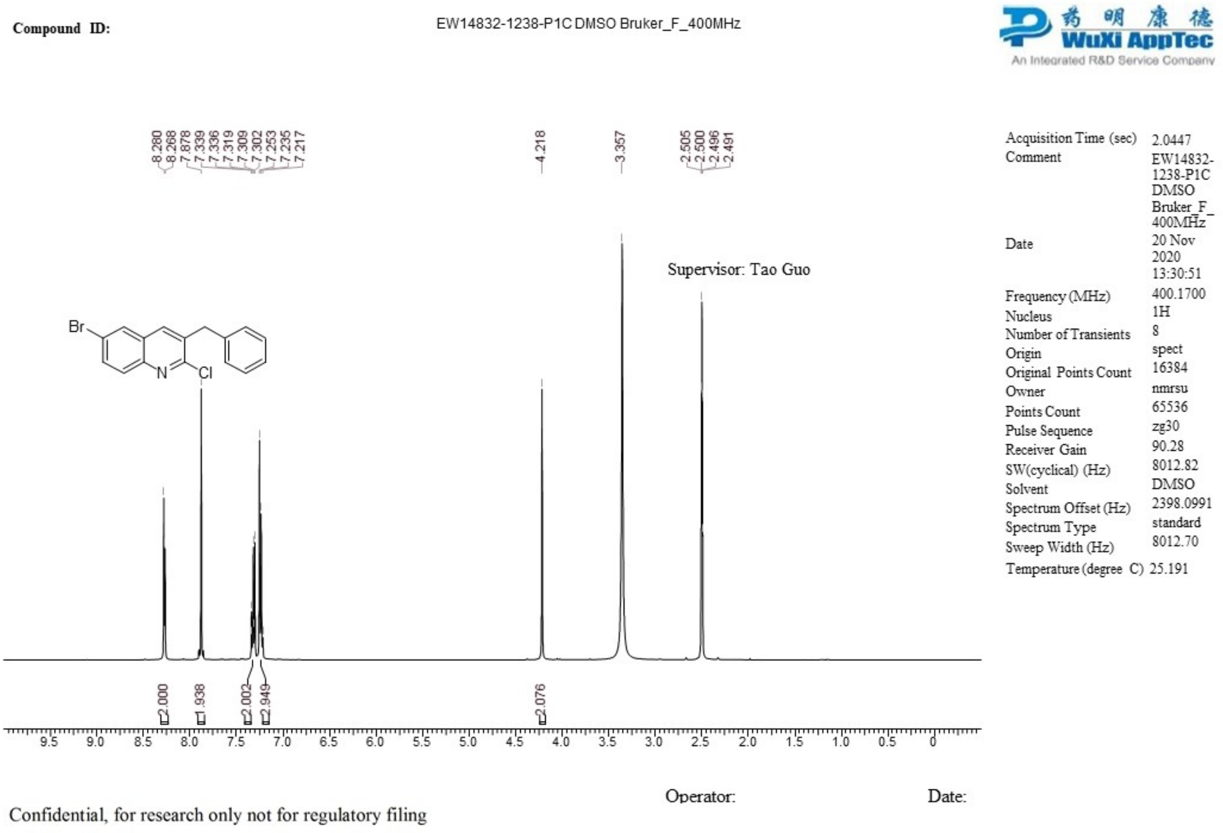

Figure S12. ^{1}H NMR spectrum of **S4**.

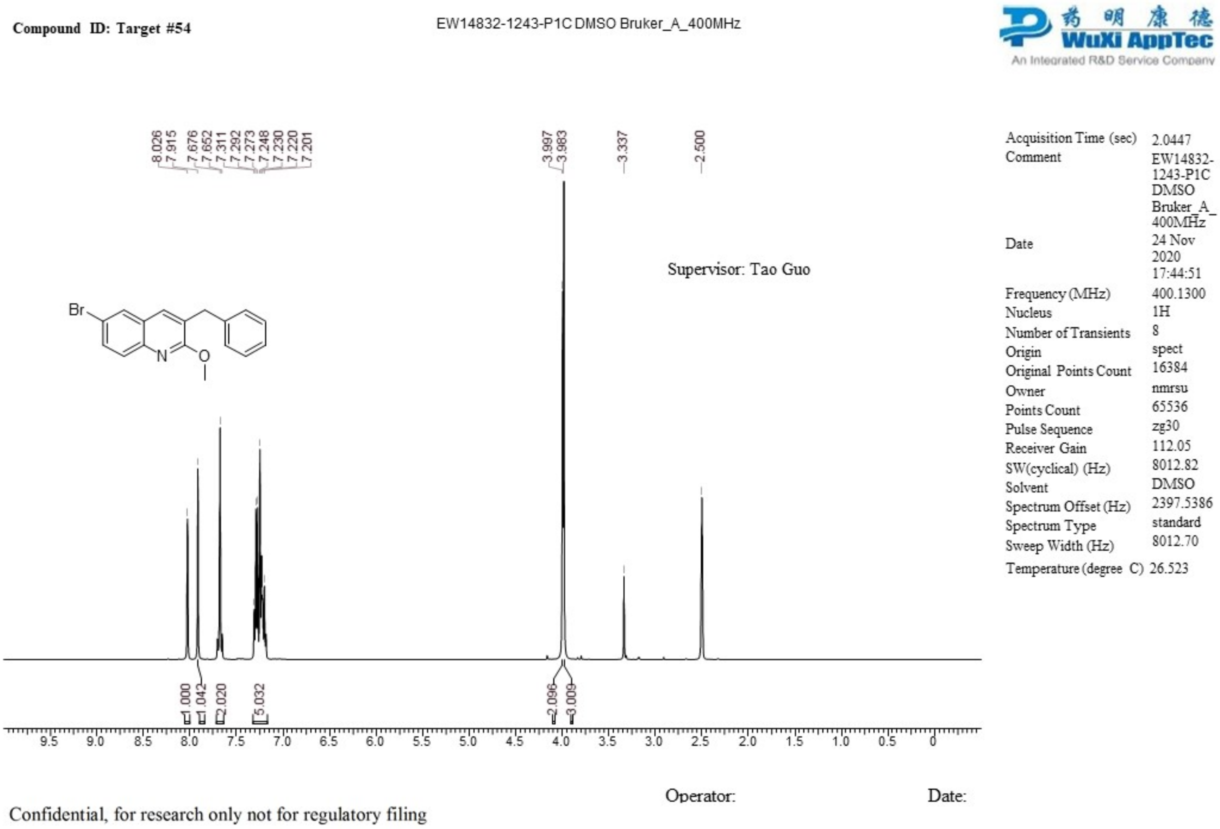

Figure S13. ^{1}H NMR spectrum of **3**.
